# Garlic exosome-like nanoparticles reverse high-fat diet induced obesity via the gut/brain axis

**DOI:** 10.7150/thno.65427

**Published:** 2022-01-01

**Authors:** Kumaran Sundaram, Jingyao Mu, Anil Kumar, Jyotirmaya Behera, Chao Lei, Mukesh K Sriwastva, Fangyi Xu, Gerald W Dryden, Lifeng Zhang, ShaoYu Chen, Jun Yan, Xiang Zhang, Juw Won Park, Michael L Merchant, Neetu Tyagi, Yun Teng, Huang-Ge Zhang

**Affiliations:** 1 James Graham Brown Cancer Center, Department of Microbiology & Immunology, University of Louisville, KY 40202, USA.; 2 Department of Physiology, University of Louisville School of Medicine, Louisville, KY 40202, USA.; 3 Department of Medicine, University of Louisville, Louisville, KY 40202, USA.; 4 Department of Pharmacology and Toxicology, University of Louisville, Louisville, KY 40202, USA.; 5 Department of Computer Engineering and Computer Science, University of Louisville, KY 40202, USA.; 6 KBRIN Bioinformatics Core, University of Louisville, Louisville, KY 40202, USA.; 7 Kidney Disease Program and Clinical Proteomics Center, University of Louisville, Louisville, KY 40202, USA.; 8 Robley Rex Veterans Affairs Medical Center, Louisville, KY 40206, USA.

**Keywords:** Garlic derived exosome-like nanoparticles, phosphatidic acid, microglial cells, BASP1, c-Myc, IDO1, AHR, STING, mitochondria, gut/brain axis, brain inflammation, obesity.

## Abstract

**Background:** Obesity is becoming a global epidemic and reversing the pathological processes underlying obesity and metabolic co-morbidities is challenging. Obesity induced chronic inflammation including brain inflammation is a hallmark of obesity via the gut-brain axis. The objective of this study was to develop garlic exosome-like nanoparticles (GaELNs) that inhibit systemic as well as brain inflammatory activity and reverse a HFD induced obesity in mice.

**Methods:** GELNs were isolated and administrated orally into HFD fed mice. GaELNs were fluorescent labeled for monitoring their *in vivo* trafficking route after oral administration and quantified the number particles in several tissues. The brain inflammation was determined by measuring inflammatory cytokines by ELISA and real-time PCR. Mitochondrial membrane permeability of microglial cells was determined using JC-10 fluorescence dye. The *in vivo* apoptotic cell death was quantified by TUNEL assay. The brain metabolites were identified and quantified by LC-MS analysis. Memory function of the mice was determined by several memory functional analysis. The effect of GaELNs on glucose and insulin response of the mice was determined by glucose and insulin tolerance tests. c-Myc localization and interaction with BASP1 and calmodulin was determined by confocal microscopy.

**Results:** Our results show that GaELNs is preferentially taken up microglial cells and inhibits the brain inflammation in HFD mice. GaELN phosphatidic acid (PA) (36:4) is required for the uptake of GaELNs via interaction with microglial BASP1. Formation of the GaELNs/BASP1 complex is required for inhibition of c-Myc mediated expression of STING. GaELN PA binds to BASP1, leading to inhibition of c-Myc expression and activity through competitively binding to CaM with c-Myc transcription factor. Inhibition of STING activity leads to reducing the expression of an array of inflammatory cytokines including IFN-γ and TNF-α. IFN-γ induces the expression of IDO1, which in turn the metabolites generated as IDO1 dependent manner activate the AHR pathway that contributes to developing obesity. The metabolites derived from the GaELNs treated microglial cells promote neuronal differentiation and inhibit mitochondrial mediated neuronal cell death. GaELNs treated HFD mice showed improved memory function and increased glucose tolerance and insulin sensitivity in these mice.

**Conclusion**: Collectively, these results demonstrate how nanoparticles from a healthy diet can inhibit unhealthy high-fat diet induced brain inflammation and reveal a link between brain microglia/diet to brain inflammatory disease outcomes via diet-derived exosome-like nanoparticles.

## Introduction

Chronic non-resolving inflammation with widespread microglial activation is a defining feature of central nervous system (CNS) diseases, including high-fat diet (HFD) induced obesity and metabolic syndrome. Glial cells, which constitute more than 50% of the mass of the CNS, have been shown to be involved in body weight homeostasis and obesity 1-4. Among glial cells, microglia cells contribute to the sculpting of neural circuits through the promotion of neural cell genesis, patterning of synaptic connections, and removal of apoptotic cells 5-8. They also respond vigorously to both chronic disease and acute insults affecting the CNS 9, 10. Targeted delivery of therapeutic agents to microglia for treatment of the CNS is needed but highly challenging.

A healthy diet can prevent unhealthy diet induced chronic inflammation and associated brain damage 11. However, to date the medical community does not fully understand how a healthy diet can prevent or reduce the harmful effects of an unhealthy diet, such as a HFD induced systemic inflammation including brain inflammation. A better mechanistic understanding of dietary effects is essential for the development of efficient preventative and therapeutic strategies for a number of metabolic syndrome related diseases. Thus, healthy diet-derived factors are poised to play a key role in nutritional interventions for maintaining brain health and prevention of unhealthy diet effects on brain inflammation and malfunction.

The aryl hydrocarbon receptor (AHR) is a ligand-activated transcription factor that enables cells to adapt to changing conditions by sensing compounds from the diet, microbiome, and cellular metabolism 12. AHR ligands in circulation are reported to induce weight gain, glucose intolerance 13. The metabolites generated from IDO1 can promote the activity of the AHR signaling pathway 14.

MYC regulates systemic metabolism. Reduction of MYC in mice improves high-fat-diet-induced obesity, insulin resistance, hepatic steatosis and steatohepatitis^15^. Administration of the MYC inhibitor has beneficial effects on high-fat-diet-induced metabolic disorders 15. c-MYC overexpression results in an increase in reactive oxygen species (ROS) and DNA damage 16, which contributes to the progression of aging 17. The unifying aspect that translates from mice to humans is the diet regulated MYC signature. Dietary fat intake does not only amplify the MYC transcriptional program but can enrich for it 18, 19. MYC mediated-increase in reactive oxygen species (ROS) and DNA damage 16 could also induce the activation of the cGAS/STING inflammatory pathway 20, 21.

Altogether, these findings raise questions as to whether diet-derived factors have a direct impact on brain function; how healthy diet-derived factor(s) impact the nervous system functionally that may reverse adverse effects on the brain caused by an unhealthy diet are unknown. In this study, these issues are explored.

Recently, a growing interest in exosome-like nanoparticles (ELNs) from a variety of plants has emerged, especially those with medicinal benefits such as garlic. We 22 and other groups 23 have demonstrated that orally given ELNs inhibit the development of mouse colitis. Circulating nanoparticles such as exosomes can cross the BBB in both directions 24, 25. However, whether ELNs have a direct effect on the expression of c-Myc, AHR and IDO1 in the brain is not clear. In this study, as proof-of-concept, we show that oral administration of garlic ELNs (GaELNs) resolves the HFD-induced brain inflammation and obesity in a HFD induced mouse obesity model. GaELNs are preferentially taken up by microglial cells and inhibit brain inflammation via IDO1 mediated AHR pathway and c-Myc mediated c-GAS/STING inflammatory pathway. These findings provide cellular and molecular insight into how diet-derived factors such as ELNs modulate neuroimmune function and metabolic activity by ELN mediated targeting of microglial cells.

## Results

### Oral administration of GaELNs inhibits the production of an array of inflammatory cytokines and reverses HFD induced mouse obesity

**Figure 1:**
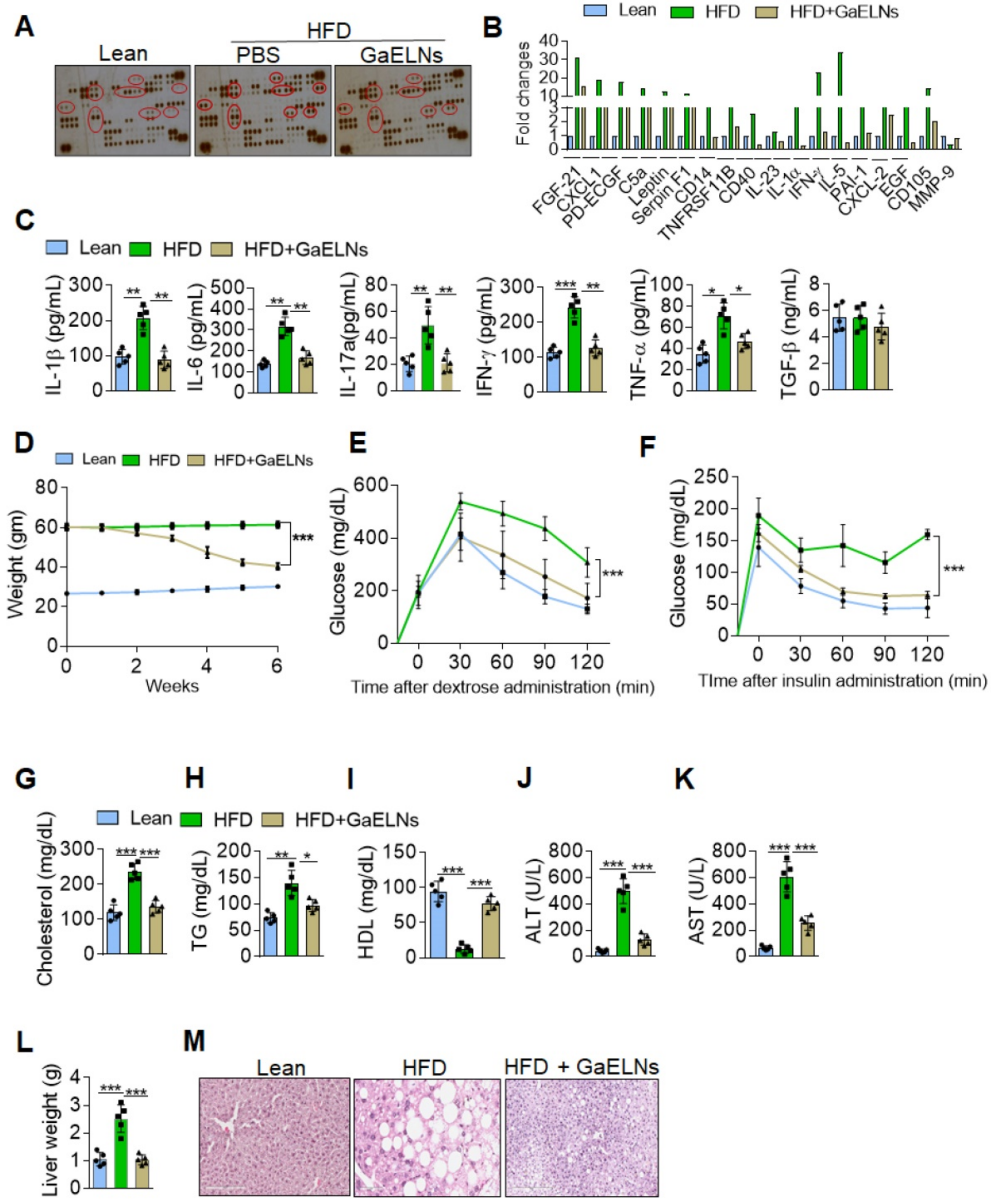
** GaELNs decreases inflammation and reverses HFD induced obesity in mice.** (A). Analysis of cytokine level in plasma from lean, HFD and GaELNs treated HFD mice for 6 weeks using mouse cytokine array. The difference in the spot intensity is circled. (B). Quantification of relative intensity of the selective upregulation and downregulation of cytokines. (C). Inflammatory cytokines IL-1β, IL-6, IL-17A, IFN-γ, and TNF-α in the plasma of lean, HFD and GaELNs treated mice were quantified by ELISA (n = 5). (D). Body weight of lean, HFD and GaELNs treated mice (n = 5/group). (E). Glucose tolerance test was determined in lean, HFD and GaELNs treated mice as described in the Materials and Methods (n = 5/group). (F). Insulin resistance testing was conducted on lean, HFD and GaELNs treated mice as described in the Materials and Methods (n = 5/group). (G-I). Lipid profile was determined in Lean, HFD and GaELNs treated mice plasmid (n = 5). (G) Cholesterol (H) Triglycerides. (I) HDL. (J). Liver damage was determined by liver specific enzymes in lean, HFD and GaELNs treated mice using plasma (n = 5/group). (J). Alanine aminotransferase (ALT). (K) Aspartate aminotransferase (AST). (L). Liver weight of HFD control and GaELNs treated mice (n = 5/group). (M). Histology of liver showing lipid deposits and cirrhosis. The values are expressed as mean ± SD for three independent assays. *p < 0.05, **p < 0.01, ***p < 0.001 compared with the untreated group using one-way ANOVA with Turkey's Multiple comparison test.

Chronic systemic low-grade inflammation is a hallmark of obesity, which plays a pathogenic role in development of diabetes, atherosclerosis, and cardiovascular diseases 26. But no effective therapeutic strategy has been developed for prevention or inhibition of chronic inflammation. In general, healthy diet-derived factors prevent chronic inflammation. Therefore, we sought to determine whether ELNs systemically inhibit the production of inflammatory cytokines in high-fat diet (HFD) fed mice. To address this issue, garlic ELNs (GaELNs) were used as proof-of-concept; we screened the cytokine levels in plasma of lean, HFD and GaELNs treated HFD mice. GaELNs were isolated, purified, and characterized (Figure S1 and Table S1). The cytokine array showed that HFD mice had significantly increased levels of an array of cytokines and chemokines compared to lean mice (mice fed a regular chow diet) (Figure 1A-B). These increased level of cytokines and chemokines were significantly decreased in GaELNs treated HFD mice. Because we were focusing on inflammatory cytokines that play a major role in developing obesity, we quantified the level of IL-1β, IL-6, IL-17A, IFN-γ and TNF-α in the plasma of lean, HFD and GaELNs treated HFD mice. The inflammatory cytokines IL-1β, IL-6, IL-17A, IFN-γ and TNF-α levels were significantly elevated in HFD mice compared to lean mice. In contrast, GaELNs treated HFD mice showed a decreased level of the inflammatory cytokines IL-1β, IL-6, IL-17A, IFN-γ and TNF-α (Figure 1C). These results suggest that GaELNs systemically inhibit the HFD induced array of inflammatory cytokines in HFD induced obesity in mice.

Obesity triggered inflammation can lead to insulin resistance, which in turn can lead to type 2 diabetes 27. Type 2 diabetes is the most common form of diabetes and accounts for approximately 90% of diabetes cases 28. To address the therapeutic role of GaELNs in type 2 diabetes, C57BL/6 mice were fed the high-fat diet for 12 months to develop an obesity and insulin resistance that closely mimics human obesity associated type 2 diabetes with insulin resistance 29. Before gavaging HFD mice with GaELNs, we measured glucose tolerance and insulin resistance to confirm that the mice were diabetic and had developed an insulin resistance (Figure S2A). Then, GaELNs (1×10^10^ particles) were gavage administrated daily to HFD mice for 6 weeks (Figure S2A) and glucose tolerance and insulin resistance assays were conducted every week. A significant decrease in body weight was observed after six weeks of GaELNs treatment (Figure 1D). There were no significant changes in food and water intake in these mice (Figure S2B). Oral glucose tolerance, evaluated by the OGTT test, was significantly improved in GaELNs treated mice compared to untreated HFD mice (Figure 1E). Further, insulin sensitivity was significantly increased in GaELNs treated mice compared to untreated HFD mice as shown by the insulin tolerance test (ITT) (Figure 1F). These results indicate that GaELNs treatment reverses glucose intolerance and improves insulin sensitivity in HFD mice. Furthermore, GaELNs treated HFD mice showed significantly lower levels of cholesterol, triglycerides and increased HDL levels compared to PBS treated HFD mice (Figure 1G-I). Also, GaELNs significantly decreased AST and ALT enzyme activity in HFD mice plasma compared to PBS treated HFD mice (Figure 1J-K). In addition, GaELNs significantly decreased liver weight and fat deposit in HFD mice compared to control HFD mice (Figure 1L-M). Collectively, these results suggest that GaELNs treatment reverses HFD-induced obesity associated insulin resistance.

### Oral administration of GaELNs is preferentially taken up by microglial cells and inhibition of IL-1β, TNF-α and IFN-γ expressed in the brain of HFD mice

**Figure 2:**
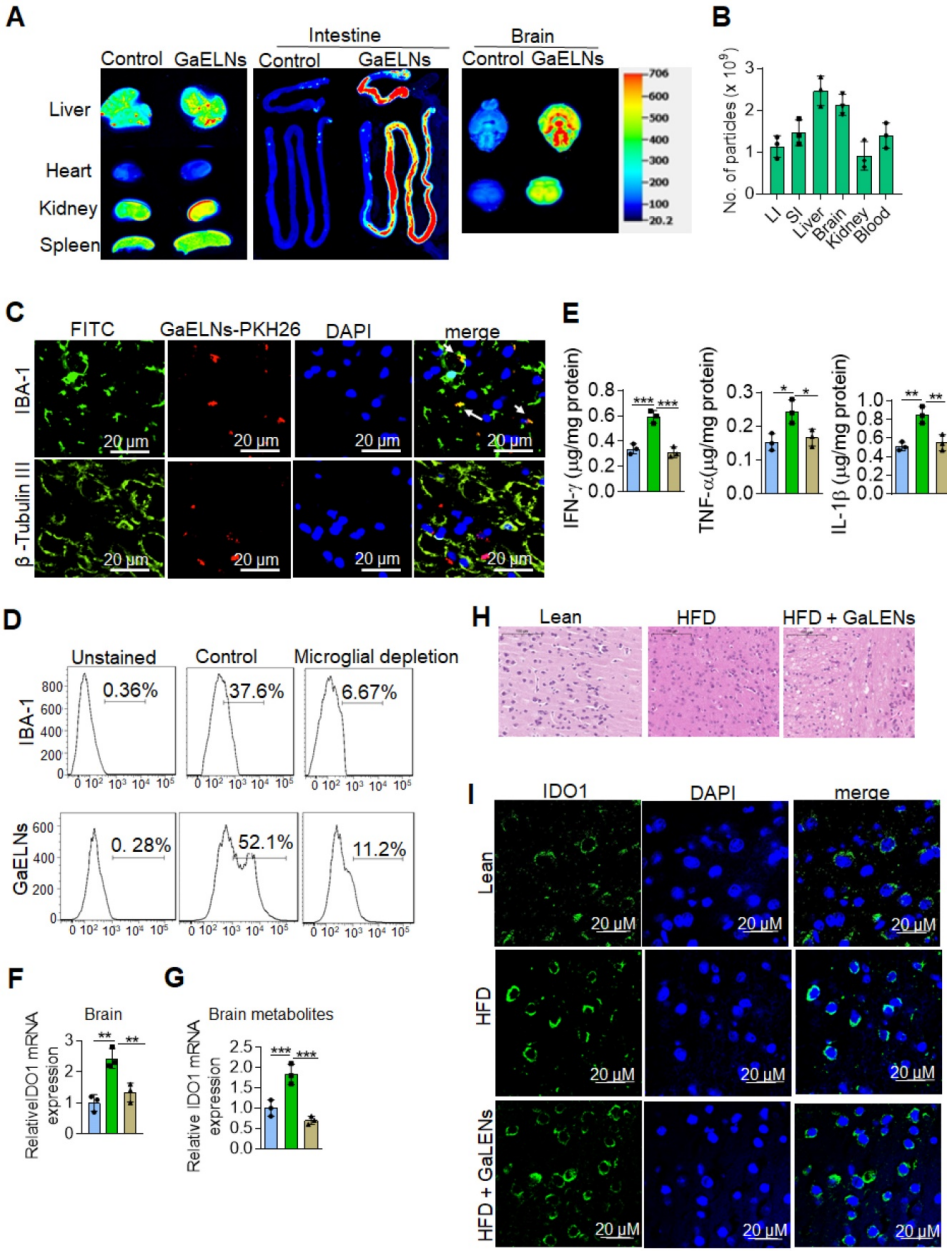
** GaELNs taken up by brain microglial cells and inhibit inflammatory cytokines and IDO1 expression.** (A). GaELNs were labelled with PKH26 and DiR dye. HFD fed mice were orally gavaged to for 24 h with labelled GaELNs (1×10^10^) and *in vivo* distribution of GaELNs in different organs was visualized by image analysis. (B). The number of particles distributed in each organ was determined based on fluorescent intensity of a standard known concentration of particles. (C). GaELNs uptake by brain microglial cells and neuronal cells was determined by confocal microscopy. Microglial cells and neuronal cells were stained using specific markers, IBA-1 and β-tubulin III, respectively. (D). Mice were calvaria injection with PBS and Clodrosomes (10 µL) for 72 h to deplete microglial cells and mice were gavage with PKH26 labelled GaELNs for 24 h. The GaELNs taken up by microglial cells were determined by flow cytometry. The microglial cells were stained with specific marker IBA-1 antibody. (E). The level of inflammatory cytokines INF-γ, TNF-α and IL-1β in brain of lean, HFD and GaELNs treated HFD fed mice was quantified by ELISA. (F). Total RNA isolated from lean, HFD and GaELNs treated HFD mice brain was subjected to real-time PCR for IDO1 mRNA expression. (G). BV2 cells were treated for 24 h with brain metabolites (100 µL/mL) derived from lean, HFD and GaELNs treated HFD fed mice. Total RNA isolated from these cells was subjected to real-time PCR for IDO1 mRNA expression. (H). Histology of brain sections show inflammatory state of the brain. (I). The brain section of lean, HFD and GaELNs treated HFD mice was stained with IDO1 antibody and visualized b confocal microscopy. The nucleus was stained with DAPI. The values are expressed as mean ± SD for three independent assays. *p < 0.05, **p < 0.01, ***p < 0.001 compared with the untreated group using one-way ANOVA with Turkey's Multiple comparison test.

*In vivo* targeted delivery of a therapeutic agent to the tissue where the pathogenic effect takes place is desirable for reducing side-effects, but it is challenging. We first sought to determine whether GaELNs preferentially traffic to brain. HFD fed mice were orally given GaELNs labelled with DiR dye and PKH26, or free DiR dye as a control. 24 h after gavage, mice were sacrificed. A relative high level of GaELNs traveled to brain, liver, small intestine, and large intestine compared with other organs (Figure 2A). The intensity of DiR dye labeled GaELNs in each organ was quantitatively analyzed. Approximately 2×10^9^ particles of the total GaELNs that were orally given traveled to the brain, 2.4×10^9^ particles traveled to the liver and 2x10^9^ particles remained in the intestine (Figure 2B). We then determined which type of brain cells take up the PKH26 labeled GaELNs. The mice were gavage-given fluorescent dye PKH26-labeled GaELNs. As Figure 2C shows, double positive PKH26^+^ and IBA-1^+^ cells were detected in brain tissue sections, indicating that GaELNs is selectively taken up by brain microglial cells whereas neuronal cells did not (Figure 2C). To further determine if microglial cells are the primary cells taking up GaELNs, brain macrophages were depleted with clodronate (CLO) or PBS as a control by parenchyma injection 30 before gavage administration of PKH26 labeled GaELNs. 72 h after the injection, the control and microglial depleted mice were orally gavage with PKH26 labelled GaELNs. 24 h after being gavage-given the labelled GaELNs, the PKH26 positive brain cells were quantified by flow cytometry. The microglial cells were stained with IBA-1 with FITC tagged secondary antibody. The results show that most of the IBA-1 positive microglial cells were successfully depleted, and depletion of microglial cells leads to decreasing GaELNs being detected in the brain (Figure 2D).

Microglial cells are one of the major immune cells releasing IL-1β, TNF-α and IFN-γ which plays a critical role in brain inflammation in high-fat diet induced obesity 31. Next, we examined whether GaELNs treatment would have effects on the brain, i.e., reduce expression of inflammatory cytokines including IL-1β, TNF-α and IFN-γ which induces expression of IDO1. Total brain tissue cell lysates were obtained from lean mice, HFD and GaELNs treated HFD mice and the level of inflammatory cytokines IFN-γ, TNF-α and IL-1β were measured. The results show that GaELNs treated HFD mice showed significant reduction of all three inflammatory cytokine levels in the brain compared to HFD fed mice (Figure 2E). Overproduction of IDO1 is involved in development of diabetes 32. IFN-γ induces the expression of IDO1 33. Our data show that the expression of IFN-γ is inhibited by GaELNs in HFD mice (Figure 1C). Corresponding with the decreased level of IFN-γ in the brain, the expression of IDO1 was decreased as well in the brain of GaELNs treated HFD mice (Figure 2F). The results generated in the *in vivo* study were confirmed by in vitro cell culture studies. BV2 mouse brain microglial cells were treated for 24 h with brain metabolites derived from lean, HFD and GaELNs treated HFD mice. Brain metabolites from GaELNs treated HFD mice significantly decreased IDO1 mRNA expression compared to HFD metabolite treated cells (Figure 2G). Further, the HE stained brain tissue section show that the infiltrated inflammatory cells are reduced in the GaELNs treated mice compared with HFD fed mice (Figure 2H). Also, the intensity signal of IDO1^+^ cells was much weaker in the GaELNs treated mice when compared with HFD fed mice (Figure 2H). Collectively, these results suggest that GaELNs inhibit the expression of IL-1β TNF-α, and IFN-γ in microglial cells and inhibition of IFN-γ leads to reduction of expression of IDO1 in HFD fed mice.

### GaELNs inhibit brain inflammation and neuron cell death in HFD mice

**Figure 3:**
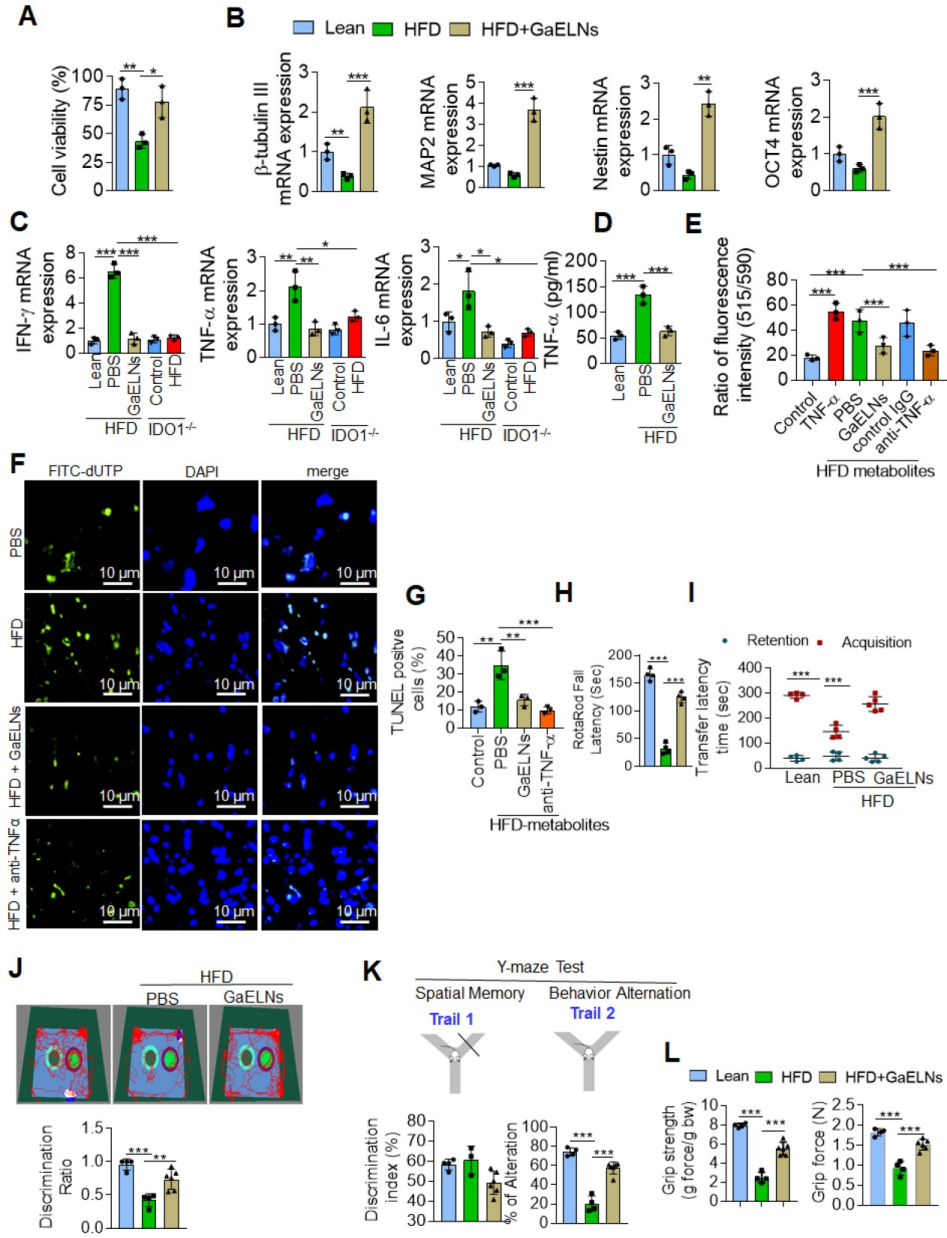
** GaELNs modulate brain metabolites and inhibit neuronal cell death in HFD fed mice**. (A). Isolated mouse primary neuronal cells were cultured in the presence of brain metabolites (100 µL/mL) derived from lean, HFD and GaELNs treated HFD fed mice. Cell viability was determined by the MTT assay as described in the Methods. (B). Total RNA isolated from mouse primary neuronal cells was cultured in the presence of brain metabolites (100 µL/mL) derived from lean, HFD and GaELNs treated HFD fed mice and subjected to real-time PCR for β-tubulin III, MAP2, nestin and OCT4 mRNA expression. The mRNA expression was normalized by actin mRNA expression (n = 3). (C). BV2 cells were treated with metabolites derived from lean, HFD fed mice, GaELNs treated HFD fed mice, IDO1^-/-^ control and HFD fed IDO1^-/-^ mice. Total RNA isolated from these cells was subjected to real-time PCR for IFN-γ, TNF-α and IL-6 mRNA expression. The mRNA expression was normalized to actin mRNA expression (n = 3). (D). BV2 cells were treated with brain metabolites (100 µL/mL) derived from lean, HFD fed mice, GaELNs treated HFD fed mice for 24 h. The level of TNF-α in culture supernatants was quantified by ELISA (n = 3). (E). Primary neuronal cells were treated with cultured supernatant (25%) from BV2 cells in the presence of anti-TNF-α neutralizing antibody and TNF-α (50 ng/mL) was used as positive control. The mitochondrial membrane potential was determined using the JC-10 fluorescent mitochondrial marker as described in the Methods. (F). BV2 cells were treated for 24 h with brain metabolites (100 µL/mL) derived from lean, HFD and GaELNs treated HFD fed mice. The conditioned media was collected from these cells and used to treat primary neuronal cells (25%) in the presence and absence of TNF-α neutralizing antibody for 24 h. *In vivo* cell death was determined by TUNEL staining as described in the Methods. (G). The number of TUNEL positive cells were quantified (n = 3). (H). Motor coordination of brain was assessed by Rotarod testing. The performance in the rotarod test of lean, HFD and GaELNs treated mice. (I). Transfer latency in elevated plus maze testing. Short-term memory of mice was assessed by a novel object recognition test (NORT) and Y-maze (spontaneous alteration and two-trail recognition test. (J). Examples of movement traces of lean, HFD and GaELNs treated HFD mice assessed by the NORT and values of the discrimination index ratio. (K). Summaries of Y-maze tests spontaneous alteration and two-trail recognition. (L). Cognitive function was assessed by grip strength in lean, HFD and GaELNs treated HFD mice. The values represent grip strength (n = 5/group). The values are expressed as mean ± SD for three independent assays. *p < 0.05, **p < 0.01, ***p < 0.001 compared with the untreated group using one-way ANOVA with Turkey's Multiple comparison test.

Microglia represent a specialized population of macrophage-like cells in the brain considered immune sentinels that are capable of an inflammatory response, are involved in phagocytosis of apoptotic cells in the developing brain and participate in control of neuronal excitability 34. Recent evidence suggest that microglia-neuronal communication plays a major role in diverse physiological and pathological processes in the brain 35. The increased level of inflammatory cytokines in the microglial cells, in particularly TNF-α, act on neuronal cells which induces neuronal cell apoptosis 36. It has been shown that a HFD induces depression-like behavior and altered neuronal activity in mice 37. Studies have shown that alteration of microglial cell function is responsible for pathological neuronal activity 38. However, our data show that neuronal cells did not take up GaELNs (Figure 2C). Next, we determined whether GaELNs have effects on neuronal activity by focusing on neuron viability via communication with microglial cells. We isolated E17 embryonic primary mouse neuronal cells. The isolated primary neuronal cells were cultured with brain metabolites (Listed in Table S2) derived from lean mice, HFD and GaELNs treated HFD mice. Cell viability was determined using the MTT assay. Cell viability was decreased in HFD brain metabolite treated primary neuronal cells compared to lean mice derived brain metabolite treated cells. Neuronal cells treated with brain metabolites from GaELNs treated HFD fed mice had a significant increase in cell viability compared to HFD brain-derived metabolites (Figure 3A). Further, we determined the effect of brain metabolites on microglial cell viability by MTT assay. There was no significant cell death was observed regardless the treatments applied (Figure S3A). It has been showed that inflammation have an effect on neuron differentiation 39, 40. Next, we sought to determine the neuronal differentiation in these cells treated with brain metabolites from GaELNs or PBS treated HFD mice. Neuronal differentiation was determined by quantification of neuronal differentiation marker gene expression such as β-tubulin III, MAP2, nestin and OCT4 mRNA expression. With the exception of β-tubulin III, HFD metabolites did not affect the expression of the other three analyzed genes. However, brain metabolites from GaELNs treated HFD mice significantly increased the expression of all four genes analyzed (Figure 3B) compared with lean mouse derived brain metabolites.

Microglial cell derived TNF-α induces neuronal cell death 36, and overproduction of IDO1 is involved in development of diabetes with a neurological effect via IDO1 regulated metabolites 32. Therefore, we wanted to know whether IDO1 mediated metabolites activate microglial cells which in turn are induced to produce a number of inflammatory cytokines including TNF-α that initiate neuronal cell death in HFD fed mice. BV2 cells were treated with brain metabolites derived from lean, HFD, GaELNs treated HFD mice, IDO1^-/-^ control and HFD fed IDO1^-/-^ mice. Total mRNA isolated from BV2 cells was subjected to real-time PCR for quantifying inflammatory cytokine expression. Brain metabolites derived from HFD mice significantly induced mRNA expression of IFN-γ, TNF-α and IL-6. In contrast, metabolites derived from GaELNs treated HFD mice showed decreased levels of expression of these inflammatory cytokines. However, the metabolites (Listed in Table S3) derived from HFD treated IDO1^-/-^ mice showed no induction of IFN-γ, TNF-α and IL-6 expression (Figure 3C). The real time PCR results were confirmed by ELISA analysis of the protein level of these inflammatory cytokines in the cultured supernatant of microglial cells treated with brain metabolites (Figure S3B). Collectively, these results suggest that brain metabolites derived from HFD mice induces IFN-γ, TNF-α and IL-β and HFD mediated induction of these cytokines is dependent on IDO1. It has been shown that neuron cell death is associated with memory loss and function 41. Among the inflammatory cytokines induced by HFD feeding that could potentially induce neuron cell death, TNF-α released from activated microglial cells was the most likely candidate. Therefore, we quantified the level of TNF-α in the media from BV2 cells treated with metabolites derived from the brains of lean, HFD and GaELNs treated HFD mice. The level of TNF-α in the cultured supernatants was significantly increased when the cells were treated with HFD brain metabolites compared to the metabolites from lean and GaELNs treated HFD mice (Figure 3D).

TNF-α induces apoptosis through increased ROS generation in mitochondria that contributes to neuronal cell death 42 . We then screened the GaELNs modulated ROS gene expression in the brain of HFD mice. Total RNA from brains of lean, HFD and GaELNs treated HFD mice was isolated and subjected to a qPCR array for the presence of the ROS gene family. We observed that GaELNs modulates the expression of several of these genes Figure S4A-B). We further confirmed qPCR array results by real-time PCR (Figure S4C-D). Then, we tested whether the metabolites released from microglial cells contribute to alter neuron cell mitochondrial membrane permeability which causes neuron cell death. Primary mouse neuronal cells were treated with culture media (25%) from BV2 cells treated for 24 h with the brain metabolites from lean, HFD, GaELNs treated HFD mice in the presence of anti-TNF-α antibody or a control IgG antibody and TNF-α (50 ng/mL) treated group served as a positive control. The mitochondrial membrane potential was determined using the JC-10 fluorescent mitochondrial membrane potential assay kit as per the manufacturer's protocol. The mitochondrial membrane potential was significantly increased in neuronal cells treated with culture media from BV2 cells treated with HFD mouse brain metabolites. However, the mitochondrial membrane potential was significantly decreased in neuronal cells treated with culture media derived from lean and GaELNs treated HFD mice. Addition of anti-TNF-α antibody to cultures prevented the HFD brain metabolite mediated increase in mitochondrial membrane potential (Figure 3E). Taken together, these results suggest that brain metabolites derived from HFD mice induce expression of TNF-α in the microglial cells which acts on neuronal cells and increases mitochondrial membrane potential. The increased level in mitochondrial membrane potential leads to apoptosis in the neuronal cells 43. Next, we quantified the level of apoptosis in the neuronal cells. We performed TUNEL staining to detect apoptotic cells by culturing primary neuronal cells for 24 h treated with condition media harvested from treated BV2 cells. The treatments include brain metabolites derived from lean, HFD and GaELNs treated HFD fed mice for 24 h. The number of apoptotic cells was significantly increased in brain metabolites derived from HFD mice compared to lean mice. Further, apoptotic cell death was significantly decreased in GaELNs and anti-TNF-α neutralizing antibody treated cells (Figures. 3F-G).

Several clinical studies have reported that obesity and type 2 diabetes have negative impacts on cognitive memory function, which is an issue of increasing clinical interest 44. We next sought to determine the functional role of GaELNs in improving memory function in HFD mice. To assess the memory function in these mice, we did several memory assessments tests such as the motor coordination/motor activity test, short-term memory by novel object recognition test (NORT), long-term memory by passive avoidance test (PAT), Y-maze test and neuro-muscular function by the grip test. From these tests we observed that HFD mice showed significantly decreased memory function. However, treatment of HFD mice with GaELNs significantly improved memory function (Figure 3 H-L).

In conclusion, HFD feeding induces an array of inflammatory cytokines including TNF-α and IFN-γ which in turn promotes neuron cell death and brain inflammation. GaELNs treatment via targeting microglial cells leads to inhibition of the expression of an array of inflammatory cytokines including IFN-γ and TNF-α. Therefore, brain inflammation and neuron cell death are inhibited in HFD fed mice.

### GaELNs reverse a high-fat diet induced insulin resistance through regulating the IDO1-AHR signaling pathway

**Figure 4:**
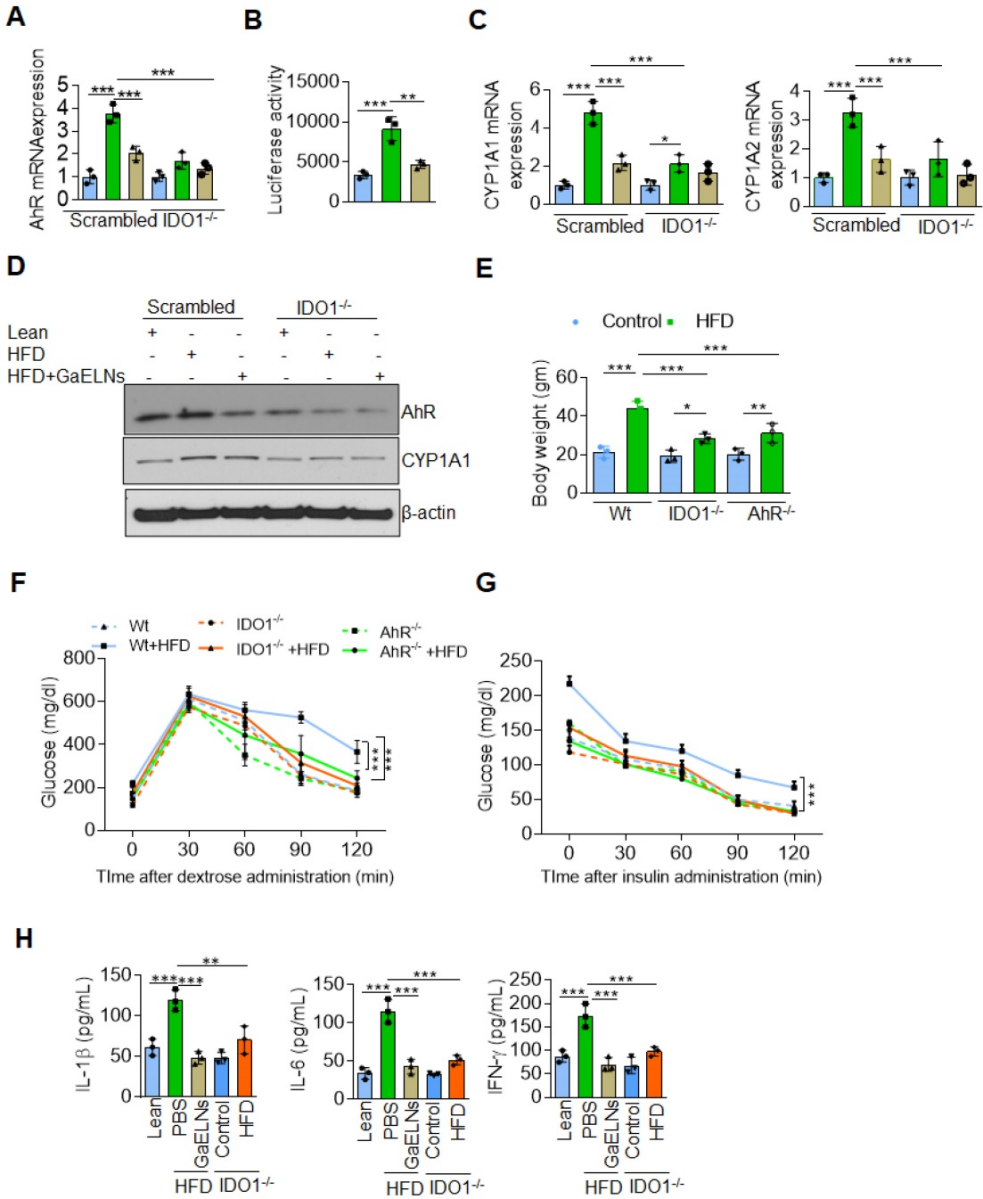
** GaELNs reverse insulin resistance in HFD mice by inhibiting activation of IDO1-AHR pathway**. (A). BV2 cells were transfected with scrambled and CRISPR/cas9 IDO1 plasmids and cells were treated for 24 h with brain metabolites (100 µL/mL) from lean, HFD and GaELNs treated HFD fed mice. Total RNA isolated from these cells was subjected to real-time PCR for AHR mRNA expression. The mRNA expression was normalized by actin mRNA expression (n = 3). (B). HEPA1.1 cells were transfected with constituently activated AHR-Luc promoter and cells were treated with brain metabolites (100 µL/mL) from lean, HFD and GaELNs treated HFD fed mice for 24 h. AHR promoter activity was measured by luciferase activity. (C). Scrambled and IDO1 knockout BV2 cells were treated for 24 h with brain metabolites (100 µL/mL) from lean, HFD and GaELNs treated HFD fed mice. Total RNA isolated from these cells was subjected to real-time PCR for CYP1A1 and CYP1A2 mRNA expression. The mRNA expression was normalized by actin mRNA expression (n = 3). (D). Total cell lysates obtained from these cells were subjected to western blot analysis for AHR, and CYP1A1 expression. (E). Body weight of WT, AHR^-/-^ and IDO1^-/-^ mice was determined (n = 5/group). (F). Glucose tolerance test was determined in WT, AHR^-/-^ and IDO1^-/-^ mice receiving the normal diet and HFD fed mice (n = 5/group) as described in the Materials and Methods. (G). The insulin resistance assay was conducted on WT, AHR^-/-^ and IDO1^-/-^ mice receiving the normal diet and HFD fed mice (n = 5/group) as described in the Methods. (H). The level of inflammatory cytokines IL-1β, IL-6 and IFN-γ was determined in plasma of lean, HFD, IDO1^-/-^ control and HFD fed mice (n = 5 mice/group) by ELISA. The values are expressed as mean ± SD for three independent assays. **p < 0.01, ***P < 0.001 compared with the untreated group using one-way ANOVA with Turkey's Multiple comparison test.

IDO1 is the rate-limiting enzyme in the tryptophan catabolism within the kynurenine (KYN) pathway. The over expression of IDO1 may leads to tryptophan depletion and increased accumulation of kynurenine which can bind the aryl hydrocarbon receptor (AHR) 45. Epidemiologically, exposure to AHR ligands contributes to obesity and type 2 diabetes (T2D) 46. We have recently shown that HFD feeding increases the expression of AHR which contributes to promoting insulin resistance in mice 29. Therefore, we next investigated whether brain metabolites of HFD mice treated with GaELNs could modulate IDO1-AHR pathway and reverse the insulin resistance in the HFD fed mice. To determine whether GaELNs inhibit the expression of IDO1 mediated AHR that could contribute to insulin sensitivity in HFD mice. Brain microglial cells were transfected with CRISPR-cas9 scrambled and IDO1 plasmid to knockdown IDO1 expression. The cells were then treated with brain metabolites derived from lean, HFD and GaELNs treated HFD mice for 24 h. Total RNA isolated from these cells was subjected to real-time PCR for AHR mRNA expression. HFD derived brain metabolites were significantly increased AHR mRNA expression compared to lean mice. Further, GaELNs treated HFD brain metabolites significantly decreased AHR mRNA expression in wild-type microglial cells. However, there was no significant changes in AHR mRNA expression in IDO1^-/-^ knockout derived microglial cells regardless which treatment was given (Figure 4A). We next determined whether brain metabolites can regulate AHR promoter activity. Thus, HEPA1.1 cells were transfected with constitutively activated AHR promoter and cells were treated with brain metabolites derived from lean, HFD and GaELNs treated HFD mice. AHR promoter activity was significantly increased in HFD metabolites treated cells compared to lean metabolites treated cells. In contrast, GaELNs treated HFD brain metabolites significantly decreased AHR promoter activity in HEPA1.1 cells (Figure 4B). Further, the role of IDO1 in AHR activation in the HFD mice was confirmed by quantifying the mRNA expression of CYP1A1 and CYP1A2 which are both known to be activated by AHR signaling 47. CYP1A1 and CYP1A2 mRNA was significantly increased in scrambled CRISPR/cas9 transfected microglial cells treated with HFD mouse brain metabolites, and GaELNs inhibited the induction whereas knockout of IDO1 cancelled the GaELNs mediated inhibitory effects (Figure 4C), suggesting that IDO1 is located upstream of AHR for induction of CYP1A1 and CYP1A2. We further confirmed that real-time PCR results of AHR and CYP1A1 expression by western blot analysis (Figure 4D). The role of IDO1 and AHR in developing obesity was further indicated by the facts that IDO1 and AHR knockout mice showed a significant decrease in body weight compared to WT control mice (Figure 4E). The blood glucose tolerance test was significantly decreased in IDO1^-/-^ and AHR^-/-^ knockout mice compared to littermate C57BL/6 mice fed the HFD (Figure 4F), and insulin sensitivity was significantly increased in IDO1^-/-^ and AHR^-/-^ knockout mice compared to littermate mice (Figure 4G). In addition, the level of the inflammatory cytokines IL-1β, IL-6 and IFN-γ were significantly decreased in HFD IDO1^-/-^ knockout mice compared to HFD WT littermate mice (Figure 4H). Collectively these results suggest that GaELNs inhibit induction of brain inflammatory cytokines and reverses the high-fat diet induced obesity through regulating the IDO1-AHR axis signaling pathway.

### GaELN PA (36:4) binding to microglial cell BASP1 is required for uptake of GaELNs and subsequent inhibition of IDO1 expression

**Figure 5:**
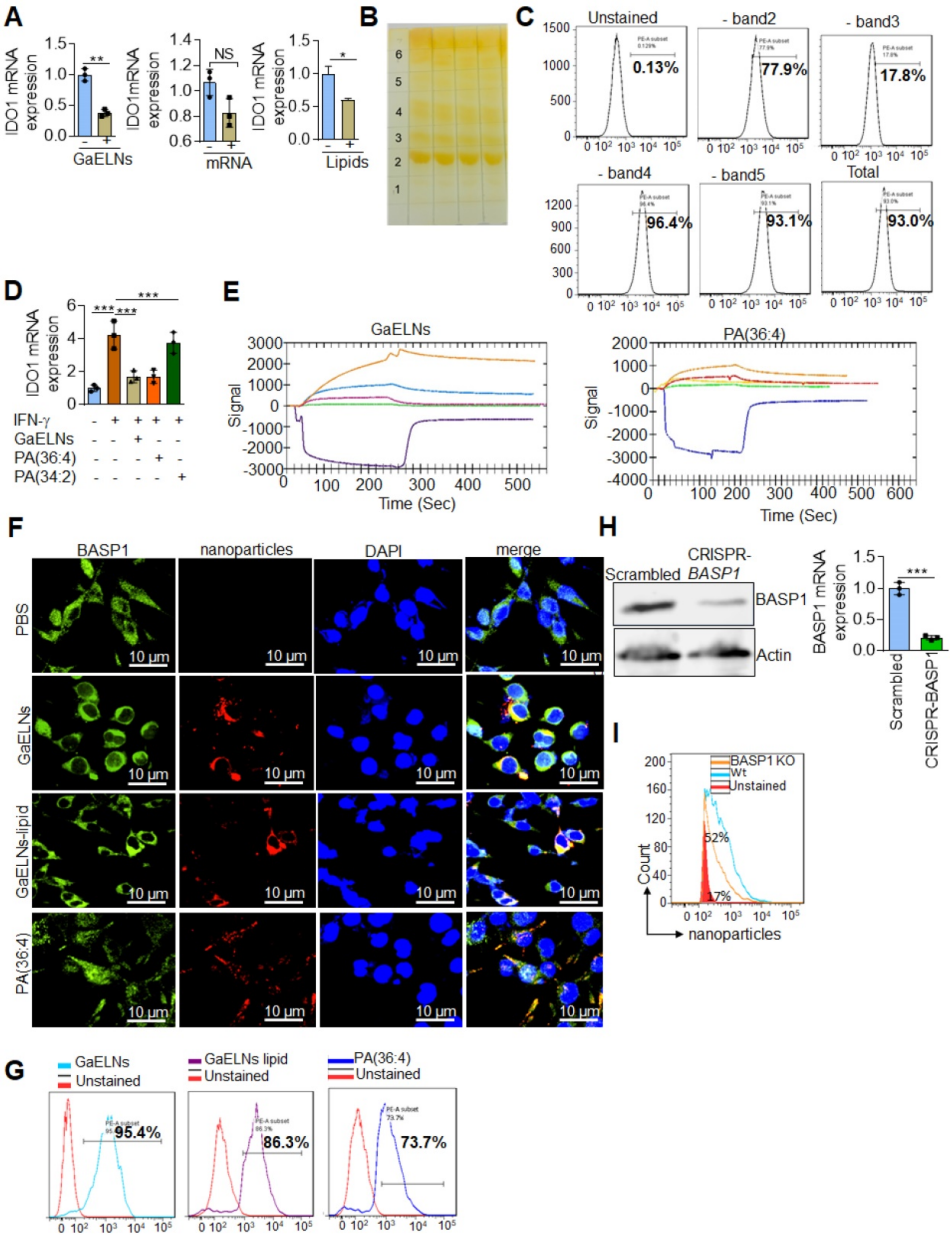
** GaELNs contain PA (36:4) which regulates IFN-γ induced IDO1 expression in brain microglial cells**. (A). BV2 cells were treated for 24 h with GaELNs (1×10^8^ nanoparticles/mL), lipid nanovector packaged GaELNs total RNA and GaELNs derived lipids (1 µg/mL). Total RNA isolated from these cells was subjected to real-time PCR for IDO1 mRNA expression (n = 3). (B). Total lipids from GaELNs were extracted and separated by TLC and stained using iodine vapor as described in the Methods. (C). Lipid nanoparticles were made from lipids extracted from TLC plates by removing each band and pooling together the remaining lipids. The lipid nanoparticles were labelled with PKH26 and incubated with BV2 cells for 24 h. The uptake of lipid nanoparticles was determined by flow cytometry. (D). BV2 cells were stimulated with IFN-γ in the presence or absence of GaELNs total lipids, PA (36:4) and PA (34:2) (1 µg/mL). Total RNA isolated from these cells was subjected to real-time PCR for IDO1 mRNA expression. The mRNA expression was normalized by actin mRNA expression (n = 3). (E). Identification of GaELNs lipids and PA (36:4) binding protein in the brain of HFD mice. Lipid nanoparticles were prepared from GaELNs total lipids and PA (36:4) was immobilized on a LIP-1 sensor. Total cell lysates of HFD fed mice brains were used as analyte as described in the Methods. The lipid-protein interaction was determined using a SPR sensogram. Protein bound with lipid nanoparticles was eluted by NaOH (200 mM) and the fraction was collected for MS/MS analysis for protein identification. The lipid-binding proteins are listed in Table S6 and S7. (F). BV2 cells were treated for 24 h with PKH26 labelled lipid nanoparticles from GaELNs total lipids and PA (36:4). Cells were fixed and stained with anti-BASP1 antibody and lipid nanoparticles binding to BASP1 were visualized by confocal microscopy. The nucleus was stained with DAPI. (G). Lipid nanoparticles were prepared from GaELNs total lipids and PA (36:4) and labelled with PKH26. The labelled particles (1 µg/mL) were incubated at 4°C overnight with total cell lysates derived from BV2 cells. Then, anti-BASP1 antibody was added and the was mixture rotated for 3 h at 4 °C. The antibody-nanoparticle complexes were pulled-down using protein-G agarose beads. Lipid nanoparticles were eluted from this complex by elution buffer (100 mM Glycine). The eluted nanoparticles were quantified using flow cytometry. (H). Knockdown of BASP-1 in BV2 cells was confirmed by BASP1 expression by western blot and real-time PCR. (I). BV2 WT and BASP1 KO cells were treated with PKH26 labelled GaELNs and uptake of particles by these cells was quantified by flow cytometry. The values are expressed as mean ± SD for three independent assays. **p < 0.01, ***P < 0.001 compared with the untreated group using one-way ANOVA with Turkey's Multiple comparison test.

We have previously shown that edible plant exosomes consist of proteins, lipids, and RNAs 48. Next, we extended our finding to determine whether RNA or lipids derived from GaELNs have a potential role in inhibiting IDO1 expression. We extracted total RNA and lipids from purified GaELNs, then total GaELNs RNA was encapsulated in lipid nanovectors made with lemon ELNs 49, 50 derived lipids as described in methods. Brain microglial BV2 cells were treated for 24 h with total RNA and total lipids (1 µg/mL) derived from GaELNs. Total RNA isolated from these cells was subjected to real-time PCR for IDO1 mRNA expression. Lipids derived from GaELNs potentially inhibit IDO1 mRNA expression in these cells. However, there was no significant change in IDO1 mRNA expression in GaELNs RNA treated cells (Figure 5A). This result suggests that lipids from GaELNs have the potential effect to modulate IDO1 expression in microglial cells. Next, we determined which GaELNs-derived lipids play a role in the inhibition of IDO1 expression. Total lipids from GaELNs were isolated and separated by thin-layer chromatography (TLC) (Figure 5B). Lipids from each band were excised and pooled together with the remaining lipids; then nanoparticles were made from the prepared lipids using a protocol previously described 51. The lipid nanoparticles were labelled with PKH26 and incubated with BV2 cells for 24 h. The cells were washed with PBS and lipid nanoparticles taken up by brain microglial cells were determined by flow cytometry. Lipid nanoparticles made with band 3 removed led to a significant decrease in uptake by microglial cells (Figure 5C). This result indicates that lipids from band 3 play an important role in GaELNs being taken up by microglial cells. Further, we extracted band 3 lipid and total lipids from GaELNs and subjected to mass spectrophotometry. The lipid profile of total lipids from GaELNs and band 3 are listed in Table S4 and S5. Identified in band 3 was phosphatidic acid (PA) and the band was enriched with PA (36:4) and PA (34:2). Phosphatidic acid (PA) participates in and regulates numerous cellular pathways, including ligand-mediated secretion and endocytosis 52. Next, we sought to determine which PA lipids from the GaELNs plays an important role in regulation of IDO1 expression. BV2 brain microglia cells were treated for 24 h with IFN-γ in the presence or absence of GaELNs, PA (36:4), or PA (34:2). Total RNA isolated from these cells was subjected to real-time PCR for IDO1 expression (Figure 5D). PA (36:4) significantly inhibited IFN-γ induced IDO1 expression compared to PA (34:2). The level of inhibition of IDO1 expression by PA (36:4) was comparable with inhibition obtained with GaELNs. Next, we explored the molecular mechanism underlying PA (36:4) binding to protein in the brain that modulates IDO1 expression. We utilized the SPR technique to identify PA (36:4) binding protein. We made lipid nanoparticles from GaELNs total lipids and PA (36:4) and immobilized each on a LIP-1 sensor chip. Total cell lysates derived from HFD mouse brains were used as an analyte that was run over the sensor chip. After allowing protein binding with lipid nanoparticles, the sensor chip was thoroughly washed to remove the unbound protein. The GaELNs total lipids and PA (36:4) binding protein was eluted from the chip using NaOH (200 mM) (Figure 5E). The eluted protein was subjected to MS/MS analysis for further identification of protein. PA (36:4) binding protein is listed in the Table S6 and S7. We identified that PA (36:4) and GaELNs lipids were binding with brain acid soluble protein 1 (BASP1). We further confirmed by confocal microscopy and a pull-down assay that GaELNs and PA (36:4) were interacting with BASP1. GaELNs, GaELNs-derived lipid nanoparticles and PA (36:4) lipid nanoparticles were labelled with PKH26. BV2 cells were treated for 24 h with the labelled particles and the cells were fixed and stained with anti-BASP1 antibody. The interacting of these nanoparticles with BASP1 was visualized using confocal microscopy (Figure 5F). We confirm by flow cytometry that GaELNs, total lipids derived from GaELNs and PA (36:4) were interacting with BASP1 in BV2 cells (Figure 5G). We next sought to determine the molecular mechanism underlying GaELNs interacting with BASP1 in the brain of HFD mice. To test the role of BASP1, we knocked out the BASP1 in BV2 cells using a CRISPR-BASP1 lentiviral plasmid. The knockdown of BASP1 in BV2 cells was confirmed by western blot and real-time PCR analysis (Figure 5H). BASP1 knockdown BV2 cells were treated with PKH26 labelled GaELNs, GaELNs lipid and PA (36:4). The uptake of these nanoparticles by BV2 cells was quantified by flow cytometry. Interestingly, the uptake of these nanoparticles in BASP1 KO BV2 cells was significantly decreased (Figure 5I). This result indicates that GaELN PA (36:4) binding to BASP1 in BV2 cells is required for uptake of GaELNs.

### GaELN PA is required for BASP1 mediated inhibition of inflammatory cytokines induced by the c-Myc regulated cGAS/STING pathway

**Figure 6:**
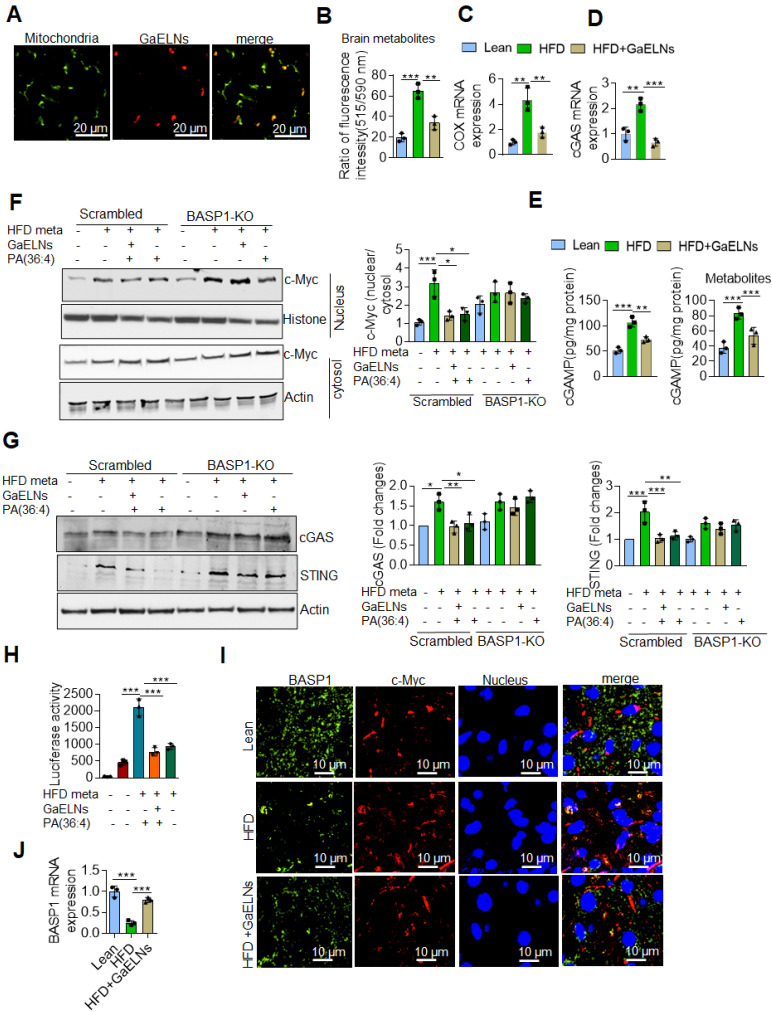
** GaELNs modulate cGAS-STING pathway mediated BASP1/c-MYC interaction and regulates inflammatory cytokine expression in microglial cells.** (A). GaELNs (1 ×10^10^ nanoparticles/mL) were labeled with PKH26 and orally given to mice over a 4 h period. The GaELNs targeting mitochondria in the brain were visualized using confocal microscopy. Mitochondria were stained with FITC labelled mitochondrial marker (B). BV2 cells were treated for 24 h with brain metabolites (100 µL/mL) derived from lean, HFD and GaELNs treated HFD fed mice. The mitochondrial membrane potential was determined using the JC-10 fluorescent mitochondrial membrane potential assay kit marker as described in the Methods. (C). BV2 cells were treated with brain metabolites (100 µL/mL) derived from lean, HFD and GaELNs treated HFD fed mice for 24 h. Total DNA was isolated from total cells and the cytosolic fraction. The amount of mtDNA in the cytosol was quantified using a specific primer for cytochrome c oxidase. The DNA level was normalized to total cytochrome c oxidase (n = 3). (D). Total RNA isolated from lean, HFD and GaELNs treated mice brains and subjected to quantitative real-time PCR for cGAS mRNA expression. The mRNA expression was normalized to actin mRNA expression (n = 3). (E). BV2 cells were treated with brain metabolites (100 µL/mL) derived from lean, HFD and GaELNs treated HFD fed mice for 24 h. The level of cGAMP was quantified in total cell lysates obtained from these cells using ELISA (n = 3). (F). Scrambled and BASP1-knockout BV2 cells were stimulated with HFD metabolites (HFD-meta) (100 µL/mL) and treated for 24 h with GaELNs (1×10^8^ nanoparticles/mL) and PA (36:4) (1 µg/mL). Total cell lysates obtained from these cells were subjected to nuclear and cytoplasmic extraction. The cytoplasmic and nuclear level of c-Myc was determined by western blot analysis. Protein level was normalized by the level of actin and histone in the cytoplasm and nucleus, respectively. The ratio of the level of c-Myc in nucleus to cytosol was calculated. (G). Scrambled and BASP-1 knockout BV2 cells were treated with metabolites (100 µL/mL) derived from HFD brain (HFD-meta) and cells were treated for 24 h with GaELNs (1×10^8^ nanoparticles/mL) and PA (36:4) (1 µg/mL). Total cell lysates obtained from these cells were subjected to western blot analysis for cGAS and STING expression. The band intensity was determined by ImageJ software. (H). BV2 cells were transfected with PGL3-c-Myc-Luc promoter and the cells were treated with HFD metabolites (100 µL/mL) with or without GaELNs (1×10^8^ nanoparticles/mL) and PA (36:4) (1 µg/mL) for 48 h. The promoter activity was measured by luciferase activity in these cells. (I). Brain sections from lean, HFD and GaELNs treated HFD fed mice were stained with BASP1 and c-Myc. The localization of BASP1 and c-Myc was visualized by confocal microscopy and the nucleus was stained with DAPI. (J). Total RNA isolated from lean, HFD and GaELNs treated mice brain and subjected to quantitative real-time PCR for BASP1 mRNA expression. The mRNA expression was normalized to actin mRNA expression (n = 3). The values are expressed as mean ± SD for three independent assays. **p < 0.01, ***P < 0.001 compared with the untreated group using one-way ANOVA with Turkey's Multiple comparison test.

BASP1 is involved in cytoskeletal and lipid raft dynamics, as well as in the nuclear regulation of transcription 53. However, a role in inhibition of production of inflammatory cytokines has not been previously reported. Our results show that GaELNs travel to the mitochondria (Figure 6A). Releasing mitochondria DNA could activate the cGAS/STING mediated inflammatory pathway 54. Given this data we began testing whether metabolites derived from HFD mice altered the mitochondrial membrane potential, and subsequently released the mtDNA into the cytosol. BV2 cells were treated with brain metabolites derived from lean, HFD and GaELNs treated HFD mice and the mitochondrial membrane potential was determined in these cells. The mitochondrial membrane potential was increased in HFD metabolite treated BV2 cells. In contrast, the mitochondrial membrane potential was significantly decreased in BV2 cells treated with metabolites derived from GaELNs treated HFD mice (Figure 6B). Next, we quantified the level of mtDNA in the cytosol by measuring the cytochrome c oxidase level. The level of mtDNA content in the cytosol was significantly increased in HFD metabolite treated cells compared to lean metabolite treated cells and decreased in GaELN treated cells (Figure 6C). Releasing mtDNA content into the cytosol is known to activate cGAS and promote induction of cGAMP that serves as a ligand to activate STING which in turn leads to inducing an array of inflammatory cytokines 55. The HFD mediated activation of cGAS was evident by the fact that the level of cGAMP which is catalytically synthetized by cGAS was significantly increased in HFD mouse brains and brain metabolite treated BV2 cells (Figure 6D-E).

Next, the linkage between BASP1 mediated inhibition of the activity of c-Myc and c-Myc driven cGAS/STING inflammatory pathway was investigated. Recent studies have shown that activation of BASP1 inhibits c-Myc mediated macrophage inflammation via activation of the cGAS-STING pathway 56. The STING promoter sequencing has a c-Myc binding site; c-Myc induces STING expression and overexpression of c-Myc causes brain inflammation and accelerates aging 21, 57. Therefore, we sought to determine whether BASP1/PA interaction plays a key inhibitory role in the cGAS-STING pathway mediated induction of inflammatory cytokines through c-Myc transcription factor. BV2-WT and BASP1 KO microglial cells were treated with brain metabolites derived from HFD mice (HFD meta) in the present or absent PA (36:4), a ligand binding BASP1, and GaELNs which contained PA was used as a positive control. Nuclear and cytosolic fractions isolated from these cells were subjected to western blot analysis for c-Myc protein expression. The level of c-Myc was significantly increased in the nuclear fraction of HFD metabolites treated BV2 cells and significant decreased in GaELNs and PA (36:4) treated cells. Notably, the level of c-Myc was significantly increased in BASP1 KO BV2 cells (Figure 6F). We confirmed these results via confocal localization of c-Myc in these cells (Figure S5). Similarly, cytosolic levels of cGAS and STING were significantly increased in HFD metabolite treated cells and decreased levels of cGAS and STING were observed in GaELNs and PA (36:4) treated cells. In addition, an increased level of cGAS and STING expression was observed in BASP1 KO BV2 cells (Figure 6G). We then transfected BV2 cells with c-Myc promoter driven luciferase gene (pGL3-basic-c-Myc promoter obtained from Addgene) and treated the cells with brain metabolites derived from lean, HFD and mice treated with GaELNs and PA (36:4). c-Myc promoter activity was significantly increased in HFD metabolite treated cells compared to lean metabolite treated cells (Figure 6H). In addition, the c-Myc promoter activity was significantly decreased in GaELN and PA (36:4) treated cells. Collectively, these results suggest that brain metabolites from HFD mice induces c-Myc expression as well as activation of c-Myc; c-Myc then promotes the expression of STING. The interaction of GaELN PA (36:4)/BASP1 leads to inhibiting c-GAS/STING mediated expression of inflammatory cytokines via inhibition of the c-Myc activation in brain microglial cells.

**Figure 7:**
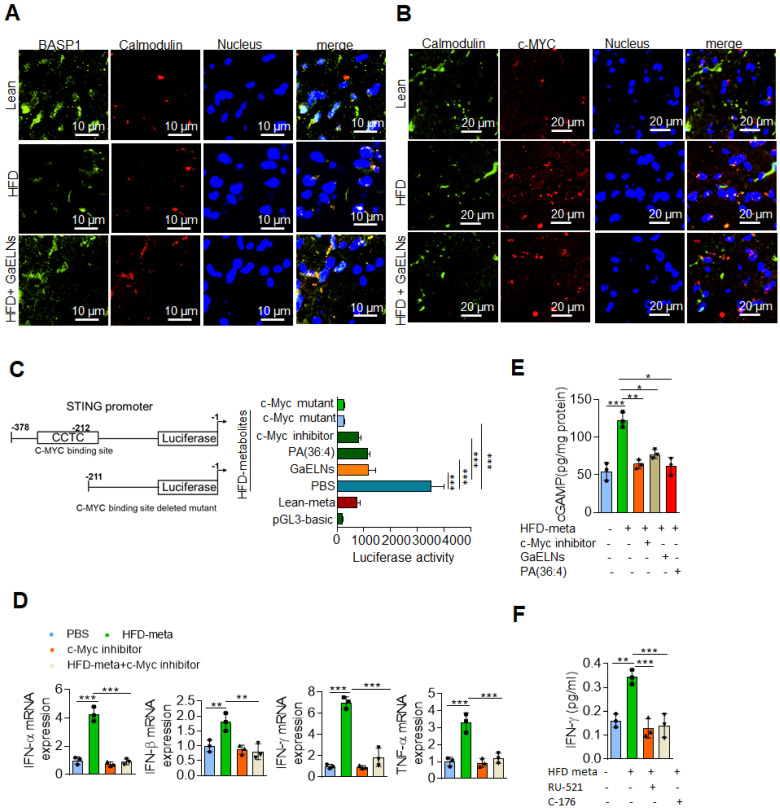
** GaELNs modulates BASP1 and calmodulin interaction which regulates STING-cGAS pathway.** (A). Brain sections from lean, HFD and GaELNs treated HFD fed mice were stained with BASP1 and calmodulin. The localization of BASP1 and calmodulin was visualized by confocal microscopy and the nucleus was stained with DAPI. (B). Brain sections from lean, HFD and GaELNs treated HFD fed mice were stained with c-Myc and calmodulin. The localization of c-Myc and calmodulin was visualized by confocal microscopy and the nucleus was stained with DAPI. (C). BV2 cells were transfected with the pGL3-STING-Luc promoter and c-Myc binding site deleted STING-Luc promoter. Cells were for 24 h with HFD metabolites (100 µL/mL) and with or without GaELNs (1×10^8^ nanoparticles/mL), PA (36:4) (1 µg/mL) and c-Myc inhibitor (10 µM). The promoter activity was measured by luciferase activity in these cells (n = 3). (D). BV2 cells were treated with HFD metabolites (100 µL/mL) and with or without c-Myc inhibitor (10 µM) for 24 h. Total RNA isolated from these cells was subjected to real-time PCR for IFN-α, IFN-β, IFN-γ and TNF-α mRNA expression. mRNA expression was normalized to actin mRNA expression (n = 3). (E). BV2 cells were treated for 24 h with HFD metabolites (100 µL/mL) and with or without c-Myc inhibitor (10 µM). Total cell lysates were obtained from these cells were used to quantify the level of cGAMP by ELISA (n = 3). (F). BV2 cells were treated with cGAS (RU-521: 100 nM) and STING (C-176: 1 µM) inhibitors and stimulated with or without HFD brain metabolites. The culture supernatant was collected from these cells and the level of IFN-γ determined by ELISA. The values are expressed as mean ± SD for three independent assays. **p < 0.01, ***P < 0.001 compared with the untreated group using one-way ANOVA with Turkey's Multiple comparison test.

The results generated above further prompted us to test whether BASP1 interacts with c-Myc and regulates the STING expression in HFD mice by evaluating brain sections of lean, HFD and GaELNs treated HFD fed mice stained with fluorescent BASP1 and c-Myc; the interactions were determined using confocal microscopy. Surprisingly, no interaction of BASP1 with c-Myc was observed (Figure 6I). However, the mRNA level of BASP1 was significantly decreased in HFD mice compared to lean mice. Furthermore, the BASP1 level was increased in GaELNs treated HFD mice (Figure 6J). In contrast, the level of c-Myc was increased in HFD mice compared to lean mice and GaELNs treated HFD mice. A recent study has shown that calmodulin (CaM) plays a central role between BASP1 and v-Myc 58. BASP1 competes with v-Myc leading to destabilization of v-Myc and increased v-Myc degradation 58. Therefore, we tested whether GaELNs treatment can inhibit c-Myc activity via GaELNs competing with c-Myc for interaction with CaM thus blocking c-Myc activity in the HFD mouse brains. To test this hypothesis, we did confocal localization of BASP1 and CaM in the brain of lean, HFD and GaELNs treated HFD mice. Interaction between BASP1 and CaM was decreased in HFD mice compared to lean mice (Figure 7A). Furthermore, the GaELNs treated HFD mice showed increased interaction of BASP1 with CaM. In contrast, CaM and c-Myc interaction was significantly increased in HFD mouse brains compared to lean mouse brains. Interestingly, the c-Myc-CaM interaction was decreased in GaELNs treated HFD mice (Figure 7B). These results suggest that GaELNs treatment enhances the interaction of BASP1 with CaM and decreases the interaction of c-Myc with CaM.

STING promoter contains a c-Myc binding site and regulates the expression of STING 57. To determine whether metabolites derived from HFD mice could regulate STING promoter activity through c-Myc expression, we cloned the STING promoter containing the c-Myc binding site into the pGL3-basic luciferase vector and transfected BV2 cells. The cells were treated for 24 h with brain metabolites derived from lean and HFD mice and cells were treated with GaELNs, PA (36:4) and c-Myc inhibitor. Total cell lysates obtained from these cells were evaluated for luciferase activity. The STING promoter activity was significantly increased in HFD brain metabolite treated cells compared to lean metabolite treated cells (Figure 7C). The luciferase activity was significantly decreased in GaELNs, PA (36;4) and c-Myc inhibitor treated cells. Next, we sought to determine the role of c-Myc in regulation of inflammatory cytokines in brain microglial cells. We treated BV2 cells with HFD brain metabolites in the presence or absence of c-Myc inhibitor and quantified the level of mRNA expression of IFN-α, β, γ and TNF-α. Remarkably, c-Myc inhibitor significantly decreased inflammatory cytokine expression in HFD brain metabolite treated cells (Figure 7D). Next, we explored whether cGAS and STING are required for activation of inflammatory cytokines in HFD mice through c-Myc. BV2 cells were treated with HFD brain metabolites in the presence or absence of c-Myc inhibitor, GaELNs and PA (36:4) and a secondary messenger produced by cGAS, i.e., cGAMP, was quantified. The level of cGAMP was significantly increased in HFD metabolite treated cells and the level was significantly decreased in c-Myc inhibitor, GaELNs and PA (36:4) treated cells (Figure 7E). This result indicates that c-Myc increases cGAS activity in HFD mice. Additionally, GaELNs and PA (36:4) inhibit cGAS activity in these cells. To test whether cGAS and STING are required for inflammatory cytokine expression in these cells, BV2 cells were treated with HFD brain metabolites with or without cGAS and STING inhibitor RU-521 and C-176. The level of inflammatory and pro-inflammatory cytokines, e.g., IFN-γ, IL-6 and TNF-α, was significantly decreased in both cGAS and STING inhibitor treated cells (Figure 7F). Collectively, our results indicate that HFD induces microglia c-Myc which activates the cGAS-STING mediated inflammatory pathway. GaELN PA binds to BASP1, leading to inhibition of c-Myc expression and activity through competitively binding to CaM with c-Myc transcription factor.

## Discussion

Prolonged HFD-induced obesity is not reversable and causes many life-threatening diseases, including insulin resistance, oxidative stress and inflammation, hypertension and cardiovascular mortality 59, 60. Therefore, reversing the pathological processes underlying obesity and metabolic co-morbidities represents an essential task for biomedical research 61. In this study, our results suggest that oral administration of garlic exosome-like nanoparticles (GaELNs) reverses high-fat diet induced mouse obesity and insulin resistance. We can even reverse obesity of mice which have been fed a high-fat diet for more than one year. This therapeutic effect is through GaELN phosphatidic acid (PA) (36:4) mediated inhibition of the microglial c-Myc mediated cGAS/STING/IDO1/AHR inflammatory signaling cascade. The findings are summarized in the graphical abstract.

The microglial cell mediated chronic low-grade brain inflammation takes place in a number of diseases including obesity and type 2 diabetes 62, yet no effective therapy has been developed for prevention of microglial cell mediated brain inflammation. This study focused on the molecular mechanism(s) underlying how GaELNs contribute to the inhibition of microglial cell mediated inflammation in HFD induced obesity with insulin resistance developed. The findings from this study are significant for the development of future products that provide a basis for developing mechanism-driven novel approaches to prevent microglial cell mediated chronic brain inflammation via non-invasive oral administration of plant-based nanoparticles.

The finding that GaELNs can target to brain microglial cells in HFD fed mice is significant. HFD promotes chronic low-grade inflammation which is the major contributor for development of diabetes and insulin resistance in humans and mice 63. The inflammation is mediated by macrophages in the peripheral tissues and microglia, the innate immune cells of the brain 64. Microglia is a chronic source releasing multiple neurotoxic factors including TNF-α, nitric oxide and IL-1β and reactive oxygen species (ROS) which drive neuron damage 65. Our results show that microglial cells readily take up GaELNs, leading to inhibiting microglial cell activation and improving memory function in HFD fed mice. It is conceivable that besides the GaELN's anti-inflammation effect itself, GaELNs could be further developed as a therapeutic agent delivery vehicle to treat microglial related brain diseases.

The finding that plant GaELNs PA interacts with mouse brain acid soluble protein 1 (BASP1) is also meaningful. BASP1 is a neuron enriched Ca^2+^-dependent calmodulin (CaM)-binding protein. The BASP1 gene is a negative transcriptional target of Myc via its competing with Myc for calcium sensor CaM binding that leads to inactivation of v-Myc 56, 58. Myc+/- mice have significantly extended lifespans in both sexes and display ameliorated aging phenotypes across a variety of pathophysiological processes in multiple organs 57. The unifying aspect that translates from mice to humans is the diet regulated Myc signature. Dietary fat intake does not only amplify the Myc transcriptional program but can enrich for it 18, 19. The increased activation of c-Myc leads to enhanced mitochondrial membrane permeability which causes leakage of mitochondrial DNA into the cytosol and activates the cGAS/STING pathway to induce inflammatory cytokine expression 54, 66. Myc mediated-increases in reactive oxygen species (ROS) and DNA damage 16 could also induce the activation of the cGAS/STING inflammatory pathway 20, 21. We demonstrated that activation of c-Myc induces in microglial cells in HFD mice an array of inflammatory cytokines including IFN-γ via the cGAS/STING pathway. IFN-γ induces the expression of IDO1 to further activate the AHR signaling pathway via IDO1 metabolic products. Activation of AHR contributes to insulin resistance in HFD induced obesity in mice 67. Defects in PA production are involved in Alzheimer's disease 68. Our findings show that GaELN PA interacting with microglial BASP1 is required for targeting to microglial cells and subsequently inhibiting c-Myc mediated expression of STING. These findings provide a foundation to determine whether GaELN PA may not only be considered as a targeting moiety presented on nanovectors for the delivery of therapeutic agents to inhibit c-Myc activity in microglial cell via oral administration in general, but GaELN PA may itself be a therapeutic agent to treat brain defects of several pathological conditions.

Multiple pathways are dysregulated in obesity and type 2 diabetes. c-Myc, a multifunctional transcription factor, was activated in HFD mice 19. The individual pathophysiological role c-Myc, cGAS/STING, IDO1, and AHR plays in inflammatory responses has been demonstrated in obesity and type 2 diabetes 69-71. How one might target all of these molecules simultaneously without inducing side-effects is challenging. Compelling evidence shows that edible plants in a healthy diet have important physiological roles for normal brain function and prevent neuroinflammatory processes 72-77. In this study, we provided cellular and molecular insight into how healthy diet-derived exosome-like nanoparticles, i.e., GaELNs, modulate neuroimmune function via simultaneously targeting several molecules/pathways (Graphical abstract) that are dysregulated in microglial cells without inducing side-effects.

Our results also show that GaELNs not only inhibits STING gene expression in microglial cells via inhibition of c-Myc activity, but also targets to microglial cell mitochondria to prevent leakage of mtDNA that can cause activation of cGAS. It mediates the induction of cGAMP that serves as a ligand to activate STING. The mitochondrion are important intracellular organelles for drug targeting due to their key roles and functions in cellular proliferation and death 78. The significance of this finding provides the foundation for further identifying the GaELNs molecule that can target mitochondria so a potential non-invasive approach via oral administration is realized. Again, our results also show that the factors derived GaELNs itself has beneficial effect on restoring mitochondria homeostasis that is dysregulated in obesity mouse model. The expression of ROS related genes including Gpx 79, Duox1^80^, Rag2^81^, Nox1 82, Atr^83^, Ehd2 84 and Sod3 85 is altered as a result from GaELNs treatment. These molecules are known to play a critical role in mitochondria mediated cognitive function. Whether altering expression of these ROS related genes in microglial cells contributes to mitochondria mediated cognitive function in HFD mice requires further experimentation.

Our finding also implies that GaELNs regulate the microglial cell activity via IDO1-AHR axis signaling pathway. It has been shown that AHR plays an important role in HFD induced obesity, fatty liver, glucose intolerance and insulin resistance 86. AHR is a ligand-activated transcription factor that is activated by small molecules including kynurenines. Indoleamine 2,3 dioxygenase 1 (IDO1), a tryptophan metabolizing enzyme that converts the tryptophan into downstream catabolites known as kynurenines which acts as potential ligand for AHR 87. IDO1 enzyme is induced during inflammation by several immune factors including interferon-γ 88. In this study, we have shown that GaELNs inhibit the expression of IFN-γ in the brain. Further, we observed that GaELNs inhibits IDO1 mediated AHR activation in the microglial cells and improves the glucose tolerance and insulin sensitivity in HFD fed mice. In addition, we observed, knockout down of IDO1 in mice showed reverse the HFD phonotype in the mice. Collectively, these findings will provide a foundation for further investigating whether GaELN treatment could also be applied to treat disease caused by the AHR/IDO1/AHR pathway dysregulated.

Besides the brain, the liver plays a role in energy balance and glucose tolerance and insulin response^89^, ^90^. Our imaging data show that GaELNs are taken up by liver and intestine tissue as well. The results generated from this study will provide a rationale for further investigating how much of the efforts of GaELN targeted liver and intestine tissue may also contribute to reversing obesity in the HFD induced obesity mouse model.

In conclusion, in this study we made the following major discoveries. 1. Oral administration of GaELNs leads to inhibition of brain inflammation and the reversing of obesity in the obesity mouse model at least partly via a direct effect on the GaELN recipient microglial cells. 2. GaELN PA is required for targeting to microglial cells and subsequent interaction with microglial brain acid‐soluble protein 1 (BASP1). 3. Interaction of GaELN PA with microglial BASP1 results in more calmodulin recruited to BASP1, leading to a shortage of formation of c-Myc/calmodulin complex which is required for maintaining c-Myc activity. 4. We further show that expression and activity of c-Myc in microglial cells is increased in the obesity mouse model. 5. c-Myc contributes to brain inflammation by inducing an array of microglial-derived inflammatory cytokines via the cGAS/STING/IDO1/AHR inflammatory signaling cascade. Oral administration of GaELNs inhibits the cGAS/STING/IDO1/AHR inflammatory signaling cascade. 6. GaELNs migrate to microglial mitochondria and prevent mitochondria DNA leaking, which triggers activation of the cGAS/STING inflammation pathway.

## Materials and Methods

### Isolation and purification of garlic exosome-like nanoparticles (GaELNs)

Garlic exosome-like nanoparticles (GaELNs) were isolated and purified as described previously 48. Briefly, garlic was purchased from a local supermarket and washed with sterile PBS and the skin peeled away. Garlic was ground in a blender to obtain the juice and strained to remove larger particles. Juice was sequentially centrifuged at 1000×g for 10 min, 3000×g for 20 min and 10,000×g for 40 min to remove microparticles. The supernatant was then centrifuged at 150,000×g for 2 h, the pellet was resuspended in sterile PBS and transferred to a sucrose step gradient (8%/15%/30%/45%/60%), followed by centrifugation at 150,000×g for 2 h at 4°C 48. The bands between the 30%/45% layer were harvested separately and noted as garlic exosome-like nanoparticles (GaELNs). The purified GaELNs were fixed with 2% paraformaldehyde and imaged by electron microscopy (Zeiss EM 900) as described previously 48. GaELNs size and concentration (particle number) were determined using a NanoSight NS300 (Malvern Instrument, UK) at a flow rate of 30 µL per minute. The assays were performed in accordance with the manufacturer's instructions. Briefly, for the NanoSight, three independent replicates of diluted GaELNs preparations in PBS were injected at a constant rate into the tracking chamber using the provided syringe pump. The specimens were tracked at room temperature (RT) for 60 s. Shutter and gain were manually adjusted for optimal detection and were kept at optimized settings for all samples. The data were captured and analyzed with NTA Build 127 software (version 2.2, Malvern Instruments Ltd., Malvern, UK).

### Electron microscopy examination of GaELNs

GaELNs were fixed in 2% paraformaldehyde (Electron Microscopy Science, PA) in PBS for 2 h at 22 °C followed by 1% glutaraldehyde (Electron Microscopy Science, PA) for 30 min at 22 °C. 15 µL of fixed samples were put on a 2% agarose gel with formvar/carbon-coated nickel grids on top and allowed to adsorb for 5-10 min. The grids with adherent exosomes were fixed in 2% paraformaldehyde in PBS for 10 min followed by extensive washing in PBS. Negative contrast staining was performed with 1.9% methyl cellulose and 0.3% uranyl acetate for 10 min. The grids with negatively stained exosomes were dried before observation under a Zeiss EM 900 electron microscope 51.

### Cell culture

BV2 microglia cells were purchased from ATCC. The cells were maintained in high glucose Dulbecco's modified Eagle's medium (DMEM) supplemented with 10% fetal bovine serum, 100 units/mL penicillin, and 100 mg/mL streptomycin. Cells were maintained in humidified 5% CO_2_ at 37 °C.

### Primary neuronal cell culture

Primary neuronal cultures were prepared from the cerebral cortex of embryonic day 17 C57BL/6 mouse embryos 91. Briefly, the embryo cortices were removed, and meninges removed and placed in ice-cold HBSS. The cells were dissociated by trypsinization (0.25% for 15 min at 37°C), followed by trituration with a fire-polished Pasteur pipette and the cells were plated onto poly-D-lysine-coated 24 well plate containing DMEM with 5% fetal bovine serum. After 48 h, the media was changed to neurobasal media containing B-17 supplements. Cultures were maintained at 37 °C in a humidified incubator containing 5% CO_2_ and culture media were changed every 3 days.

### Animal Model

Male 10-12-week-old WT C57BL/6, IDO1^-/-^ and AHR^-/-^ mice were obtained from Jackson Laboratories, maintained in groups and housed in micro-isolator cages. Mice were a fed standard diet with water *ad libitum* and kept on a 12 h light and dark cycle. The University of Louisville Institutional Animal Care and Use Committee approved all animal procedures in this study. All animal experiments were performed according to institutional guidelines for animal care. The mice were matched according to age and divided into three groups. One group was fed regular chow diet (Lean) consisting of fat (4.5%), carbohydrate (56%) and protein (23%). A second group of mice was fed a high-fat diet (HFD) typical of a 'Western style' HFD. The HFD food contained fat (23%), carbohydrate (43%) and protein (23%). The normal chow diet and HFD were purchased from Harlan Labs Inc., Madison, WI). The animals were kept on these diets for more than a year to develop insulin resistance. After, confirming the HFD mice had developed insulin resistance and glucose intolerance, a third group of HFD fed mice were orally gavaged with garlic exosome-like nanoparticles (10^10^ particles) every day for six weeks. The glucose tolerance and insulin resistance tests were conducted as described.

### Glucose tolerance and insulin resistance test

Mice were fasted overnight before baseline blood glucose levels (mg/dl) were determined; 10 μl of tail vein blood were used in the glucose meter (Priology, USA). Glucose (2 mg dextrose/g body weight) in sterile PBS was injected intraperitoneally and blood glucose was measured at different time point (0,30, 60, 90, 120 min) after the injection 92. Insulin tolerance was conducted using the same glucometer. Mice were fasted for 6 h prior to starting the procedure. After the baseline glucose values were established, mice were given an intraperitoneal injection of insulin (1.2 units g^-1^ of body weight). Clearance of plasma glucose was subsequently monitored at the indicated times post-injection.

### Liver and brain histology

For hematoxylin and eosin (H&E) staining, liver and brain tissues from lean, HFD and GaELNs treated HFD mice were fixed overnight at 4°C in buffered 10% formalin solution (SF93-20; Fisher Scientific, Fair Lawn, NJ). Dehydration was achieved by sequential immersion in a graded ethanol series of 70, 80, 95, and 100% ethanol for 40 min each. Tissues were embedded in paraffin and subsequently cut 5 μm thick using a microtome. Tissue sections were deparaffinized in xylene (Fisher Scientific, Fair Lawn, NJ), rehydrated in decreasing concentrations of ethanol in PBS and tissue sections were stained with hematoxylin and eosin (H&E) for histological and morphological analysis. The slides were scanned with an Aperio ScanScope as previously described 93

### Biochemical assays

Whole blood was collected from lean, HFD and GaELNs treated HFD fed mice. Serum was separated from whole blood by centrifugation (1000×g for 10 min). The serum levels of aspartate aminotransferase (AST) and alanine aminotransferase (ALT) were measured using an Infinity enzymatic assay kit according to manufacturer's protocol (Thermofisher Scientific). High-density lipoprotein (HDL), total cholesterol (TC), and total triglyceride (TG) were determined using a Piccolo^R^ lipid panel plus (Abaxis Inc, CA, USA) according to manufacturer's instructions.

### *In vivo* imaging of GaELNs

To monitor the trafficking of GaELNs administered by oral gavage, GaELNs were first labeled with a near-infrared lipophilic carbocyanine dye, dioctadecyl-tetra methyl indotricarbocyanine iodide (DiR; Invitrogen, Carlsbad, CA), using a previously described method 94. Mice were anesthetized with ketamine (100 mg/kg body weight) and xylazine (10 mg/kg body weight) via i.p. injection, and inhaled isoflurane was used as necessary. The mice were imaged using a Pearl Impulse Molecular Imaging system (LI-COR Biosystem, Lincoln, NE). For controls, mice received non-labeled GaELNs in PBS at the same concentration as for DiR dye-labeled exosomes. Images were collected using a high-sensitivity CCD camera with an exposure time of 2 min. Regions of interest were analyzed for signals and were reported in units of relative photon counts per second. The total photon count per minute was calculated using Living Image software. The effects of DiR dye-labeled versus non-labeled GaELNs on mice were determined by dividing the number of photons collected for DiR dye-labeled GaELN treated mice by the number of photons collected for non-labeled GaELNs treated mice at different imaging time points.

### Determination of GaELNs uptake in brain

GaELNs were labelled with PKH26 and orally gavage (1 x 10^10^ particles) given to mice. After 4h, mice were perfused with saline to remove blood. Brain tissue was removed and fixed for 2 h at room temperature with periodate-lysine-paraformaldehyde (PLP) fixative, dehydrated with 30% sucrose solution overnight at 4 °C, embedded in OCT and cut into 8 μm sections. The tissue sections were blocked with 5% BSA at room temperature for 1 h and then incubated for 3 h at room temperature with anti-IBA-1 antibody (1:100) and anti-β-Tubulin III antibody (1:100). After washing three times, tissue sections were stained with Alexa 488-conjugated secondary antibody (1:500) for 1 h at room temperature and the nucleus stained with DAPI. The tissue slides were mounted and viewed using a confocal microscope equipped with a digital image analysis system (Nikon, Melville, NY).

### Extraction of brain metabolites and cell culture treatment

To obtain protein-free extract of metabolites derived from lean, HFD and GaELNs treated HFD mice brain we used a flowing sample preparation protocol. Mice were anesthetized with ketamine (100 mg/kg body weight) and xylazine (10 mg/kg body weight) via i.p. injection and mice were perfused as previously described 95. Then the brain was removed and weighed. 100 mg of tissue was homogenized using a TissueRuptor II homogenizer in 1 mL of ice-cold water/methanol/chloroform mixture at a ratio of 1:2:2, vortexed for 30 s, kept on ice for 10 minutes and incubated at -20 ^0^C for 20 min. The mixture was centrifuged at 12,000 rpm at 4 °C for minutes to pellet protein. The top hydrophilic fraction was collected into fresh vials and stored at -80 °C for further use. BV2 cells were treated for 24 h with and without brain metabolites derived from lean, HFD and GaELNs treated mice brain (100 µL/mL). Total cell lysates obtained from these cells were subjected to western blot analysis and total RNA isolated from these cells were subjected to real-time PCR for mRNA expression.

### Flow cytometry analysis

GaELNs were labelled with PKH26 and orally gavage (1 x 10^10^ particles) given to mice. After 4 h, mice were perfused with saline to remove blood. Liver and gut tissues were removed and cells were isolated as described earlier 94, 96. For cell surface marker staining, isolated cells were blocked with 10 µg/mL mouse Fc block for 5 min at 4 °C (BD Biosciences, San Jose, CA) and then reacted with various fluorochrome-labeled antibodies including appropriate isotype controls for 1 h at 4 °C. After washing three time, cells were fixed and analyzed using a FACSCalibur flow cytometer (BD Biosciences). Data were analyzed using FlowJo software (TreeStar, Ashland, OR).

### Determination of Blood-brain barrier (BBB) function assay

Disruption of BBB integrity was determined using FITC-BSA as described 97. Briefly, FITC-BSA was intraperitoneally injected into lean, HFD and GaELNs treated HFD fed mice. After 1 h, mice were perfused with saline to remove blood. The brain was rapidly harvested and embedded in OCT compound and the frozen brain was stored at -80 °C until cryostat sectioning. The brain was serially cut into 10 µm thick sections and mounted on microscopic slides. Nuclear staining was done with DAPI and localization of FITC-BSA was visualized using confocal microscopy (Nikon, Melville, NY). In addition, we measured the fluorescent intensity in the brain. Brain tissue was homogenized in cell lysis buffer and the FITC fluorescent intensity was determined using a microplate reader (microTek, San Jose, CA) set at an excitation of 495 nm and emission wavelength of 519.

### Lipid extraction and TLC analysis

Total lipids from GaELNs were extracted with chloroform: methanol (2:1, v/v). GELNs were mixed with chloroform: methanol and the mixture vortexed 98. The mixture was then centrifuged at 2000×g for 10 min and the bottom layer containing lipids carefully removed and completely dried under nitrogen. Then, thin-layer chromatography (TLC) was performed. HPTLC-plates (silica gel 60 with concentrating zone, 20 cm × 10 cm; Merck) were used for the separation. Aliquots of concentrated lipid samples extracted from GaELNs were separated on HPTLC-plates with chloroform: methanol: H_2_O (65:25:4) as the mobile phase. After drying, lipids were stained using iodine vapor. The plate was imaged with an Odyssey Scanner.

### Lipidomic analysis

Lipid samples extracted from GaELNs and band 3 from TLC was excised and submitted to the Lipidomics Research Center, Kansas State University (Manhattan, KS) for analysis 99. The lipid composition was determined using a triple quadrupole mass spectrometer (Applied Biosystems Q-TRAP, Applied Biosystems, Foster City, CA). The data are reported as concentration (nmol/mg GELNs) and percentage of each lipid in total signal for the molecular species determined after normalization of the signals to internal standards of the same lipid class. Multiple correlation analysis was used to predict changes in the lipid content and which lipids were most affected.

### Lipid nanoparticle preparation

GaELNs total lipids and the band of excised lipids from TLC and PA (36:4) were dried under a stream of nitrogen gas and then under vacuum overnight. The lipid film was suspended in HBS running buffer (20 mM HEPES, 150 mM NaCl, pH 7.4), gently vortexed and sonicated for 10 min until a clear solution was formed. The lipid nanoparticle suspension was extruded through a polycarbonate membrane filter syringe with a pore size of 100 nm. The size of the lipid nanoparticles was confirmed using a NanoSight NS300 (Malvern Panalytical Inc, MA, USA). Total lipids were determined by measuring total phosphate level 100.

### Isolation of RNA from GaELNs

Total RNA was isolated from GaELNs using a RNeasy mini kit (Qiagen) according to the manufacturer's instructions. In brief, 100 mg of GaELNs was disrupted in QIAzol Lysis Reagent. The homogenate was mixed with 140 μL of chloroform and centrifuged at 12,000 rpm for 15 min at 4 °C. The upper aqueous phase was mixed with 1.5 volumes of ethanol and loaded into a RNeasy spin column. The flow-through was discarded after centrifugation, and the column was washed with RWT and RPE sequentially. Total RNA was eluted with RNase-free water. The quality and quantity of the isolated RNA were analyzed using a NanoDrop spectrophotometer and Agilent Bioanalyzer.

### Preparation of GaELNs RNA libraries and sequencing

Small RNA libraries were generated with 100 ng of total RNA from GaELNs and TruSeq Small RNA Library Preparation Kits (Illumina, San Diego, CA) according to the manufacturer's instructions. Following PCR amplification (16 cycles), libraries between 140 and 160 bp in size were gel purified and resuspended in ultrapure water. Equal amounts of libraries were pooled and sequenced on an Illumina HiSeq 2500, followed by demultiplexing and fastq generation with CASAVA v1.8.4. Raw fastqs were adapter and quality score trimmed with cut adapt v1.10. with a minimum length of 15 nucleotides. MicroRNAs were identified using the sRNABench Pipeline (version 05/14). A core set of plant mRNAs from miRBase v21 was used as a reference and this set included all 14 plant species with at least 200 mature microRNA sequences annotated in miRBase. Within the sRNABench pipeline, mapping was performed with bowtie (v0.12.9) and microRNA folding was predicted with RNAfold using the Vienna package (v2.1.6).

### Delivery of RNA into BV2 cells

Total RNA isolated from GaELNs was packaged into lemon-derived lipid nanovectors. Lemon lipids were extracted with chloroform, methanol and dried under vacuum. Then, 200 nM of lipid was suspended in 250 µLµL of 155 mM NaCl with or without 10 µg GaELNs derived RNA. After UV irradiation at 500 mJ/cm^2^ in a spectrolinker crosslinker (Spectronic Corp) and sonication (FS60 bath sonicator, Fisher Scientific) was continued for 30 min. Then, the complexes were centrifuged at 100,000 g for 1 h at 4 °C. The RNA encapsulation efficiency was determined using previously described method 22. BV2 cells were treated with the RNA bound lipid nanoparticles for 24 h. Then, total RNA was isolated from these cells and determined IDO1 mRNA expression by real-time PCR.

### 2DLC-MS/MS analysis and its data analysis

The supernatant and total cell lysates derived from the brain of lean, HFD and GaELN treated HFD mice was mixed with 3 volumes of methanol for 30 min to precipitate protein. The supernatant was subjected to MS/MS analysis for quantification of metabolites. To extract polar metabolites for 2DLC-MS/MS analysis, 500 µL of supernatant was lyophilized, then redissolved in 100 µLµL 20% acetonitrile. After 3 min of vigorous vortex mixing, the sample was centrifuged at 12,000×g for 20 min at 4 °C. The supernatant was collected and used for 2DLC-MS/MS analysis 101.

All samples were analyzed on a Thermo Q Exactive HF Hybrid Quadrupole-Orbitrap Mass Spectrometer coupled with a Thermo DIONEX UltiMate 3000 HPLC system (Thermo Fisher Scientific, Waltham, MA, USA). The UltiMate 3000 HPLC system was equipped with a hydrophilic interaction chromatography (HILIC) column and a reverse phase chromatography (RPC) column. The HILIC column was a SeQuant® ZIC®-cHILIC HPLC column (150 × 2.1 mm i.d., 3 µm) purchased from Phenomenex (Torrance, CA, USA). The RPC column was an ACQUITY UPLC HSS T3 column (150 × 2.1 mm i.d., 1.8 µm) purchased from Waters (Milford, MA, USA). The two columns were configured in parallel 2DLC mode.

For 2DLC separation, the mobile phase A for RPC was water with 0.1% formic acid and the mobile phase A for HILIC was 10 mM ammonium acetate (pH adjusted to 3.25 with acetate). Both RPC and HILIC used the same mobile phase B, acetonitrile with 0.1% formic acid. The RPC gradient was 0 min, 5% B, hold for 5.0 min; 5.0 min to 6.1 min, 5% B to 15% B; 6.1 min to 10.0 min, 15% B to 60% B, hold for 2.0 min; 12.0 min to 14.0 min, 60% B to 100% B, hold for 13.0 min; 27.0 min to 27.1 min, 100% B to 5% B, hold for 12.9 min. The HILIC gradient was 0 to 5.0 min, 95% B to 35% B, hold for 1.0 min; 6.0 min to 6.1 min, 35% B to 5% B, hold for 16.9 min; 23.0 min to 23.1 min, 5% B to 95% B, hold for 16.9 min. The flow rate was 0.4 mL/min for RPC and 0.3 mL/min for HILIC. The column temperature was 40°C for both columns. The injection volume was 2 µL.

To avoid systemic bias, the samples were analyzed by 2DLC-MS in a random order. All samples were first analyzed by 2DLC-MS in positive mode followed by 2DLC-MS in negative mode to obtain the full MS data of each metabolite. For quality control purposes, a pooled sample was prepared by mixing a small portion of the supernatant from each sample and was analyzed by 2DLC-MS after injection of every six biological samples. The pooled sample was also analyzed by 2DLC-MS/MS in positive mode and negative mode to acquire MS/MS spectra for metabolite identification.

For 2DLC-MS data analysis, MetSign software was used for spectrum deconvolution, metabolite identification, cross-sample peak list alignment, normalization, and statistical analysis. To identify metabolites, the 2DLC-MS/MS data of pooled sample were first matched to our in-house MS/MS database that contains the parent ion m/z, MS/MS spectra, and retention time of 187 metabolite standards. The thresholds used for metabolite identification were MS/MS spectral similarity ≥ 0.4, retention time difference ≤ 0.15 min, and m/z variation ≤ 4 ppm. The 2DLC-MS/MS data without a match in the in-house database were then analyzed using Compound Discoverer software (Thermo Fisher Scientific, Inc., Germany), where the threshold of MS/MS spectra similarity score was set as ≥ 40 with a maximum score of 100. The remaining peaks that did not have a match were then matched to the metabolites in our in-house MS database using the parent ion m/z and retention time. The thresholds for assignment using the parent ion m/z and retention time were ≤ 4 ppm and ≤ 0.15 min, respectively.

### Surface Plasmon Resonance (SPR)

SPR experiments were conducted on an OpenSPR^TM^ (Nicoya, Lifesciences, ON, CA). All experiments were performed on a LIP-1 sensor (Nicoya, Lifesciences) as described earlier 51. Tests were run at a flow rate of 20 µL/min using HBS running buffer (20 mM HEPES, 150 mM NaCl, pH 7.4). First, the LIP-1 sensor chip was cleaned with octyl β-D-glucopyranoside (40 mM) and CHAPS (20 mM). Liposomes were made from GaELN total lipids and PA (36:4). Liposomes (1 mg/mL) were injected on the sensor chip for 10 min until stable resonance was obtained. After immobilization of lipid nanoparticles, the surface was blocked with BSA (3%) in running buffer. After a stable signal was obtained, total cell lysates (5 µg/mL of protein) from the brain of HFD mice were run over the immobilized liposomes. A negative control test was also performed by injecting protein onto a blank sensor chip to check for non-specific binding. After 10 min, the lipid nanoparticle binding protein was eluted using NaOH (200 µM). The eluted protein was subjected to LC-MS proteomic analysis for identification of GaELN lipid nanoparticles and PA binding proteins. The sensograms were analyzed using TraceDrawer kinetic analysis software.

### Lipid nanoparticle pull-down assay

GaELN lipid and PA (36:4) interactions with BASP1 were further confirmed using a lipid nanoparticle pull-down assay 51, 102. GaELN and PA (36:4) lipid nanoparticles were fluorescently labelled with PKH26 in vesicle buffer (50 mM Tris-HCl (pH 7.5) and 150 mM NaCl). The labelled nanoparticles were incubated overnight at 4 °C with rotation in total cell lysates derived from HFD fed mice brain. Then, the complex was incubated with anti-BASP1 antibody for 3 h with rotation at room temperature. The lipid-protein complexes were pulled down by protein agarose beads. The complexes were washed thoroughly, and lipid nanoparticles were eluted from the complexes using elution buffer (100 mM glycine). The lipid nanoparticles were quantified using flow cytometry. The number of lipid nanoparticles in the complex was determined using a Nanosight 300 and the quantity of lipid nanoparticles was determined based on fluorescent intensity as measured using a fluorescence spectrophotometer (Molecular Device) with excitation and emission wavelengths of 551 and 567 nm, respectively.

### Liquid chromatography-mass spectrometry (LC-MS) data analysis

Proteome Discoverer v1.4.1.114 (Thermo Fisher Scientific,) was used to analyze the data collected by the mass spectrometer. The database used in Mascot v2.5.1 and SequestHT searches was the 2/17/2017 version of the mouse proteome from UniprotKB. Scaffold was used to calculate the false discovery rate using the Peptide and Protein Prophet algorithms. Proteins were grouped to satisfy the parsimony principle. The proteins were clustered based on differential expression and heat maps representing differentially regulated proteins by GaELNs were generated using software R.

### Western blot

Total cell lysates were prepared in RIPA lysis buffer (50 mM Tris HCl, 150 mM NaCl, 1.0% (v/v) NP-40, 0.5% (w/v) sodium deoxycholate, 1.0 mM EDTA, 0.1% (w/v) SDS and 0.01% (w/v) sodium azide, pH of 7.4.) with protease and phosphatase inhibitors (Roche). Cell lysates were separated by SDS-PAGE (4-5% gradient gel) and transferred onto nitrocellulose membranes. After transfer, membranes were probed for overnight at 4 °C with primary polyclonal antibodies specific for BASP-1 at 1:1000. The membrane was washed with PBS-T and incubated for 1 h at room temperature with secondary antibodies conjugated to Alex-647 (Eugene, OR, USA) at 1:10,000. Bands were visualized and analyzed based on band intensity using Odyssey Imager (Licor Inc, Lincoln, NE, USA).

### Real-time qPCR

Total RNA was isolated from tissue and cell lines using TRIzol reagent according to the manufacturer's protocol (Invitrogen). RNA (1 µg) was converted into cDNA with an iScript cDNA synthesis kit (Bio-Rad, Hercules, CA). cDNA samples were amplified with Sso Fast green super mix using the CFX96 Real-time PCR system (Bio-Rad). mRNA expression was quantified by the ΔΔCt method using 16S rRNA expression as an internal control for bacterial gene expression and actin expression as an internal control for mouse gene expression. All primers were purchased from Eurofins MWG Operon and primers are listed in Table S8.

### Determination of motor function - Rotarod Test

Motor function in lean, HFD and GaELNs treated HFD fed mice was measured using a Panlab LE 8205 RotaRod System (Harvard Apparatus, Spain) according to a previously published protocol 103. In brief, experimental mice were acclimated to the device over 2 practice sessions consisting of 1 session per day with 3 trials of varied protocols per session. This was followed by a testing day where the mice ran on the device as it accelerated from 4 to 40 rpm over 5 min; the outcome measure was latency to falling. We report the best of three trials, each trial administered with a minimum 15 min rest period.

### Determination of neuromuscular function - grip test

The neuromuscular function was determined by the grip strength test as maximal muscle strength of combined forelimbs and hind limbs by using a grip force tester (Grip Strength Tester bio-GT3, Bioseb, Vitrolles, France) following the protocol of Larcher et al 104. Experimental mice were placed on their forepaws on a grid and were gently pulled backward until they released their grip 103. The grip meter, attached to a force transducer, measured the peak force generated. Five tests were performed in sequence with a short rest period between each test, and results were expressed in grams.

### Determination of sensorimotor coordination - Balance Beam Test

Balance beam testing was performed to determine the sensorimotor coordination and forelimb and hind limb functionality. For the balance beam test 105, mice were placed on a metal beam 1.3 cm diameter and 77 cm long, suspended 2 feet above the ground, and were required to traverse the beam. They were scored on a scale of 0 - 6 as follows: 0, crosses the beam without any slips or hesitations; 1, crosses the beam with 1 or 2 slips and/or hesitation; 2, crosses the beam partially with multiple slips and falls; 3, balances with steady posture (>60 s) with multiple slips and one fall; 4, attempts to balance on the beam and falls off (>40 s) with multiple slips and falls but walks on paws; 5, attempts to balance on the beam but falls off (>20 s), multiple falls and fails to walk on the feet; and 6, falls off: no attempt to balance or hang on the beam (>20 s). A score of 1 indicated strong coordination and gait, whereas a score of 6 indicated very poor performance**.**

### Assessment of behavior assay- Novel Object Recognition Test (NORT)

The Novel Object Recognition Test (NORT) is a commonly used behavioral assay for the investigation of various aspects of learning and memory in mice 106. In brief, mice were placed individually in a testing chamber with beige walls for a 5 min habituation interval and returned to their home cage. Thirty minutes later mice were placed in the testing chamber for 10 min with two identical objects (acquisition session). Mice were returned to their home cages and one day later placed back into the testing chamber in the presence of one of the original objects and one novel object (recognition session) for 5 min. The chambers and objects were cleaned with ethanol between trials. Exploratory behavior was defined as sniffing, touching and directing attention to the object. Expected normal behavior would be, with a short delay between Acquisition and Retention trials, that the animal explores the novel object for a longer period than the familiar object. A “memory score is calculated for each animal, defined as the time spent in exploring the novel object as a percentage of total time exploring both objects during the retention trial.” For the acquisition session, the recognition index (RI) was calculated as (time exploring one of the objects/the time exploring both objects). For the recognition session, the RI was calculated as (time exploring the novel object/the time exploring both the familiar and novel object). Discrimination index (DI) was also calculated (DI = (Novel Object Exploration Time/Total Exploration Time) - (Familiar Object Exploration Time/Total Exploration Time) ×100). In our laboratory, NORT was performed using a Top Scan behavioral analyzing system (Version 3.00 by Clever Sys Inc; Reston, VA, USA). The data for the behavioral assessment was expressed by calculating DI. A lower DI indicates less time spent with the novel object; thus, indicates short-term memory impairment 107. Overall, the NORT is a relatively low-stress, efficient testing for memory in mice, and is appropriate for the detection of neuropsychological changes following pharmacological, biological, or genetic manipulations 106.

### TUNEL assay

The *in vivo* apoptotic cell death in brain of lean, HFD and GaELNs treated HFD fed mice was determined using a fluorescence TUNEL detection kit according to the manufacturer's instructions (Roche Applied Science, Indianapolis, IN). In brief, 10-μm-thick frozen sections of brain from lean, HFD and GaELNs treated HFD fed mice were cut using a cryostat and kept at -20 °C until further use. The tissue section was brought to room temperature and air dried, fixed with 4% paraformaldehyde for 20 min at room temperature. Then, permeabilized with 0.2% Triton X-100 for 10 min, the TUNEL reaction mixture added, and the slide incubated in a humidified atmosphere for 60 min at 37 °C. The nucleus was stained with DAPI. Sections were mounted using mounting solution and apoptotic cell death was visualized by confocal microscopy. The number of TUNEL positive and DAPI nuclei was calculated, and data were expressed as percentage of TUNEL positive cells, which was calculated by counting the number of TUNEL-positive nuclei divided by the total number of nuclei multiplied by 100.

### 2'-3'-cGAMP enzyme immunoassay

Total cell lysates were prepared from brain tissue of lean, HFD and GaELNs treated HFD mice and BV2 cells were treated with metabolites derived from brain tissues. The level of cGAMP in cell lysates was measured by ELISA according to manufacturers' instructions (Arbor Assays, An Arbor, MI). The results were normalized based on total protein concentration.

### Measurement of mitochondrial membrane potential

Mitochondrial membrane potential (ΔΨ) was determined using JC-10™ (AAT Bioquest, Sunnyvale, CA), a cationic and lipophilic dye that selectively enters mitochondria. The mitochondrial uptake of JC-10™ is directly associated with ΔΨ. In general, JC-10™ accumulates in the mitochondrial matrix where it forms red fluorescent aggregates. In uncoupled cells, JC-10™ diffuses out of the mitochondria and changes to monomeric forms and stains the cell a green fluorescence. The primary neuronal cells were suspended in 1 mL culture medium with JC-10™ (7.5 μM) for 30 min at 37 °C, 5% CO_2_. The JC-10™ stained cells were centrifuged (1200×g, 5 min) and the pelleted cells were washed three times with 1 mL PBS containing FBS (3%). The stained cells were resuspended in PBS. The fluorescent intensities for aggregate and monomeric forms of JC-10™ were measured at Ex/Em = 490/525 nm (FITC channel) and 540/595 nm (TRITC channel) in triplicate using 200 μL of stained cell suspension in a 96-well fluorometric plate reader (SpectraMax i3, Molecular Devices). The aggregate/monomer ratio was calculated and was proportional to the mitochondrial membrane potential 108.

### Determination of mtDNA released into the cytosol

BV2 cells were treated for 24 h with brain metabolites derived from lean, HFD and GaELNs treated HFD fed mice. Cells were divided into two equal aliquots with one aliquot being resuspended in 50 mM NaOH and boiled for 10 min to solubilize the total DNA. After completing this step, Tris-HCl (pH 8) was added and total mtDNA was isolated to serve as a normalization control. The second aliquot was resuspended in lysis buffer containing 150 mM NaCl, 50 mM HEPES (pH 7.4), digitonin (25 mg/mL) for 10 min, then centrifuged at 100×g for 10 min. The cytosolic fraction of the supernatant was transferred to a new tube and centrifuged at 17,000×g for 10 min to remove all cellular debris. DNA was isolated from these cytosolic fractions using a Qiagen DNA extraction kit. DNA isolated from the cytosol and whole cell extract was subjected to real-time PCR for mtDNA specific primer cytochrome c oxidase 109.

### MTT assay

The viability of cells was determined by the MTT assay. Briefly, cells were added with 100 µL of MTT (3-(4,5-Dimethylthiazol-2yl)-2,5-diphenyltetrazolium bromide) reagent (500 ng/mL) to each well and incubated for 30 min at 37 °C. Then, removed MTT solution and washed with sterile PBS and add 180 µL of DMSO and incubate for 15 min to dissolve the formazan crystals. The color intensity was measured spectrophotometrically at 560 nm.

### Lentivirus production and transduction

Lentiviral IDO1 and BASP-1 CRISPR/Cas9 KO plasmids were used to transiently transfect HEK (human embryonic kidney) 293T cells with lentivirus packing vectors pCMV-Δ8.2 and VSV-G plasmids using the Lipofectamine 3000 transfection kit (Invitrogen, USA) and virus containing medium was collected as described earlier 110. BV2 cells were transduced for 72 h with virus containing IDO1 and BASP1 in the presence of polybrene. The stable cell lines were established by selecting for resistance to puromycin (1.5 µg/mL) and the concentration of puromycin was determined by a puromycin kill curve assay. Similarly, a non-targeting control cell line was established by transfecting the cells.

### Cloning of mouse STING promoter region

The c-Myc binding site and deletion of c-Myc binding site in the STING promoter region was amplified by the polymerase chain reaction (PCR) and then cloned into *KpnI* and *Bgl II* sites of the pGL3-Basic vector (Promega) 21. The forward primer was 5'-GTA*GGTACC*TGAAACTATTAAACCTTGC-3' and the reverse primer 5'-GATC*AGATCT*CTTCTGCTTCCTAGACCGGT-3' (restriction sites are underlined). Similarly, the c-Myc binding site deletion clones were constructed using PCR of the above reverse primer and forward primer 5'- GTA*GGTACC*TGGAAATCCTGTGGGGCCC-3'.

### Transient transfection and luciferase reporter assay

BV2 cells were transiently transfected with mouse c-Myc promoter (Addgene, Watertown, MA), STING promoter and c-Myc binding site deleted STING promoter using the Lipofectamine-3000 transfection reagent (Invitrogen). After 24 h, the cells were treated for 24 h with 100 µL of metabolites derived from lean, HFD and GaELNs treated HFD mice brains. Luciferase activity was measured using a dual-luciferase system (cat. No. E1910, Promega Corp. WI, USA) as per the manufacturer's instructions.

### Nuclear and cytosolic fraction extraction

Nuclear and cytoplasmic extracts were prepared as described previously 111. Nuclear and cytoplasmic fractionation was confirmed by western blotting for a cytoplasmic marker (β-actin) and a nuclear marker (histone). Extracts were either used immediately or stored at -80 °C. For whole-cell lysate preparation, cells were mixed with cell lysis buffer (50 mM Tris-HCl (pH 7.4), 150 mM NaCl, 1 mM EDTA, 1% Triton X-100, 0.5% IGEPAL CA630) containing protease inhibitor cocktail (Roche Diagnostics), incubated at 4 °C for 30 min, and the supernatant was collected after centrifugation at 5000 rpm for 5 min.

### Mouse cytokine array

To determine the effect of GaELNs on the regulation of cytokine production in the plasma and brain tissues of lean, HFD and GaELNs treated HFD mice. Plasma and brain cell lysates were processed as per the manufacturer's protocol. Cytokines were analyzed with a Proteome Profiler Mouse XL Cytokine Array Kit (R&D Systems, RY028) as per the manufacturer's instructions. Quantification of the spot intensity in the arrays was conducted with background subtraction using HL Image++ (Western Vision Software).

### Cytokine detection

TNF-α, IL-6, IL-1β and IFN-γ in culture supernatants and brain homogenates were quantified using ELISA kits (eBioscience).

### Microglial depletion

For microglial depletion, Clodrosomes^R^ (Encapsula Nano Sciences) was used in accordance with the manufacturer's instructions. In brief, a parenchyma injection 30 of Clodrosomes^R^ was given to each mouse and 72 h later microglial depletion was confirmed by FACS analysis of IBA-1 positive brain microglial cells.

### Statistical analysis

Values are shown as mean ± SD for three independent experiments. Statistical analysis was performed with GraphPad Prism 6. Comparison of multiple experimental groups was performed using the one-way Analysis of Variance test with Tukey post hoc test for multiple comparisons. A t-test was used to compare the means of two groups. P values of < 0.05 were statistically significant. Appropriate sample sizes were calculated to ensure statistical significance could be determined.

## Supplementary Material

Supplementary figures and tables.
