# Supplementary material for: Garlic exosome-like nanoparticles reverse high-fat diet induced obesity via the gut/brain axis
Source: Theranostics. 2022 Jan 1;12(3):1220–46. doi: 10.7150/thno.65427 (PMC8771565; doi:10.7150/thno.65427)
Supplement: Supplementary file 1 — Supplementary figures and tables. [file thnov12p1220s1.pdf]

Supplemental Information

Garlic exosome-like nanoparticles reverse high-fat diet induced obesity via the gut/brain axis

Kumaran Sundaram[1], Jingyao Mu[1], Anil Kumar[1], Jyotirmaya Behera[2], Chao Lei[1], Mukesh K Sriwastva[1], Fangyi Xu[1], Gerald W Dryden[1,3], Lifeng Zhang[1], ShaoYu Chen[4], Jun Yan[1], Xiang Zhang[4], Juw Won Park[5,6], Michael L Merchant[7], Neetu Tyagi[2], Yun Teng[1], and Huang-Ge Zhang[1,8,9*]

[1] James Graham Brown Cancer Center, Department of Microbiology & Immunology, University of Louisville, KY 40202, USA

[2] Department of Physiology, University of Louisville School of Medicine, Louisville, KY 40202, USA.

[3] Department of Medicine, University of Louisville, Louisville, KY 40202, USA

[4] Department of Pharmacology and Toxicology, University of Louisville, Louisville, KY 40202, USA

[5] Department of Computer Engineering and Computer Science, University of Louisville, KY 40202, USA

[6] KBRIN Bioinformatics Core, University of Louisville, Louisville, KY 40202, USA

[7] Kidney Disease Program and Clinical Proteomics Center, University of Louisville, Louisville, KY 40202, USA

[8] Robley Rex Veterans Affairs Medical Center, Louisville, KY 40206, USA

[9] Lead contact

CONTACT FOR REAGENT AND RESOURCE SHARING

Further information and request for reagents may be directed to lead contact,

Dr. Huang-Ge Zhang (h0zhan17@louisville.edu).

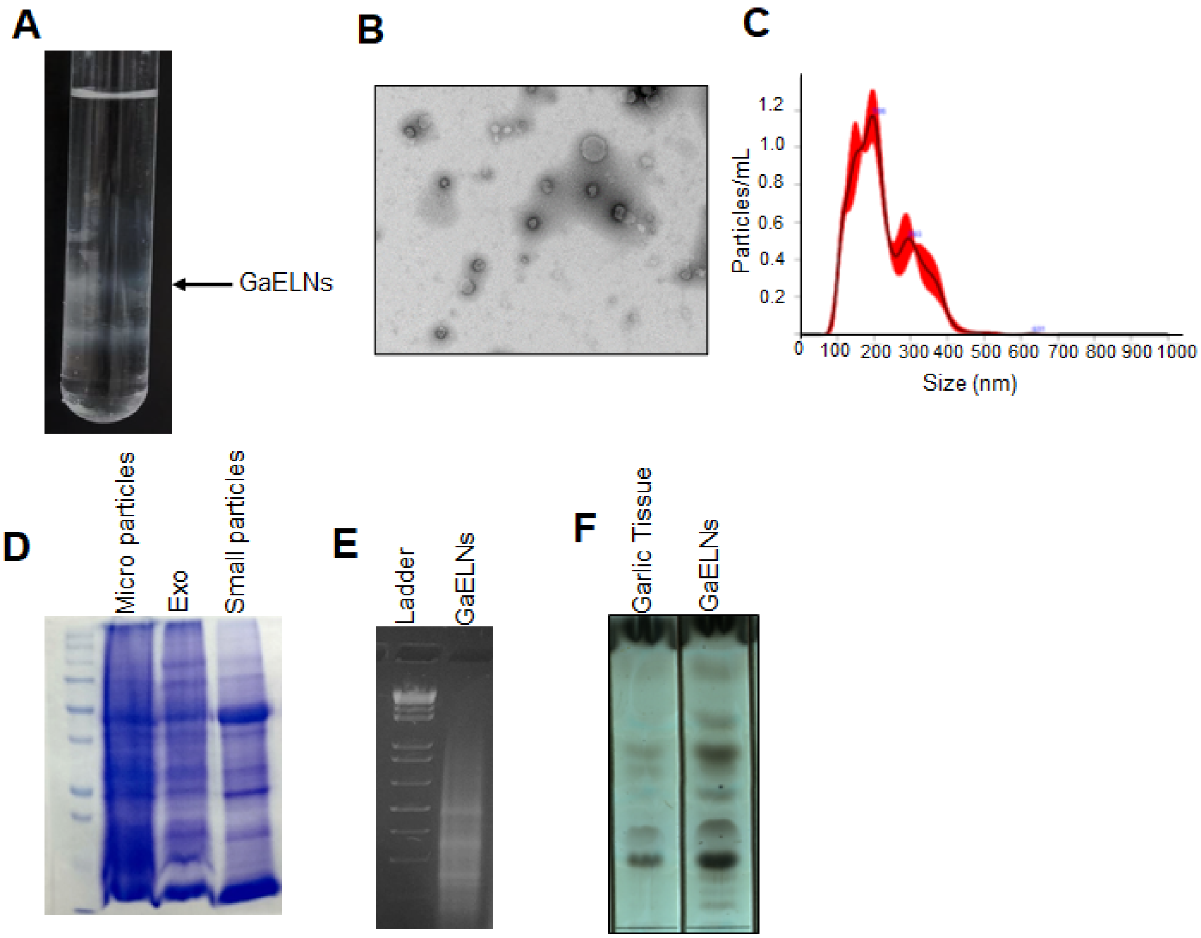

Figure S1: Isolation, purification and characterization of garlic exosome-like nanoparticles (GaELNs). (A). Garlic exosomes like nanoparticles were isolated by ultracentrifugation and purification of GaELNs by sucrose gradient centrifugation as described in methods (B). The purified GaELNs were viewed under electron microscopy. (C). The GaELNs size was determination using the Nanosight NS300. (D). Different size the of the garlic nanoparticles were lysed with cell lysis buffer and subjected to SDS-PAGE and protein was stained with Coomassie brilliant blue. (E). Total miRNA was isolated from GaELNs and subjected to agarose gel electrophoresis. (F). Total lipids were extracted from the GaELNs and separated by thin layer chromatography as described in methods.

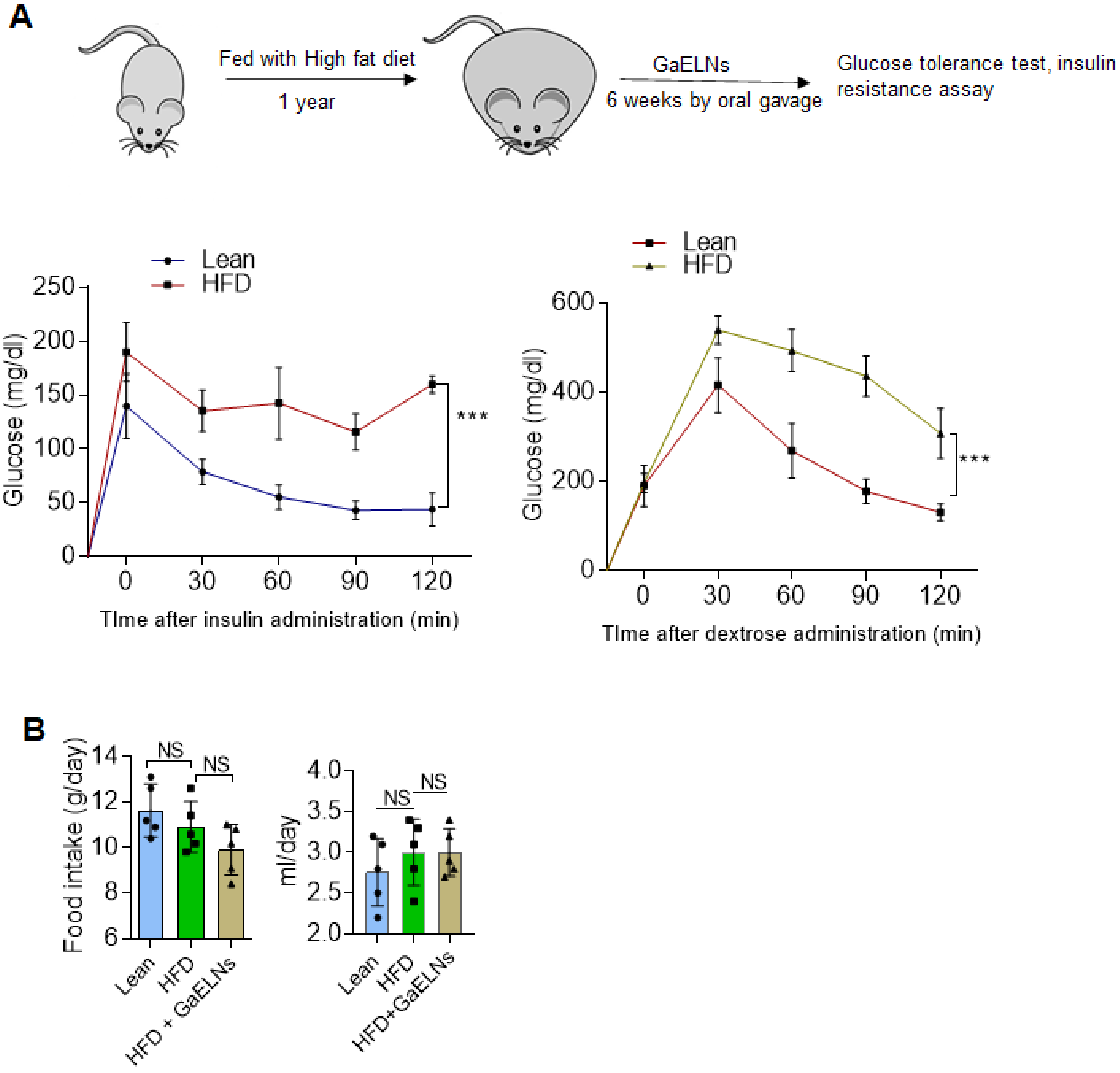

Figure S2: (A). Schematic representation of high-fat diet fed and GaELNs treatment in C57BL/6 mice. (B). Glucose tolerance test. (B). Insulin resistance assay. (C). Food and water intake of lean, HFD and GaELNs treated HFD fed mice.

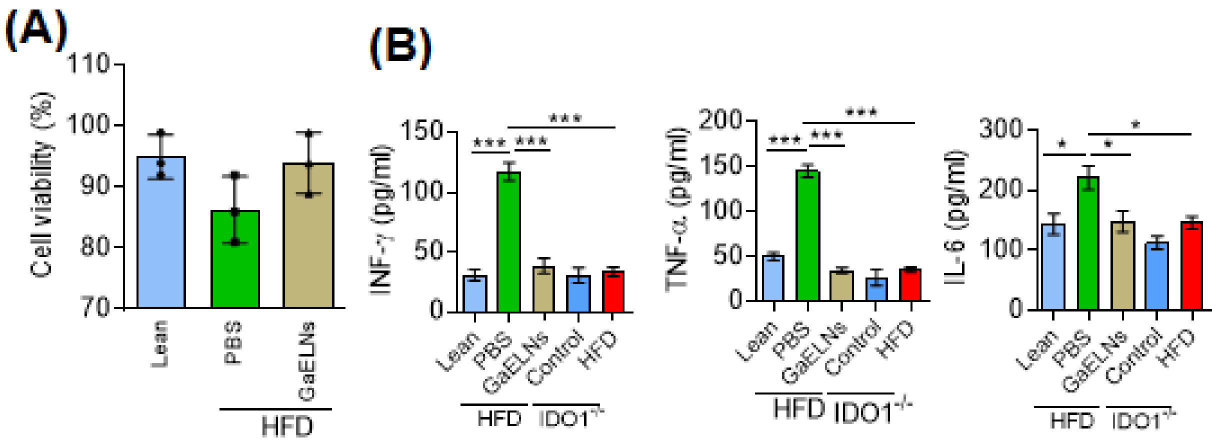

Figure S3. (A). BV2 cells were treated with brain metabolites (100 µl/ml) derived from lean, HFD and GaELNs treated HFD mice for 24 h. Cell viability was determined by MTT assay as described in methods. (B). BV2 cells were treated with metabolites derived from lean, HFD fed mice, GaELNs treated HFD fed mice, IDO1$^{-/-}$ control and HFD fed IDO1$^{-/-}$ mice for 24 h. The culture supernatant was collected from these cells and the level of IFN-γ, TNF-α and IL-6 was quantified by ELISA.

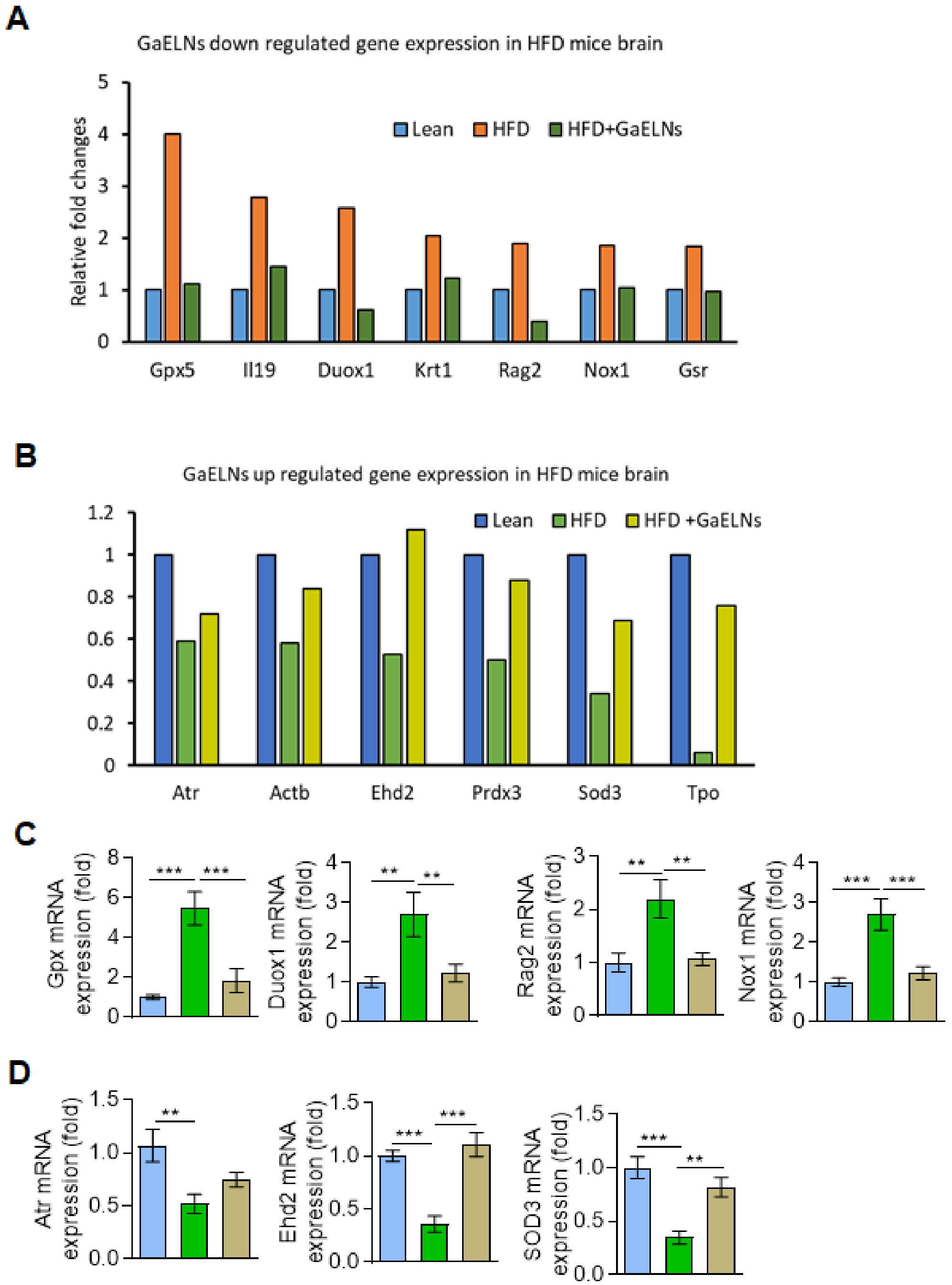

Figure S4: Reactive oxygen species gene array. (A-B). Total RNA isolated from brain of lean, HFD and GaELNs treated HFD fed mice was subjected to ROS gene array. (A). Down regulated genes. (B). Up regulated genes. (C-D). Total RNA isolated from lean, HFD and GaELNs treated HFD mice brain was subjected to real-time PCR form Gpx, Duox1, Rag2, Nox1, Atr, Ehd2 and Sod3 mRNA expression. The mRNA level was normalized by actin mRNA expression.

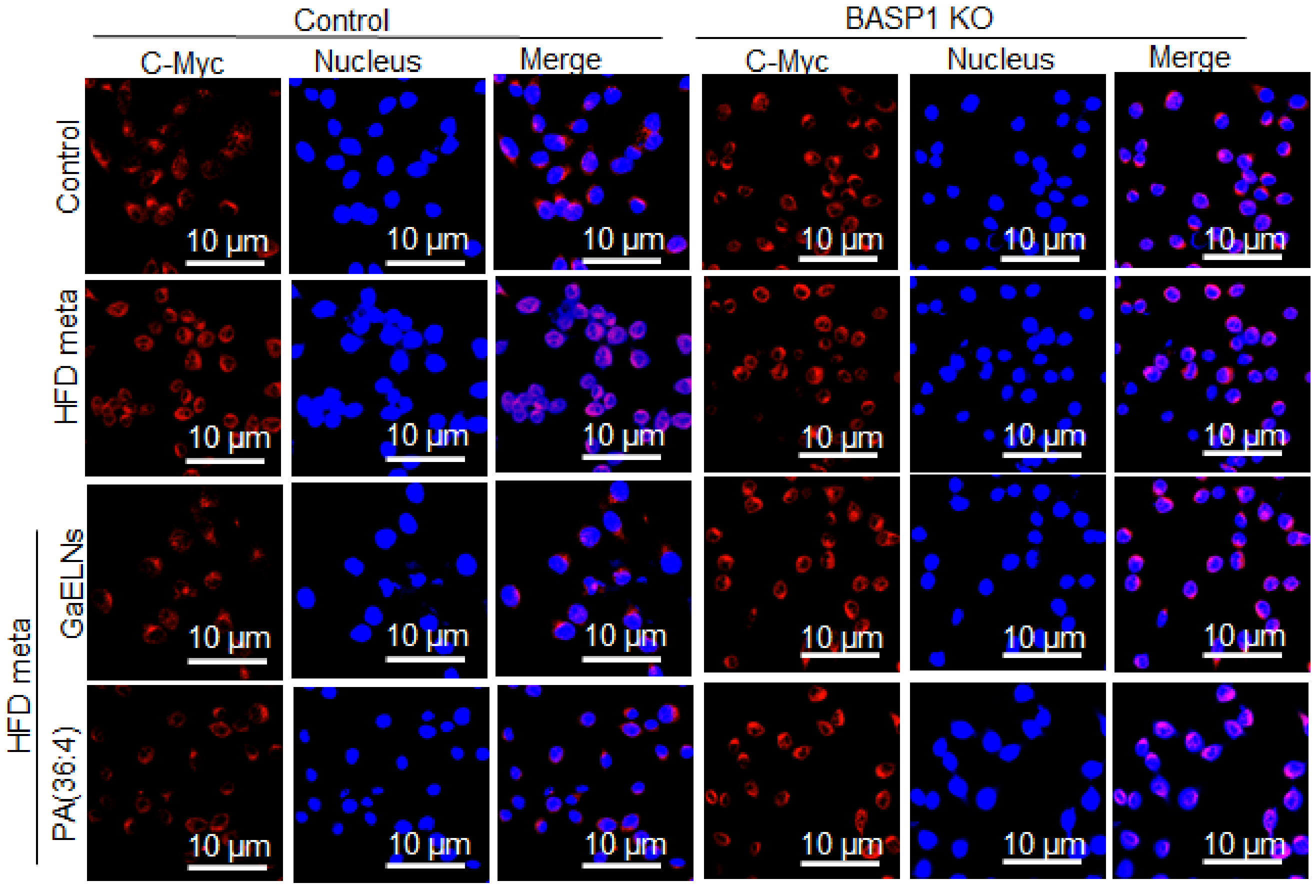

Figure S5: c-Myc localization in BASP1 knockout microglial cells. BV2 WT and BASP1 knockout cells were treated with metabolites derived from HFD mice brains and treated for 24 h with GaELNs or PA (36:4). The localization of c-Myc was visualized by confocal microscopy.

Table S1: miRNA profile of GaELNs

RNA_ ID	No. of repeats	Sequence
bdi-miR166f	22079	UCUCGGACCAGGCUUCAUUCC
gma-miR166u	22078	UCUCGGACCAGGCUUCAUUC
sbi-miR166k	22041	UCGGACCAGGCUUCAUUCCU
aly-miR166a-3p	21928	GGAAUGUUGUCUGGCUCGAGG
gma-miR166p	21924	UCGGACCAGGCUUCAUUCCC
gma-miR166m	21917	CGGACCAGGCUUCAUUCCCC
bdi-miR166e-3p	21911	CUCGGACCAGGCUUCAUUCCC
aly-miR159a-3p	541	UUUGGAUUGAAGGGAGCUCUA
ptc-miR396g-5p	253	UUCCACGGCUUUCUUGAACUU
mtr-miR166c	155	UCGGACCAGGCUUCAUUCCUC
gma-miR6300	104	GUCGUUGUAGUAUAGUGG
aly-miR168a-5p	44	UCGCUUGGUGCAGGUCGGGAA
gma-miR168b	43	UCGCUUGGUGCAGGUCGGG
aly-miR396a-5p	20	UUCCACAGCUUUCUUGAACUG
gma-miR396h	20	UCCACAGCUUUCUUGAACUG
aly-miR166a-5p	7	GGAAUGUUGUCUGGCUCGAGG
gma-miR4995	7	AGGCAGUGGCUUGGUUAAGGG

Table S2: List of brain metabolites from lean, HFD and GaELNs treated HFD mice

Name	Lean	HFD-PBS	HFD + GELNs
(+/-)12(13)-DiHOME	1	0.24	0.68
(±)9-HODE;(±)13-HODE	1	1.79	1.00
1-Methylguanine	1	13.55	0.96
2'-Deoxyinosine	1	1.35	1.41
2-Oxobutyric acid	1	4.00	0.70
3-Indoxyl sulphate	1	11.44	0.99
3-Phenyllactic acid	1	0.17	0.22
4-Dodecylbenzenesulfonic acid	1	2.41	2.35
4-Indolecarbaldehyde	1	3.80	1.09
4-Oxoproline	1	4.63	1.03
4-Pyridoxic acid	1	1.64	0.28
5-Aminovaleric acid	1	1.93	0.51
6-Hydroxycaproic acid	1	0.33	0.02
Adenine	1	0.61	0.46
Adenosine	1	2.05	1.00
Alanine	1	1.73	0.73
alpha-Ketoglutarate	1	4.40	0.53
Arabitol	1	7.50	1.05
Asparagine	1	0.45	0.40
Azelaic acid	1	1.06	0.72
Benzoic acid	1	0.89	1.01
Cholic acid	1	8.38	0.79
cis-Aconitic acid	1	9.58	0.45
Citraconic acid	1	12.60	1.15
Citric acid	1	1.87	1.44
Citrulline	1	0.65	1.00
Deoxycholic acid	1	4.76	0.83
DL-4-Hydroxyphenyllactic acid	1	1.02	0.67
DL-Arginine	1	0.83	0.54
DL-p-Hydroxyphenyllactic acid	1	1.54	0.58
DL-β-Leucine	1	1.70	0.49
D-Saccharic acid	1	1.08	0.75
D-α-Hydroxyglutaric acid	1	1.29	1.06
Ethylmalonic acid	1	0.68	1.04
Fructose	1	1.75	0.30
Glucose	1	0.16	1.76
Glutamic acid	1	0.95	0.53
Glutamine	1	0.61	0.85

Glutaric acid	1	1.75	1.02
Glycyl-L-leucine	1	0.24	0.60
Guanine	1	2.08	0.87
Guanosine	1	9.23	1.60
Hexanoylglycine	1	2.92	1.13
Hippuric acid	1	7.74	0.62
Histidine	1	0.71	0.74
Hypoxanthine	1	0.42	1.29
Inosine	1	3.17	2.26
Isocitric acid	1	13.67	1.17
Itaconic acid	1	1.52	0.67
Kynurenic acid	1	11.04	0.82
Lactate	1	0.80	0.36
L-alpha-Amino-n-butyric acid	1	0.98	0.71
L-Ascorbic acid 2-sulfate	1	36.02	0.92
L-Aspartic acid	1	1.68	0.72
L-Cysteine-S-sulfate	1	3.36	1.04
Levulinic acid	1	19.26	1.10
Malate	1	0.07	0.60
Maleic acid	1	4.05	0.94
Malonic acid	1	0.83	1.10
Mannitol	1	10.06	1.36
Methionine	1	1.19	0.55
N-Acetylaspartic acid	1	0.63	1.64
N-Acetyl-D-Glucosamine	1	0.39	1.57
N-Acetyl-DL-glutamic acid	1	1.35	1.08
N-Acetyl-L-glutamine	1	7.97	0.83
N-Acetylvaline	1	12.47	0.89
N-Formylmethionine	1	0.35	0.37
Nicotinic acid	1	0.34	1.45
N-Isobutyrylglycine	1	54.36	0.04
N-Tigloylglycine	1	29.13	0.76
O-Acetylserine	1	0.64	0.35
Ornithine	1	0.67	0.98
Orotic acid	1	9.48	0.83
Oxalic acid	1	2.54	1.14
Phenobarbital	1	157.32	1.60
Phenylacetylglycine	1	71.09	1.49
Phenylalanine	1	0.75	0.48
Phenyllactic acid	1	0.28	0.35
Pseudouridine	1	14.27	1.16

Pyruvic acid	1	1.64	0.69
Ribose	1	0.86	0.97
Salicylic acid	1	2.85	1.21
Serine	1	1.91	0.42
Suberic acid	1	0.92	0.00
Succinic acid	1	0.06	0.84
Succinic semialdehyde	1	2.76	0.61
Sucrose	1	5.89	3.38
Tartaric acid	1	105.23	1.20
Taurine	1	2.37	1.29
Threonine	1	1.20	0.50
Thymidine	1	18.43	0.61
trans-Aconitic acid	1	6.42	1.33
Tryptophan	1	0.60	0.51
Tyrosine	1	0.85	0.51
U (Uridine)	1	0.35	3.38
Uracil	1	0.82	1.38
Uric acid	1	11.35	1.20
Urocanic acid	1	1.41	0.45
Xanthine	1	0.72	0.81
Xanthurenic acid	1	19.50	0.73
β-D-Glucopyranuronic acid	1	1.91	1.18
δ-Gluconic acid δ-lactone	1	0.00	0.00

Table S3: List of brain metabolites from IDO1$^{-/-}$ mice

Name	IDO1-KO Vs Lean-HFD	IDO1-KO-HFD/Lean-HFD
(2E)-2-(hydroxymethyl)-3-[3-oxo-5-(propan-2-yl)-1,3,4,5,6,7-hexahydro-2-benzofuran-4-yl]prop-2-enoic acid	1.18	1.26
(2E,4E)-N-(2-methylpropyl)dodeca-2,4-dienamide	0.96	1.06
(4aR,5R,6R)-6-hydroxy-4a,5-dimethyl-3-(prop-1-en-2-yl)-2,4a,5,6,7,8-hexahydronaphthalen-2-one	0.94	1.07
[3-({3-[(Cyclopropylmethyl)amino]-3-oxetanyl}methyl)-1,2-oxazol-5-yl]methanol	0.60	0.70
1-(3-phenylpropanoyl)-4-piperidinecarboxylic acid	0.91	2.87
1,5-Isoquinolinediol	1.19	1.16
1,5-Naphthalenediamine	0.88	0.81
11-piperidino-2,3-dihydro-1H-cyclopenta[4,5]pyrido[1,2-a]benzimidazole-4-carbonitrile	0.57	0.92
1-Methyl-L-histidine/3-Methyl-L-histidine	0.86	0.97
1-Methyl-L-histidine/3-Methyl-L-histidine*,*1-Methyl-L-histidine*,*3-Methyl-L-histidine	0.83	1.05
1-Vinylimidazole	0.28	0.94
2-(2-amino-3-methylbutanamido)-3-phenylpropanoic acid	0.59	0.75
2-(2-amino-3-methylbutanamido)-3-phenylpropanoic acid	6.61	1.11
2-(2-hydroxy-3-methylbutanamido)-4-methylpentanoic acid	0.96	1.00
2,2,6,6-Tetramethyl-1-piperidinol (TEMPO)	1.35	1.73
2,4-Quinolinediol	0.64	0.60
2,4-Xylidine	0.85	0.97
2,6-Diaminotoluene	0.31	0.74
2-[(2S,3R,4S,5R)-3,4-Dihydroxy-5-{[(isopropylcarbamoyl)amino]methyl}tetrahydro-2-furanyl]-N-(3-pyridinylmethyl)acetamide	1.02	1.14
2-[(3S)-1-Benzyl-3-pyrrolidinyl]-1,3-benzothiazole	0.91	0.97
2-[2-(2-acetamido-4-methylpentanamido)propanamido]-3-hydroxypropanoic acid	0.74	1.46
2-[4-(3-Amino-2-hydroxypropoxy)phenyl]acetamide	0.85	0.94
2-Amino-3-methoxybenzoic acid	0.79	1.37
2-Amino-4-methylpyrimidine	0.72	0.88
2'-Deoxyadenosine	0.82	1.01
2-Ethylamino-1-phenylbutane	1.55	1.75
2-Hydroxycinnamic acid	1.15	1.07
2-Hydroxyphenylalanine	0.83	1.23
2-Mercaptoethanol	0.10	1.16
2-methyl-2,3,4,5-tetrahydro-1,5-benzoxazepin-4-one	1.27	1.36
2-Naphthylamine	2.52	1.12
3,14-dihydro-15-keto-tetranor Prostaglandin E2	0.86	0.91

3-Aminosalicylic acid	1.06	1.64
3-Hydroxy-2-methylpyridine	7.28	0.77
3-methylpentyl]oxy}-4-oxobutanoic acid	1.77	1.34
3-oxoindane-1-carboxylic acid	1.11	1.37
4,7-dihydroxy-4-(hydroxymethyl)-3,4a,8,8-tetramethyl-1,4,4a,5,6,7,8,8a-octahydronaphthalen-1-one	0.93	1.08
4-{[5-(6-Hydroxy-5,5,8a-trimethyl-2-methylenedecahydro-1-naphthalenyl)-3-methylpentyl]oxy}-4-oxobutanoic acid	0.81	0.71
4-Amino-N'-hydroxypyrimidine-5-carboximidamide	0.92	1.46
4-cyclopropyl-6-methoxy-1,3,5-triazin-2-amine	1.02	1.07
4-Guanidinobutyric acid	0.75	0.86
4-Hydroxybenzaldehyde	1.04	1.00
4-Hydroxyindole	2.13	2.45
4-Methoxycinnamaldehyde	1.58	2.04
4-Methyl-5-thiazoleethanol	1.13	1.16
4-oxobutanoic acid	0.40	0.77
4-Phenylbutyric acid	0.70	0.78
4-Pyridineacetic acid	0.04	1.25
5-(2-Nitroprop-1-enyl)-1,3-benzodioxole	1.12	1.64
5-(5-hydroxy-3-methylpentyl)-1,4a-dimethyl-6-methylidene-decahydronaphthalene-1-carboxylic acid	0.86	0.94
5-(6-hydroxy-6-methyloctyl)-2,5-dihydrofuran-2-one	3.92	3.98
5,5-dimethyl-2-{[(2-phenylacetyl)amino]methyl}-1,3-thiazolane-4-carboxylic acid	0.86	0.90
5,5-dimethyl-2-{[(2-phenylacetyl)amino]methyl}-1,3-thiazolane-4-carboxylic acid*,*5,5-dimethyl-2-{[(2-phenylacetyl)amino]methyl}-1,3-thiazolane-4-carboxylic acid	0.58	0.82
5,8-dihydroxy-10-methyl-5,8,9,10-tetrahydro-2H-oxecin-2-one	0.57	0.84
5-[4-(phenylsulfonyl)phenyl]-1H-pyrazole	4.89	4.01
5-fluoro AKB48 N-(4-hydroxypentyl) metabolite	0.77	1.52
5-Fluoro CUMYL-P7AICA	0.72	0.45
5-hydroxy-6,7-dimethoxy-2-phenyl-4H-chromen-4-one	1.89	2.02
5-hydroxy-indolacetic acid	1.08	1.14
5-Hydroxyindole-3-acetic acid	2.20	0.40
5-Methylcytosine	0.70	0.44
5'-S-Methyl-5'-thioadenosine	0.67	1.55
6-Aminocaproic acid	1.12	0.59
6-Methoxyquinoline	0.30	2.23
6-Methylnicotinamide	1.16	92.40
6-Pentyl-2H-pyran-2-one	0.73	1.33
7-(tert-butyl)-4-imino-1,2,3,4,5,6-hexahydropyrido[3,4-d]pyridazine-1,5-dione	1.00	93.36
7-Methylguanine	0.93	0.99

7-Methylguanosine*,*Gm (2'-O-methylguanosine)	0.66	0.86
9,10,13-trihydroxyoctadeca-11,15-dienoic acid	1.14	1.09
Acetophenone	2.50	0.60
Acetylcholine	1.04	1.34
Acetyl-L-carnitine	0.61	0.92
Acetyl-β-methylcholine	0.84	0.59
Adenine	0.65	0.86
Adenosine	0.18	2.60
Ageratriol	31.53	1.37
Agmatine	1.78	3.71
Apigenin 7-O-glucuronide	0.56	1.88
Arginine	0.90	1.18
Aspartame	1.52	0.98
Aspartic acid	1.89	2.24
Atrazine	0.95	0.91
Betain	0.22	1.42
Beta-Lactose	0.95	1.21
Bilirubin	0.44	3.21
Biochanin A	0.10	1.19
Bis(2-ethylhexyl) phthalate	0.87	1.28
Bis(4-ethylbenzylidene)sorbitol	0.82	0.76
Bis(methylbenzylidene)sorbitol	2.46	3.60
BMK ethyl glycidate	0.45	0.94
Buflomedil	0.70	1.35
cAMP (3' 5')	1.42	1.15
Carvone	1.32	0.88
Choline	1.26	0.60
Chrysin	0.86	1.58
cis-4-Hydroxy-D-proline	0.65	0.39
Citrinin	1.34	1.20
Citrulline	1.35	1.07
Cortisone	0.37	5.87
Corymboside	1.06	14.63
Coumarin	1.97	0.39
Creatine	1.40	0.55
Creatinine	0.84	1.00
Cystine	0.94	0.99
Cytidine	0.96	1.01
Cytosine	2.03	0.70
Daidzin	0.98	0.35
DEET	1.51	1.01

Deoxyadenosine	0.49	0.75
Desthiobiotin	0.82	2.92
Dibenzylamine	1.75	2.82
Diethyl 5-amino-2-benzylidene-3-oxo-7-phenyl-2,3-dihydro-7H-pyrido[2,1-b][1,3]thiazole-6,8-dicarboxylate	0.71	9.18
Diethyleneglycol diacetate	1.65	0.97
Diglycidyl ether	0.91	1.48
Diisobutylphthalate	1.13	2.57
Dipropylene glycol dimethyl ether	0.36	1.77
DL-2-Aminobutyric acid	1.61	2.12
DL-Carnitine	0.02	3.97
DL-Stachydrine	0.26	0.68
Docosahexaenoic acid ethyl ester	1.94	0.96
Dodecamethylcyclohexasiloxane	1.04	1.40
Erucamide	0.29	1.69
ethyl 5-(dimethylamino)-2-(trifluoromethyl)[1,6]naphthyridine-3-carboxylate	0.91	2.57
Ethyl palmitoleate	0.46	0.92
Ethylenediaminetetraacetic acid (EDTA)	0.42	2.10
Formononetin	1.33	1.23
G (Guanosine)	0.81	0.79
Gabapentin	0.97	1.03
Galangin	0.12	0.67
gamma-Amino-n-butyric acid	1.33	1.56
Genistein	0.60	2.06
Glycerophosphocholine	0.38	3.19
Glycerophospho-N-palmitoyl ethanolamine	0.57	0.93
Glycitein	1.53	1.74
Guanine	1.58	1.22
Guanosine	3.72	0.45
Hexadecanamide	0.93	0.90
Histamine	1.20	1.09
Histidine	0.76	1.11
Hypoxanthine	1.13	1.92
indole	0.43	0.49
Indole-3-acetyl-L-aspartic acid	2.43	0.78
indole-3-propionic acid	0.60	0.32
Inosine	0.63	0.60
Isoamylamine	8.94	1.22
Isoleucine	1.20	1.36
Kanosamine	0.01	10.10
Ketamine	0.78	1.12

Kynurenic acid	0.04	0.08
Lactamide	2.76	3.65
L-alpha-Amino-n-butyric acid	0.91	1.06
L-Ergothioneine	1819.77	3661.36
Leucine	0.16	1.18
Leucylproline	1.68	2.16
L-Histidinol	1.12	0.73
Linoleoyl Ethanolamide	0.89	1.49
L-Methionine sulfoxide	0.66	0.79
L-Pipecolic acid	1.88	0.36
L-Pyroglutamic acid	7.53	0.33
Lysine	0.84	1.15
Maltotetraose	0.82	0.70
Maltotriose	1.59	1.22
Maltotriose	6.37	0.46
Melibiose	0.53	1.32
Mesalamine	1.27	1.22
mesityl (4-methylphenyl) sulfone	1.44	1.78
Mesterolone	0.02	1.51
Methionine	0.23	0.12
Methylimidazoleacetic acid	1.10	1.06
Metolachlor morpholinone	2.18	2.84
N-(4-cyano-1-phenyl-1H-pyrazol-5-yl)-2-(4-methylpiperazino)acetamide	0.34	1.86
N-(5-acetamidopentyl)acetamide	0.21	166.24
N-(8-methyl-8-azabicyclo[3.2.1]oct-3-yl)-4-nitrobenzamide	1.31	2.39
N,N'-Dicyclohexylurea	1.39	2.43
N,N-Dimethylaniline	0.41	0.78
N,N-Dimethylarginine	0.20	2.19
N,N-Dimethylarginine	0.89	1.07
N,N-Dimethylglycine	0.97	0.98
N-[2-Pyrrolidin-1-yl-5-(trifluoromethyl)phenyl]-2,3-dihydro-1-benzofuran-5-carboxamide	0.79	0.85
N'-[6-(tert-butyl)thieno[3,2-d]pyrimidin-4-yl]-4-methylbenzohydrazide	0.96	1.24
N1-[4-hydroxy-6-(methoxymethyl)pyrimidin-2-yl]acetamide	2.16	0.93
N1-Acetylspermine	2.55	1.25
N1-phenethylbenzene-1-carbothioamide	0.53	3.55
N6,N6,N6-Trimethyl-L-lysine	0.04	0.80
N6-Acetyl-L-lysine	0.59	1.13
N8-Acetylspermidine	0.38	0.40
N-Acetyl-1-aspartylglutamic acid	0.17	1.94

N-Acetyl-D-Glucosamine	0.88	2.63
N-Acetylhistamine	2.25	2.34
N-Acetylputrescine	0.71	0.97
N-Benzylformamide	1.37	3.39
Nicotinamide	0.75	0.92
Nicotinic acid	0.61	12.45
Nipecotic acid	0.75	1.24
Nipecotic acid	0.38	0.93
N-Methyl-2-pyrrolidone	0.57	2.82
Norketamine	0.16	0.63
NPK	0.73	0.89
Oleoyl ethanolamide	0.73	3.07
Olomoucine	1.46	2.94
Ornithine	2.17	1.36
Palmitic Acid	1.80	2.80
Palmitoyl ethanolamide	0.07	0.76
Palmitoylcarnitine	0.79	1.25
Paracetamol	0.45	1.41
Penicillin G	0.72	1.04
Phenacetin	0.45	8.37
Phenylacetylglycine	3.45	1.39
Phenylalanine	0.03	0.06
Pipecolinic acid	1.02	1.12
Proline	7.21	8.06
Prolylglycine	0.99	1.70
Putrescine	2.37	3.41
Pyridoxal	0.49	0.76
Pyridoxamine	0.16	1.36
Pyridoxine	3.90	6.25
S-Adenosylmethionine	0.54	1.71
Salsolinol	0.41	6.46
Schaftoside	0.90	0.95
Serine	2.67	9.10
Spermidine	1.37	2.23
Spermine	0.69	4.08
Stearamide	2.27	1.56
Taurine	2.71	2.08
Taurochenodeoxycholic acid	9.75	1.07
Taurocholic acid	0.88	1.47
tert-Butyl N-[1-(aminocarbonyl)-3-methylbutyl]carbamate	1.02	2.25
Thiamine	0.60	1.18

Threonine	0.76	1.46
Thymine	0.43	0.83
Tiglic acid	0.79	0.67
Triethyleneglycol bis(2-ethylhexanoate)	2.16	1.41
Trigonelline	0.71	2.52
Triisopropanolamine	0.05	1.08
Triphenyl phosphate	1.66	2.79
Triphenylphosphine oxide	24.18	63.23
Valylproline	0.09	0.48

Table S4: Lipid profile of GaELNs

Mass	Compound Formula	Compound Name	nmol per mg dry wt
926.6	C49H80O15	DGDG(34:6)	0.000
928.6	C49H82O15	DGDG(34:5)	0.011
930.6	C49H84O15	DGDG(34:4)	0.012
932.6	C49H86O15	DGDG(34:3)	4.342
934.6	C49H88O15	DGDG(34:2)	1.906
936.6	C49H90O15	DGDG(34:1)	2.549
954.6	C51H84O15	DGDG(36:6)	11.414
956.6	C51H86O15	DGDG(36:5)	11.637
958.6	C51H88O15	DGDG(36:4)	4.246
960.6	C51H90O15	DGDG(36:3)	1.344
962.6	C51H92O15	DGDG(36:2)	0.042
982.6	C53H88O15	DGDG(38:6)	0.458
984.6	C53H90O15	DGDG(38:5)	0.105
986.6	C53H92O15	DGDG(38:4)	0.057
988.7	C53H94O15	DGDG(38:3)	0.187
**Total DGDG**		**Total DGDG**	**38.310**
766.5	C43H72O10	MGDG(34:5)	0.002
768.5	C43H74O10	MGDG(34:4)	0.136
770.5	C43H76O10	MGDG(34:3)	1.011
772.6	C43H78O10	MGDG(34:2)	0.046
774.6	C43H80O10	MGDG(34:1)	0.693
792.5	C45H74O10	MGDG(36:6)	20.221
794.5	C45H76O10	MGDG(36:5)	25.958
796.6	C45H78O10	MGDG(36:4)	11.788
798.6	C45H80O10	MGDG(36:3)	0.874
800.6	C45H82O10	MGDG(36:2)	0.870
802.6	C45H84O10	MGDG(36:1)	0.000
820.6	C47H78O10	MGDG(38:6)	0.077
822.6	C47H80O10	MGDG(38:5)	0.129
824.6	C47H82O10	MGDG(38:4)	0.127
826.6	C47H84O10	MGDG(38:3)	0.000
**Total MGDG**		**Total MGDG**	**61.931**
738.5	C38H73O10P	PG(32:1)	0.104
740.5	C38H75O10P	PG(32:0)	4.204
762.5	C40H73O10P	PG(34:3)	9.339
764.5	C40H75O10P	PG(34:2)	58.456
766.5	C40H77O10P	PG(34:1)	1.050
768.5	C40H79O10P	PG(34:0)	1.765

784.5	C42H71O10P	PG(36:6)	0.108
786.5	C42H73O10P	PG(36:5)	1.044
788.5	C42H75O10P	PG(36:4)	3.057
790.5	C42H77O10P	PG(36:3)	0.324
792.5	C42H79O10P	PG(36:2)	0.816
794.6	C42H81O10P	PG(36:1)	0.151
**Total PG**		**Total PG**	**80.419**
500.3	C22H43O9P	LPG(16:1)	2.122
502.3	C22H45O9P	LPG(16:0)	21.099
524.3	C24H43O9P	LPG(18:3)	2.284
526.3	C24H45O9P	LPG(18:2)	7.121
528.3	C24H47O9P	LPG(18:1)	4.169
**Total LysoPG**		**Total LysoPG**	**36.795**
494.3	C24H48O7PN	LPC(16:1)	0.144
496.3	C24H50O7PN	LPC(16:0)	2.770
518.3	C26H48O7PN	LPC(18:3)	0.614
520.3	C26H50O7PN	LPC(18:2)	8.221
522.3	C26H52O7PN	LPC(18:1)	1.183
524.4	C26H54O7PN	LPC(18:0)	0.170
**Total LysoPC**		**Total LysoPC**	**13.102**
452.3	C21H42O7PN	LPE(16:1)	0.000
454.3	C21H44O7PN	LPE(16:0)	3.732
476.3	C23H42O7PN	LPE(18:3)	0.036
478.3	C23H44O7PN	LPE(18:2)	5.379
480.3	C23H46O7PN	LPE(18:1)	0.193
**Total LysoPE**		**Total LysoPE**	**9.339**
734.6	C40H80O8PN	PC(32:0)	0.679
754.5	C42H76O8PN	PC(34:4)	0.000
756.5	C42H78O8PN	PC(34:3)	29.068
758.6	C42H80O8PN	PC(34:2)	229.053
760.6	C42H82O8PN	PC(34:1)	22.596
778.5	C44H76O8PN	PC(36:6)	3.304
780.5	C44H78O8PN	PC(36:5)	33.899
782.6	C44H80O8PN	PC(36:4)	208.835
784.6	C44H82O8PN	PC(36:3)	36.945
786.6	C44H84O8PN	PC(36:2)	8.233
788.6	C44H86O8PN	PC(36:1)	2.537
806.6	C46H80O8PN	PC(38:6)	0.139
808.6	C46H82O8PN	PC(38:5)	0.477
810.6	C46H84O8PN	PC(38:4)	2.170
812.6	C46H86O8PN	PC(38:3)	2.844
814.6	C46H88O8PN	PC(38:2)	2.319

836.6	C48H86O8PN	PC(40:5)	0.015
838.6	C48H88O8PN	PC(40:4)	0.295
840.6	C48H90O8PN	PC(40:3)	2.434
842.7	C48H92O8PN	PC(40:2)	0.939
**Total PC**		**Total PC**	**586.780**
686.5	C37H68O8PN	PE(32:3)	0.024
688.5	C37H70O8PN	PE(32:2)	0.160
690.5	C37H72O8PN	PE(32:1)	0.140
692.5	C37H74O8PN	PE(32:0)	0.049
712.5	C39H70O8PN	PE(34:4)	0.031
714.5	C39H72O8PN	PE(34:3)	7.317
716.5	C39H74O8PN	PE(34:2)	99.799
718.5	C39H76O8PN	PE(34:1)	3.196
736.5	C41H70O8PN	PE(36:6)	0.176
738.5	C41H72O8PN	PE(36:5)	6.761
740.5	C41H74O8PN	PE(36:4)	48.702
742.5	C41H76O8PN	PE(36:3)	6.027
744.5	C41H78O8PN	PE(36:2)	1.421
746.6	C41H80O8PN	PE(36:1)	0.418
764.5	C43H74O8PN	PE(38:6)	0.036
766.5	C43H76O8PN	PE(38:5)	0.090
768.5	C43H78O8PN	PE(38:4)	0.417
770.6	C43H80O8PN	PE(38:3)	0.390
798.6	C45H84O8PN	PE(40:3)	0.147
800.6	C45H86O8PN	PE(40:2)	2.170
826.6	C47H88O8PN	PE(42:3)	0.185
828.6	C47H90O8PN	PE(42:2)	1.633
**Total PE**		Total PE	**179.290**
822.5	C41H73O13P	PI(32:3)	0.145
824.5	C41H75O13P	PI(32:2)	0.056
826.5	C41H77O13P	PI(32:1)	0.077
828.5	C41H79O13P	PI(32:0)	0.307
848.5	C43H75O13P	PI(34:4)	0.102
850.5	C43H77O13P	PI(34:3)	3.884
852.5	C43H79O13P	PI(34:2)	21.787
854.5	C43H81O13P	PI(34:1)	1.054
872.5	C45H75O13P	PI(36:6)	0.586
874.5	C45H77O13P	PI(36:5)	3.268
876.5	C45H79O13P	PI(36:4)	9.024
878.5	C45H81O13P	PI(36:3)	1.824
880.6	C45H83O13P	PI(36:2)	0.496
882.6	C45H85O13P	PI(36:1)	0.006

**Total PI**		**Total PI**	**42.616**
756.5	C40H70O10PN	PS(34:4)	0.003
758.5	C40H72O10PN	PS(34:3)	0.126
760.5	C40H74O10PN	PS(34:2)	1.091
762.5	C40H76O10PN	PS(34:1)	0.000
780.5	C42H70O10PN	PS(36:6)	0.024
782.5	C42H72O10PN	PS(36:5)	0.128
784.5	C42H74O10PN	PS(36:4)	0.257
786.5	C42H76O10PN	PS(36:3)	0.018
788.5	C42H78O10PN	PS(36:2)	0.045
812.5	C44H78O10PN	PS(38:4)	0.008
814.6	C44H80O10PN	PS(38:3)	0.023
816.6	C44H82O10PN	PS(38:2)	0.087
818.6	C44H84O10PN	PS(38:1)	0.009
840.6	C46H82O10PN	PS(40:4)	0.001
842.6	C46H84O10PN	PS(40:3)	0.166
844.6	C46H86O10PN	PS(40:2)	2.103
846.6	C46H88O10PN	PS(40:1)	0.091
868.6	C48H86O10PN	PS(42:4)	0.013
870.6	C48H88O10PN	PS(42:3)	0.143
872.6	C48H90O10PN	PS(42:2)	2.058
874.6	C48H92O10PN	PS(42:1)	0.025
898.6	C50H92O10PN	PS(44:3)	0.021
**Total PS**		**Total PS**	**6.440**
666.5	C35H69O8P	PA(32:0)	0.173
686.4	C37H65O8P	PA(34:4)	0.012
688.5	C37H67O8P	PA(34:3)	2.715
690.5	C37H69O8P	PA(34:2)	24.595
692.5	C37H71O8P	PA(34:1)	1.764
710.4	C39H65O8P	PA(36:6)	0.150
712.5	C39H67O8P	PA(36:5)	3.241
714.5	C39H69O8P	PA(36:4)	24.664
716.5	C39H71O8P	PA(36:3)	3.406
718.5	C39H73O8P	PA(36:2)	0.772
**Total PA**		**Total PA**	**61.492**
**Total Routine Polar**		**Total Routine Polar**	**1116.513**
**32:1**	C35H70O5N	16:0/16:1	14.446
**32:0**	C35H72O5N	16:0/16:0	12.797
**34:6**	C37H64O5N	18:3/16:3	0.454
**34:5**	C37H66O5N	18:3/16:2	0.586
		18:2/16:3	2.137
**34:4**	C37H68O5N	18:3/16:1	6.930

		18:2/16:2	5.823
		18:1/16:3	0.618
**34:3**	C37H70O5N	18:3/16:0	1242.029
		18:2/16:1	44.513
		18:1/16:2	1.681
		18:0/16:3	0.207
**34:2**	C37H72O5N	18:2/16:0	9349.055
		18:1/16:1	6.765
		18:0/16:2	0.518
**34:1**	C37H74O5N	18:1/16:0	451.192
		18:0/16:1	0.036
**34:7-O**	C37H62O6N	OPDA/16:3	1.032
		18:3/dnOPDA	3.616
**34:8-2O**	C37H60O7N	OPDA/dnOPDA	0.406
**36:6**	C39H68O5N	18:3/18:3	37.417
**36:5**	C39H70O5N	18:3/18:2	218.610
**36:4**	C39H72O5N	18:3/18:1	188.482
		18:2/18:2	989.359
**36:3**	C39H74O5N	18:3/18:0	23.185
		18:2/18:1	282.898
**36:2**	C39H76O5N	18:2/18:0	188.068
		18:1/18:1	20.956
**36:1**	C39H78O5N	18:1/18:0	9.998
**36:7-O**	C39H66O6N	OPDA/18:3	0.742
**36:8-2O**	C39H64O7N	OPDA/OPDA	0.659
		**Total DAG**	**13105.214**
818.7	C51H96O6N	TAG(48:3)	0.005
820.7	C51H98O6N	TAG(48:2)	0.030
822.8	C51H100O6N	TAG(48:1)	0.020
824.8	C51H102O6N	TAG(48:0)	0.010
842.7	C53H96O6N	TAG(50:5)	0.002
844.7	C53H98O6N	TAG(50:4)	0.035
846.8	C53H100O6N	TAG(50:3)	0.495
848.8	C53H102O6N	TAG(50:2)	1.514
850.8	C53H104O6N	TAG(50:1)	0.157
862.7	C55H92O6N	TAG(52:9)	0.045
864.7	C55H94O6N	TAG(52:8)	0.006
866.7	C55H96O6N	TAG(52:7)	0.001
868.7	C55H98O6N	TAG(52:6)	0.311
870.8	C55H100O6N	TAG(52:5)	2.659
872.8	C55H102O6N	TAG(52:4)	7.650

874.8	C55H104O6N	TAG(52:3)	0.864
876.8	C55H106O6N	TAG(52:2)	0.185
890.7	C57H96O6N	TAG(54:9)	0.021
892.7	C57H98O6N	TAG(54:8)	0.003
896.8	C57H102O6N	TAG(54:6)	0.003
898.8	C57H104O6N	TAG(54:5)	0.012
900.8	C57H106O6N	TAG(54:4)	0.063
902.8	C57H108O6N	TAG(54:3)	0.157
904.8	C57H110O6N	TAG(54:2)	0.053
920.8	C59H102O6N	TAG(56:8)	0.002
922.8	C59H104O6N	TAG(56:7)	0.001
928.8	C59H110O6N	TAG(56:4)	0.001
930.8	C59H112O6N	TAG(56:3)	0.009
932.9	C59H114O6N	TAG(56:2)	0.030
958.9	C61H116O6N	TAG(58:3)	0.001
960.9	C61H118O6N	TAG(58:2)	0.007
**Total NL273 TAG 16:0 acyl containing**		**Total NL273 TAG 16:0 acyl containing**	**14.351**
818.7	C51H96O6N	TAG(48:3)	0.002
824.8	C51H102O6N	TAG(48:0)	0.006
840.7	C53H94O6N	TAG(50:6)	0.007
842.7	C53H96O6N	TAG(50:5)	0.022
844.7	C53H98O6N	TAG(50:4)	0.034
846.8	C53H100O6N	TAG(50:3)	0.082
864.7	C55H94O6N	TAG(52:8)	0.001
866.7	C55H96O6N	TAG(52:7)	0.030
868.7	C55H98O6N	TAG(52:6)	0.725
870.8	C55H100O6N	TAG(52:5)	2.602
872.8	C55H102O6N	TAG(52:4)	0.644
874.8	C55H104O6N	TAG(52:3)	0.013
876.8	C55H106O6N	TAG(52:2)	0.001
890.7	C57H96O6N	TAG(54:9)	0.666
892.7	C57H98O6N	TAG(54:8)	2.501
894.8	C57H100O6N	TAG(54:7)	6.545
896.8	C57H102O6N	TAG(54:6)	1.849
898.8	C57H104O6N	TAG(54:5)	0.312
900.8	C57H106O6N	TAG(54:4)	0.052
902.8	C57H108O6N	TAG(54:3)	0.001
904.8	C57H110O6N	TAG(54:2)	0.005
920.8	C59H102O6N	TAG(56:8)	0.017
922.8	C59H104O6N	TAG(56:7)	0.074
924.8	C59H106O6N	TAG(56:6)	0.113

926.8	C59H108O6N	TAG(56:5)	0.045
928.8	C59H110O6N	TAG(56:4)	0.006
950.8	C61H108O6N	TAG(58:7)	0.001
952.8	C61H110O6N	TAG(58:6)	0.011
954.8	C61H112O6N	TAG(58:5)	0.018
956.9	C61H114O6N	TAG(58:4)	0.003
**Total NL295 TAG 18:3 acyl containing**		**Total NL295 TAG 18:3 acyl containing**	**16.389**
818.7	C51H96O6N	TAG(48:3)	0.008
820.7	C51H98O6N	TAG(48:2)	0.026
824.8	C51H102O6N	TAG(48:0)	0.001
840.7	C53H94O6N	TAG(50:6)	0.002
842.7	C53H96O6N	TAG(50:5)	0.020
844.7	C53H98O6N	TAG(50:4)	0.135
846.8	C53H100O6N	TAG(50:3)	0.227
848.8	C53H102O6N	TAG(50:2)	0.601
862.7	C55H92O6N	TAG(52:9)	0.031
866.7	C55H96O6N	TAG(52:7)	0.006
868.7	C55H98O6N	TAG(52:6)	0.220
870.8	C55H100O6N	TAG(52:5)	3.494
872.8	C55H102O6N	TAG(52:4)	12.307
874.8	C55H104O6N	TAG(52:3)	0.863
876.8	C55H106O6N	TAG(52:2)	0.078
890.7	C57H96O6N	TAG(54:9)	0.035
892.7	C57H98O6N	TAG(54:8)	1.262
894.8	C57H100O6N	TAG(54:7)	11.191
896.8	C57H102O6N	TAG(54:6)	30.621
898.8	C57H104O6N	TAG(54:5)	4.008
900.8	C57H106O6N	TAG(54:4)	0.717
902.8	C57H108O6N	TAG(54:3)	0.156
904.8	C57H110O6N	TAG(54:2)	0.021
920.8	C59H102O6N	TAG(56:8)	0.005
922.8	C59H104O6N	TAG(56:7)	0.055
924.8	C59H106O6N	TAG(56:6)	0.276
926.8	C59H108O6N	TAG(56:5)	0.479
928.8	C59H110O6N	TAG(56:4)	0.156
930.8	C59H112O6N	TAG(56:3)	0.021
932.9	C59H114O6N	TAG(56:2)	0.004
952.8	C61H110O6N	TAG(58:6)	0.018
954.8	C61H112O6N	TAG(58:5)	0.046
956.9	C61H114O6N	TAG(58:4)	0.090
958.9	C61H116O6N	TAG(58:3)	0.009

960.9	C61H118O6N	TAG(58:2)	0.005
**Total NL297 18:2 acyl containing**		**Total NL297 18:2 acyl containing**	**67.194**
818.7	C51H96O6N	TAG(48:3)	0.001
820.7	C51H98O6N	TAG(48:2)	0.006
822.8	C51H100O6N	TAG(48:1)	0.008
824.8	C51H102O6N	TAG(48:0)	0.001
840.7	C53H94O6N	TAG(50:6)	0.000
842.7	C53H96O6N	TAG(50:5)	0.001
844.7	C53H98O6N	TAG(50:4)	0.005
846.8	C53H100O6N	TAG(50:3)	0.016
848.8	C53H102O6N	TAG(50:2)	0.047
850.8	C53H104O6N	TAG(50:1)	0.075
862.7	C55H92O6N	TAG(52:9)	0.012
864.7	C55H94O6N	TAG(52:8)	0.006
868.7	C55H98O6N	TAG(52:6)	0.006
870.8	C55H100O6N	TAG(52:5)	0.102
872.8	C55H102O6N	TAG(52:4)	0.663
874.8	C55H104O6N	TAG(52:3)	1.074
876.8	C55H106O6N	TAG(52:2)	0.316
890.7	C57H96O6N	TAG(54:9)	0.020
892.7	C57H98O6N	TAG(54:8)	0.028
894.8	C57H100O6N	TAG(54:7)	0.461
896.8	C57H102O6N	TAG(54:6)	1.987
898.8	C57H104O6N	TAG(54:5)	3.146
900.8	C57H106O6N	TAG(54:4)	0.617
902.8	C57H108O6N	TAG(54:3)	0.198
904.8	C57H110O6N	TAG(54:2)	0.067
922.8	C59H104O6N	TAG(56:7)	0.001
924.8	C59H106O6N	TAG(56:6)	0.011
926.8	C59H108O6N	TAG(56:5)	0.045
928.8	C59H110O6N	TAG(56:4)	0.080
930.8	C59H112O6N	TAG(56:3)	0.039
954.8	C61H112O6N	TAG(58:5)	0.001
956.9	C61H114O6N	TAG(58:4)	0.008
958.9	C61H116O6N	TAG(58:3)	0.020
960.9	C61H118O6N	TAG(58:2)	0.001
**Total NL299 TAG 18:1 acyl containing**		**Total NL299 TAG 18:1 acyl containing**	**9.072**
822.8	C51H100O6N	TAG(48:1)	0.002
824.8	C51H102O6N	TAG(48:0)	0.002
848.8	C53H102O6N	TAG(50:2)	0.002

850.8	C53H104O6N	TAG(50:1)	0.007
862.7	C55H92O6N	TAG(52:9)	0.004
864.7	C55H94O6N	TAG(52:8)	0.001
866.7	C55H96O6N	TAG(52:7)	0.001
870.8	C55H100O6N	TAG(52:5)	0.001
872.8	C55H102O6N	TAG(52:4)	0.006
874.8	C55H104O6N	TAG(52:3)	0.035
876.8	C55H106O6N	TAG(52:2)	0.099
890.7	C57H96O6N	TAG(54:9)	0.003
892.7	C57H98O6N	TAG(54:8)	0.005
894.8	C57H100O6N	TAG(54:7)	0.006
896.8	C57H102O6N	TAG(54:6)	0.053
898.8	C57H104O6N	TAG(54:5)	0.153
900.8	C57H106O6N	TAG(54:4)	0.340
902.8	C57H108O6N	TAG(54:3)	0.062
904.8	C57H110O6N	TAG(54:2)	0.028
920.8	C59H102O6N	TAG(56:8)	0.001
926.8	C59H108O6N	TAG(56:5)	0.001
928.8	C59H110O6N	TAG(56:4)	0.003
930.8	C59H112O6N	TAG(56:3)	0.006
932.9	C59H114O6N	TAG(56:2)	0.003
958.9	C61H116O6N	TAG(58:3)	0.001
960.9	C61H118O6N	TAG(58:2)	0.002
**Total NL301 TAG 18:0 acyl containing**		**Total NL301 TAG 18:0 acyl containing**	**0.828**
818.7	C51H96O6N	TAG(48:3)	0.001
820.7	C51H98O6N	TAG(48:2)	0.001
822.8	C51H100O6N	TAG(48:1)	0.001
824.8	C51H102O6N	TAG(48:0)	0.000
840.7	C53H94O6N	TAG(50:6)	0.001
842.7	C53H96O6N	TAG(50:5)	0.001
846.8	C53H100O6N	TAG(50:3)	0.000
848.8	C53H102O6N	TAG(50:2)	0.001
850.8	C53H104O6N	TAG(50:1)	0.001
862.7	C55H92O6N	TAG(52:9)	0.001
864.7	C55H94O6N	TAG(52:8)	0.001
866.7	C55H96O6N	TAG(52:7)	0.001
868.7	C55H98O6N	TAG(52:6)	0.002
870.8	C55H100O6N	TAG(52:5)	0.008
872.8	C55H102O6N	TAG(52:4)	0.003
874.8	C55H104O6N	TAG(52:3)	0.002
876.8	C55H106O6N	TAG(52:2)	0.005

890.7	C57H96O6N	TAG(54:9)	0.002
892.7	C57H98O6N	TAG(54:8)	0.001
894.8	C57H100O6N	TAG(54:7)	0.004
896.8	C57H102O6N	TAG(54:6)	0.007
898.8	C57H104O6N	TAG(54:5)	0.005
900.8	C57H106O6N	TAG(54:4)	0.039
902.8	C57H108O6N	TAG(54:3)	0.122
904.8	C57H110O6N	TAG(54:2)	0.028
920.8	C59H102O6N	TAG(56:8)	0.001
922.8	C59H104O6N	TAG(56:7)	0.020
924.8	C59H106O6N	TAG(56:6)	0.105
926.8	C59H108O6N	TAG(56:5)	0.276
928.8	C59H110O6N	TAG(56:4)	0.059
930.8	C59H112O6N	TAG(56:3)	0.027
932.9	C59H114O6N	TAG(56:2)	0.028
952.8	C61H110O6N	TAG(58:6)	0.003
954.8	C61H112O6N	TAG(58:5)	0.014
956.9	C61H114O6N	TAG(58:4)	0.034
958.9	C61H116O6N	TAG(58:3)	0.020
**Total TAG 20:1 acyl containing**		**Total TAG 20:1 acyl containing**	**0.825**

Table S5: Lipid profile of Band-3

Mass	Compound Formula	Compound Name	nmol per mg dry wt
954.6	C51H84O15	DGDG(36:6)	0.071
**Total DGDG**		**Total DGDG**	**0.071**
740.5	C38H75O10P	PG(32:0)	0.948
760.5	C40H71O10P	PG(34:4)	0.155
762.5	C40H73O10P	PG(34:3)	5.996
764.5	C40H75O10P	PG(34:2)	41.851
786.5	C42H73O10P	PG(36:5)	0.318
788.5	C42H75O10P	PG(36:4)	3.503
792.5	C42H79O10P	PG(36:2)	0.186
**Total PG**		**Total PG**	**52.959**
502.3	C22H45O9P	LPG(16:0)	0.062
526.3	C24H45O9P	LPG(18:2)	0.039
**Total LysoPG**		**Total LysoPG**	**0.101**
734.6	C40H80O8PN	PC(32:0)	0.335
754.5	C42H76O8PN	PC(34:4)	0.015
756.5	C42H78O8PN	PC(34:3)	0.193
758.6	C42H80O8PN	PC(34:2)	1.002
760.6	C42H82O8PN	PC(34:1)	0.143
778.5	C44H76O8PN	PC(36:6)	0.000
780.5	C44H78O8PN	PC(36:5)	0.420
782.6	C44H80O8PN	PC(36:4)	1.133
784.6	C44H82O8PN	PC(36:3)	0.204
786.6	C44H84O8PN	PC(36:2)	0.000
788.6	C44H86O8PN	PC(36:1)	0.112
810.6	C46H84O8PN	PC(38:4)	0.023
**Total PC**		**Total PC**	**3.579**
690.5	C37H72O8PN	PE(32:1)	0.194
714.5	C39H72O8PN	PE(34:3)	0.018
716.5	C39H74O8PN	PE(34:2)	0.654
718.5	C39H76O8PN	PE(34:1)	0.030
740.5	C41H74O8PN	PE(36:4)	0.048
746.6	C41H80O8PN	PE(36:1)	0.061
**Total PE**		**Total PE**	**1.006**
850.5	C43H77O13P	PI(34:3)	0.025
852.5	C43H79O13P	PI(34:2)	0.064
854.5	C43H81O13P	PI(34:1)	0.015
872.5	C45H75O13P	PI(36:6)	0.005

**Total PI**		**Total PI**	**0.109**
790.6	C42H80O10PN	PS(36:1)	0.266
818.6	C44H84O10PN	PS(38:1)	0.016
846.6	C46H88O10PN	PS(40:1)	0.309
872.6	C48H90O10PN	PS(42:2)	0.027
874.6	C48H92O10PN	PS(42:1)	0.342
**Total PS**		**Total PS**	**0.961**
686.4	C37H65O8P	PA(34:4)	0.056
688.5	C37H67O8P	PA(34:3)	1.686
690.5	C37H69O8P	PA(34:2)	16.193
712.5	C39H67O8P	PA(36:5)	2.613
714.5	C39H69O8P	PA(36:4)	19.361
716.5	C39H71O8P	PA(36:3)	1.307
718.5	C39H73O8P	PA(36:2)	0.000
**Total PA**		**Total PA**	**41.216**

Table S6: GaELNs lipid binding protein in HFD mice brain

Accession	Description	Gene Name	MW [kDa]
Q64433	10 kDa heat shock protein, mitochondrial	Hspe1	11
Q9CQV8	14-3-3 protein beta/alpha	Ywhab	28.1
P61982	14-3-3 protein gamma	Ywhag	28.3
P68254	14-3-3 protein theta	Ywhaq	27.8
P63101	14-3-3 protein zeta/delta	Ywhaz	27.8
P16330	2',3'-cyclic-nucleotide 3'-phosphodiesterase	Cnp	47.1
P10852	4F2 cell-surface antigen heavy chain	Slc3a2	58.3
P63038	60 kDa heat shock protein, mitochondrial	Hspd1	60.9
Q9DCD0	6-phosphogluconate dehydrogenase, decarboxylating	Pgd	53.2
Q99KI0	Aconitate hydratase, mitochondrial	Aco2	85.4
P40124	Adenylyl cyclase-associated protein 1	Cap1	51.5
Q9CYT6	Adenylyl cyclase-associated protein 2	Cap2	52.8
Q9EPJ9	ADP-ribosylation factor GTPase-activating protein 1	Arfgap1	45.3
Q61282	Aggrecan core protein	Acan	221.8
A2ASQ1	Agrin	Agrn	207.4
Q9JII6	Aldo-keto reductase family 1 member A1	Akr1a1	36.6
Q9QYC0	Alpha-adducin	Add1	80.6
P17182	Alpha-enolase	Eno1	47.1
P46660	Alpha-internexin	Ina	55.3
Q9DBG3	AP-2 complex subunit beta	Ap2b1	104.5
P84091	AP-2 complex subunit mu	Ap2m1	49.6
Q3UHJ0	AP2-associated protein kinase 1	Aak1	103.3
P05201	Aspartate aminotransferase, cytoplasmic	Got1	46.2
P05202	Aspartate aminotransferase, mitochondrial	Got2	47.4
P56480	ATP synthase subunit beta, mitochondrial	Atp5f1b	56.3
Q9Z2H5	Band 4.1-like protein 1	Epb41l1	98.3
Q9WV92	Band 4.1-like protein 3	Epb41l3	103.3
P18572	Basigin	Bsg	42.4
Q91XV3	Brain acid soluble protein 1	Basp1	22.1
Q61361	Brevican core protein	Bcan	95.8
P11798	Calcium/calmodulin-dependent protein kinase type II subunit alpha	Camk2a	54.1
P05132	cAMP-dependent protein kinase catalytic subunit alpha	Prkaca	40.5
P00920	Carbonic anhydrase 2	Ca2	29
P24270	Catalase	Cat	59.8
Q5M8N0	CB1 cannabinoid receptor-interacting protein 1	Cnrip1	18.6
Q8R5M8	Cell adhesion molecule 1	Cadm1	49.8
Q8BLQ9	Cell adhesion molecule 2	Cadm2	47.5

Q6ZQ06	Centrosomal protein of 162 kDa	Cep162	160.8
Q61548	Clathrin coat assembly protein AP180	Snap91	91.8
Q68FD5	Clathrin heavy chain 1	Cltc	191.4
P18760	Cofilin-1	Cfl1	18.5
P60824	Cold-inducible RNA-binding protein	Cirbp	18.6
P12960	Contactin-1	Cntn1	113.3
Q04447	Creatine kinase B-type	Ckb	42.7
P30275	Creatine kinase U-type, mitochondrial	Ckmt1	47
P97315	Cysteine and glycine-rich protein 1	Csrp1	20.6
Q61753	D-3-phosphoglycerate dehydrogenase	Phgdh	56.5
Q61495	Desmoglein-1-alpha	Dsg1a	114.5
E9Q557	Desmoplakin	Dsp	332.7
P97427	Dihydropyrimidinase-related protein 1	Crmp1	62.1
O08553	Dihydropyrimidinase-related protein 2	Dpysl2	62.2
Q62188	Dihydropyrimidinase-related protein 3	Dpysl3	61.9
Q811Q4	Disintegrin and metalloproteinase domain-containing protein 29	Adam29	86.4
P39053	Dynamin-1	Dnm1	97.7
O55176	E3 ubiquitin-protein ligase Praja-1	Pja1	63.9
P10126	Elongation factor 1-alpha 1	Eef1a1	50.1
P20029	Endoplasmic reticulum chaperone BiP	Hspa5	72.4
Q5EBJ4	Ermin	Ermn	32.1
P43006	Excitatory amino acid transporter 2	Slc1a2	62
G3X9C2	F-box only protein 50	Nccrp1	30.4
P05064	Fructose-bisphosphate aldolase A	Aldoa	39.3
P05063	Fructose-bisphosphate aldolase C	Aldoc	39.4
Q3UNH4	G protein-regulated inducer of neurite outgrowth 1	Gprin1	95.4
P17183	Gamma-enolase	Eno2	47.3
P26443	Glutamate dehydrogenase 1, mitochondrial	Glud1	61.3
P15105	Glutamine synthetase	Glul	42.1
P16858	Glyceraldehyde-3-phosphate dehydrogenase	Gapdh	35.8
Q8CI94	Glycogen phosphorylase, brain form	Pygb	96.7
P08752	Guanine nucleotide-binding protein G(i) subunit alpha-2	Gnai2	40.5
P62874	Guanine nucleotide-binding protein G(I)/G(S)/G(T) subunit beta-1	Gnb1	37.4
P62880	Guanine nucleotide-binding protein G(I)/G(S)/G(T) subunit beta-2	Gnb2	37.3
P18872	Guanine nucleotide-binding protein G(o) subunit alpha	Gnao1	40.1
P63094	Guanine nucleotide-binding protein G(s) subunit alpha isoforms short	Gnas	45.6
P62881	Guanine nucleotide-binding protein subunit beta-5	Gnb5	43.5
Q61316	Heat shock 70 kDa protein 4	Hspa4	94.1
P48722	Heat shock 70 kDa protein 4L	Hspa4l	94.3

P63017<br>P11499	Heat shock cognate 71 kDa protein<br>Heat shock protein HSP 90-beta	Hspa8<br>Hsp90ab1	70.8<br>83.2
P01942	Hemoglobin subunit alpha	Hba	15.1
Q640R3	Hepatocyte cell adhesion molecule	Hepacam	46.3
Q8BG05	Heterogeneous nuclear ribonucleoprotein A3	Hnrnpa3	39.6
O88569	Heterogeneous nuclear ribonucleoproteins A2/B1	Hnrnpa2b1	37.4
P17095	High mobility group protein HMG-I/HMG-Y	Hmga1	11.6
Q64523	Histone H2A type 2-C	H2ac20	14
Q64475	Histone H2B type 1-B	H2bc3	13.9
Q9ESM3	Hyaluronan and proteoglycan link protein 2	Hapln2	37.9
Q8R366	Immunoglobulin superfamily member 8	Igsf8	65
Q9D6R2	Isocitrate dehydrogenase [NAD] subunit alpha, mitochondrial	Idh3a	39.6
O88935-1	Isoform Ib of Synapsin-1	Syn1	70
Q02257	Junction plakoglobin	Jup	81.7
Q9JIT0	Limb region 1 protein	Lmbr1	55.1
Q8BLK3	Limbic system-associated membrane protein	Lsamp	38.1
P06151	L-lactate dehydrogenase A chain	Ldha	36.5
P16125	L-lactate dehydrogenase B chain	Ldhb	36.5
Q3U2K5	Lysine-specific demethylase 4D	Kdm4d	57.2
P17897	Lysozyme C-1	Lyz1	16.8
P14152	Malate dehydrogenase, cytoplasmic	Mdh1	36.5
P08249	Malate dehydrogenase, mitochondrial	Mdh2	35.6
Q99M71	Mammalian ependymin-related protein 1	Epdr1	25.5
Q9QYR6	Microtubule-associated protein 1A	Map1a	300
P14873	Microtubule-associated protein 1B	Map1b	270.1
P20357	Microtubule-associated protein 2	Map2	199
P27546	Microtubule-associated protein 4	Map4	117.4
Q7TSJ2	Microtubule-associated protein 6	Map6	96.4
Q8BGA9	Mitochondrial inner membrane protein OXA1L	Oxa1l	48.2
O08539	Myc box-dependent-interacting protein 1	Bin1	64.4
P04370	Myelin basic protein	Mbp	27.2
Q61885	Myelin-oligodendrocyte glycoprotein	Mog	28.3
P70441	Na(+)/H(+) exchange regulatory cofactor NHE-RF1	Slc9a3r1	38.6
Q9Z1P6	NADH dehydrogenase [ubiquinone] 1 alpha subcomplex subunit 7	Ndufa7	12.6
P13595	Neural cell adhesion molecule 1	Ncam1	119.4
P55066	Neurocan core protein	Ncan	137.1
Q810U3	Neurofascin	Nfasc	137.9
P19246	Neurofilament heavy polypeptide	Nefh	116.9
P08551	Neurofilament light polypeptide	Nefl	61.5
Q80Z24	Neuronal growth regulator 1	Negr1	37.9
P35802	Neuronal membrane glycoprotein M6-a	Gpm6a	31.1

P35803<br>P97300	Neuronal membrane glycoprotein M6-b<br>Neuroplastin	Gpm6b<br>Nptn	36.2<br>44.3
Q99PJ0	Neurotrimin	Ntm	38
P18608	Non-histone chromosomal protein HMG-14	Hmgn1	10.1
Q9CZ44	NSFL1 cofactor p47	Nsfl1c	40.7
Q80XU3	Nuclear ubiquitous casein and cyclin-dependent kinase substrate 1	Nucks1	26.3
Q01768	Nucleoside diphosphate kinase B	Nme2	17.4
P17742	Peptidyl-prolyl cis-trans isomerase A	Ppia	18
P35700	Peroxiredoxin-1	Prdx1	22.2
O70172	Phosphatidylinositol 5-phosphate 4-kinase type-2 alpha	Pip4k2a	46.1
Q9D0F9	Phosphoglucomutase-1	Pgm1	61.4
Q9DBJ1	Phosphoglycerate mutase 1	Pgam1	28.8
Q6Q477	Plasma membrane calcium-transporting ATPase 4	Atp2b4	133
P05622	Platelet-derived growth factor receptor beta	Pdgfrb	122.7
P62962	Profilin-1	Pfn1	14.9
Q9JJV2	Profilin-2	Pfn2	15
Q9R1P4	Proteasome subunit alpha type-1	Psma1	29.5
Q3U3W5	Protein arginine N-methyltransferase 9	Prmt9	94.2
Q4VAA2	Protein CDV3	Cdv3	29.7
P63318	Protein kinase C gamma type	Prkcg	78.3
Q62433	Protein NDRG1	Ndrg1	43
Q9QYG0	Protein NDRG2	Ndrg2	40.8
Q3UM45	Protein phosphatase 1 regulatory subunit 7	Ppp1r7	41.3
Q8K1S6	Protein spire homolog 2	Spire2	80.2
Q8K183	Pyridoxal kinase	Pdxk	35
P52480	Pyruvate kinase PKM	Pkm	57.8
P50396	Rab GDP dissociation inhibitor alpha	Gdi1	50.5
P47708	Rabphilin-3A	Rph3a	75.4
P26043	Radixin	Rdx	68.5
Q8K386	Ras-related protein Rab-15	Rab15	24.3
Q8BHD0	Ras-related protein Rab-39A	Rab39a	25
P63011	Ras-related protein Rab-3A	Rab3a	25
P35276	Ras-related protein Rab-3D	Rab3d	24.4
B9EKR1	Receptor-type tyrosine-protein phosphatase zeta	Ptprz1	254.2
P70335	Rho-associated protein kinase 1	Rock1	158.1
A2BE28	Ribosomal biogenesis protein LAS1L	Las1l	89.4
O89086	RNA-binding protein 3	Rbm3	16.6
P56959	RNA-binding protein FUS	Fus	52.6
Q8C1B7	Septin-11	Septin11	49.7
Q9R1T4	Septin-6	Septin6	49.6
Q76MZ3	Serine/threonine-protein phosphatase 2A 65 kDa regulatory	Ppp2r1a	65.3

P31650	subunit A alpha isoform<br>Sodium- and chloride-dependent GABA transporter 3	Slc6a11	69.9
Q8VDN2	Sodium/potassium-transporting ATPase subunit alpha-1	Atp1a1	112.9
Q6PIE5	Sodium/potassium-transporting ATPase subunit alpha-2	Atp1a2	112.1
Q6PIC6	Sodium/potassium-transporting ATPase subunit alpha-3	Atp1a3	111.6
P14094	Sodium/potassium-transporting ATPase subunit beta-1	Atp1b1	35.2
P14231	Sodium/potassium-transporting ATPase subunit beta-2	Atp1b2	33.3
Q62261	Spectrin beta chain, non-erythrocytic 1	Sptbn1	274.1
P54227	Stathmin	Stmn1	17.3
P08228	Superoxide dismutase [Cu-Zn]	Sod1	15.9
Q64332	Synapsin-2	Syn2	63.3
Q9JIS5	Synaptic vesicle glycoprotein 2A	Sv2a	82.6
Q8CHC4	Synaptojanin-1	Synj1	172.5
P60879	Synaptosomal-associated protein 25	Snap25	23.3
P46096	Synaptotagmin-1	Syt1	47.4
P61264	Syntaxin-1B	Stx1b	33.2
O08599	Syntaxin-binding protein 1	Stxbp1	67.5
Q8BYI9	Tenascin-R	Tnr	149.5
P01831	Thy-1 membrane glycoprotein	Thy1	18.1
P42669	Transcriptional activator protein Pur-alpha	Pura	34.9
Q01853	Transitional endoplasmic reticulum ATPase	Vcp	89.3
P40142	Transketolase	Tkt	67.6
P17751	Triosephosphate isomerase	Tpi1	32.2
Q7TQD2	Tubulin polymerization-promoting protein	Tppp	23.6
P97797	Tyrosine-protein phosphatase non-receptor type substrate 1	Sirpa	56.4
Q9R0P9	Ubiquitin carboxyl-terminal hydrolase isozyme L1	Uchl1	24.8
Q02053	Ubiquitin-like modifier-activating enzyme 1	Uba1	117.7
Q62059	Versican core protein	Vcan	366.6
P46460	Vesicle-fusing ATPase	Nsf	82.6
P20152	Vimentin	Vim	53.7
Q60932	Voltage-dependent anion-selective channel protein 1	Vdac1	32.3
P50516	V-type proton ATPase catalytic subunit A	Atp6v1a	68.3
P62814	V-type proton ATPase subunit B, brain isoform	Atp6v1b2	56.5
Q9Z1G3	V-type proton ATPase subunit C 1	Atp6v1c1	43.9
Q9WTT4	V-type proton ATPase subunit G 2	Atp6v1g2	13.6

Table S7: PA (36:4) binding protein in HFD mice brain

Accession	Description	Gene Name	MW [kDa]
P63101	14-3-3 protein zeta/delta	Ywhaz	27.8
P07356	Annexin A2	Anxa2	38.7
Q91XV3	Brain acid soluble protein 1	Basp1	22.1
P24270	Catalase	Cat	59.8
Q61495	Desmoglein-1-alpha	Dsg1a	114.5
E9Q557	Desmoplakin	Dsp	332.7
O08553	Dihydropyrimidinase-related protein 2	Dpysl2	62.2
P20029	Endoplasmic reticulum chaperone BiP	Hspa5	72.4
G3X9C2	F-box only protein 50	Nccrp1	30.4
P15105	Glutamine synthetase	Glul	42.1
Q8BG05	Heterogeneous nuclear ribonucleoprotein A3	Hnrnpa3	39.6
O88935-1	Isoform Ib of Synapsin-1	Syn1	70
P08730	Keratin, type I cytoskeletal 13	Krt13	47.7
Q9Z2K1	Keratin, type I cytoskeletal 16	Krt16	51.6
Q6IFX2	Keratin, type I cytoskeletal 42	Krt42	50.1
Q0VBK2	Keratin, type II cytoskeletal 80	Krt80	50.6
Q9QYR6	Microtubule-associated protein 1A	Map1a	300
P20357	Microtubule-associated protein 2	Map2	199
O08539	Myc box-dependent-interacting protein 1	Bin1	64.4
P17742	Peptidyl-prolyl cis-trans isomerase A	Ppia	18
P97350	Plakophilin-1	Pkp1	80.8
Q9R1P4	Proteasome subunit alpha type-1	Psma1	29.5
Q4VAA2	Protein CDV3	Cdv3	29.7
Q9QYG0	Protein NDRG2	Ndrg2	40.8
Q9JLF6	Protein-glutamine gamma-glutamyltransferase K	Tgm1	89.8
Q8CGC6	RNA-binding protein 28	Rbm28	84.2
Q8VDN2	Sodium/potassium-transporting ATPase subunit alpha-1	Atp1a1	112.9
P54227	Stathmin	Stmn1	17.3
O08599	Syntaxin-binding protein 1	Stxbp1	67.5
P68369	Tubulin alpha-1A chain	Tuba1a	50.1

Table S8. List of Primers used in this study

Name	Forward	Reverse
m-IDO1	TGAGCATTGCAAGGAAAGTG	TATAGGCCATCAGGCAGTCC
m-b3 tubulin	CTCAACCACCTTGTGTCTGC	GAAGAAGTGGAGACGTGGGA
m-OCT-4	CGGAAGAGAAAGCGAACTAGC	ATTGGCGATGTGAGTGATCTG
m-SOX2	ATGCACCGCTACGACGTGA	CTTTTGCACCCCTCCCATTT
m-Nestin	CAGCGTTGGAACAGAGGTTGG	TGGCACAGGTGTCTCAAGGGTAG
m-MAP2	AGATGCCAAGTAAGCCTGGT	GCAAATGGAACTGGAGGCAA
m-cGAS	GTTCAAACACAAGAAATGCACTG	GCTGACGGAGTACACAATCCT
m-STING	TGAAAGGCTCTTCATTGTCTCTT	TGGCATCTTCTGCTTCCTAGA
m-COI	GCCCCAGATATAGCATTCCC	GT TCATCCTGT TCCTGCTCC
m-AHR	GGCTTTCAGCAGTCTGATGTC	CATGAAAGAAGCGTTCTCTGG
m-TNF-α	TCTATGGCCCAGACCCTCAC	GACGGCAGAGAGGAGGTTGA
m-IL-6	GAGAGGAGACTTCACAGAGGATAC	GTACTCCAGAAGACCAGAGG
m-IL-4	GAGACTCTTTCGGGCTTTTC	TGATGCTCTTTAGGCTTTCCA
m-IFN-γ	CTTTGCAGCTCTTCCTCATGGCTGTTTCTG	TGACGCTTATGTTGTTGCTGATGGCCTG
m-IL-1α	ATCAGTACCTCACGGCTGCT	TGGGTATCTCAGGCATCTCC
m-Cyp1a2	AGTACATCTCCTTAGCCCCAG	GGTCCGGGTGGATTCTTCAG
m-Cyp1b1	CCACCAGCCTTAGTGCAGAC	GGCCAGGACGGAGAAGAGT
m-BASP1	AGGCAAGCTGAGCAAGAAGA	GCTTCTCCTCCGTGCTCTC
m-β-actin	TGTGATGGTGGGAATGGGTCAG	TTTGATGTCACGCACGATTTCC
m-GPX5	AGGCCGGAAAAGATGAAGAT	CCGCAATAGGTAGCCACATT
m-Duox1	AAGGGCTGAAGATGTGGATG	CCCTGGCTTTGGTGTAAGAA
m-Rag2	GAGATGTCCCTGAACCCAGA	AACATGGGGTAGGCAGTCAG
m-Nox1	TCCATTTCCTTCCTGGAGTG	CCCAACCAGTACAGCCACTT
m-Atr	CGGCTTTACAGGTCAGGAAG	CTGTTGAGCTTGGCATTGAA
m-Ehd	CTGAGGTCCTTCGCGTCTAC	TTGTTTTCCTTCCCGAACAC
m-Sod3	TCTGCAGGGTACAACCATCA	ACCTCCATCGGGTTGTAGTG
